# Comprehensive characterization of the chemical constituents in Yiganmingmu oral liquid and the absorbed prototypes in cynomolgus monkey plasma after oral administration by UPLC-Q-TOF-MS based on the self built components database

**DOI:** 10.1186/s13020-021-00443-0

**Published:** 2021-04-28

**Authors:** Wei Wei, Siwei Li, Linyou Cheng, Erwei Hao, Xiaotao Hou, Hua Zhou, Jiagang Deng, Xinsheng Yao

**Affiliations:** 1 College of Pharmacy and International Cooperative Laboratory of Traditional Chinese Medicine Modernization and Innovative Drug Development of Chinese Ministry of Education, Jinan University, Guangzhou, 510632 China; 2 Guangxi Key Laboratory of Efficacy Study on Chinese Materia Medica, Guangxi University of Chinese Medicine, Nanning, 530200 Guangxi China; 3 National and Region Joint Engineering Center for Anticancer Drug Development, Guangxi Hebabiz Pharmaceutical Co. Ltd., Qinzhou, 535000 China; 4 Faculty of Pharmacy, Guangxi University of Chinese Medicine, Nanning, 530200 China

**Keywords:** Yiganmingmu oral liquid, UPLC-Q-TOF-MS, Self built database, Chemical profile, Cynomolgus monkey

## Abstract

**Background:**

Yiganmingmu oral liquid (YGMM), a well known over-the-counter (OTC) drug in China, is composed of 12 types of valuable herbal medicines and has been widely used in clinical for the treatment of soreness and weakness of waist and knees, dizziness, memory loss, and fatigue. However, the chemical compositions of YGMM and its absorbed compounds in plasma remain unclear.

**Methods:**

Since chemical investigation is the first important step to reveal effects and action mechanisms of traditional Chinese medicine (TCM), in this study, based on the self built components database, systematic characterization of the chemical profile of YGMM in vitro was carried out by using a reliable UPLC-Q-TOF-MS method. Moreover, to obtain better understanding of the absorbed prototypes in plasma, serum pharmacochemistry analysis of YGMM after oral administration was conducted by using cynomolgus monkeys as animal model.

**Results:**

A total of 667 constituents from the 12 single herbal medicines were collected in the self built components database by searching the reported literatures, and 415 of them were initially screened as candidate compounds in YGMM by comparison of their experimental accurate mass measurements with those theoretical values. After that, 117 compounds including 17 phenolic acids, 25 flavonoids, 4 alkaloids, 10 phthalides, 5 monoterpenes, 8 triterpenoid saponins, 9 anthraquinones, and 39 other compounds, were unambiguously identified or tentatively characterized by analysing their MS/MS fragmentation patterns, and also by comparison with reference standards and those data reported in the literatures. 61 prototypes absorbed in plasma of cynomolgus monkey, including 13 phenolic acids, 21 flavonoids, 8 phthalides, 3 monoterpenes, 4 triterpenoid saponins, and 12 other compounds were tentatively assigned by serum pharmacochemistry analysis after oral administration.

**Conclusion:**

It was the first comprehensive analysis of chemical constituents of YGMM and prototypes in plasma, and the data analysis strategy developed in this study showed high efficiency in the structural elucidations. The results might provide scientific evidence for further research on material basis of YGMM.

**Supplementary Information:**

The online version contains supplementary material available at 10.1186/s13020-021-00443-0.

## Background

Traditional Chinese medicine (TCM) prescriptions has long been used in the treatment of complex and chronic diseases in China due to their high efficiency but relatively low toxicity [1]. According to the wholesome thought of Chinese medicine theory, TCM prescriptions collectively exerts therapeutic effects via multi-target by complex interactions among the complicated composition systems formed by different single herbal medicines in the prescription [2]. The inefficiency in the material foundation study of TCM has seriously restricted its development and modernization. In recent years, the combination of chemical component investigation in vitro and serum pharmacochemistry analysis in vivo has been widely accepted as an effective strategy to obtain a better understanding of the potential therapeutic material basis of TCM [3, 4]. In which, ultra-high-performance liquid chromatography quadrupole time of flight mass spectrometry (UPLC-Q-TOF-MS) was considered the most powerful analytical tool for chemical components characterization of TCM and biosamples, due to its high speed, wide measurable mass range, high ratio of resolution, and capacity for simultaneous qualitative analysis [5]. First recorded in the 2010 edition of Chinese Pharmacopoeia, UPLC-Q-TOF-MS have become an important and irreplaceable analytical method in the field of quality control of TCM, such as main components or trace substance determination, fingerprint determination, and toxic component control, etc. Besides, since the important role in the screening of the active components and the establishment of TCM standards, in the later editions of Chinese Pharmacopoeia, the use of UPLC-Q-TOF-MS has significantly increased.

**Fig. 1:**
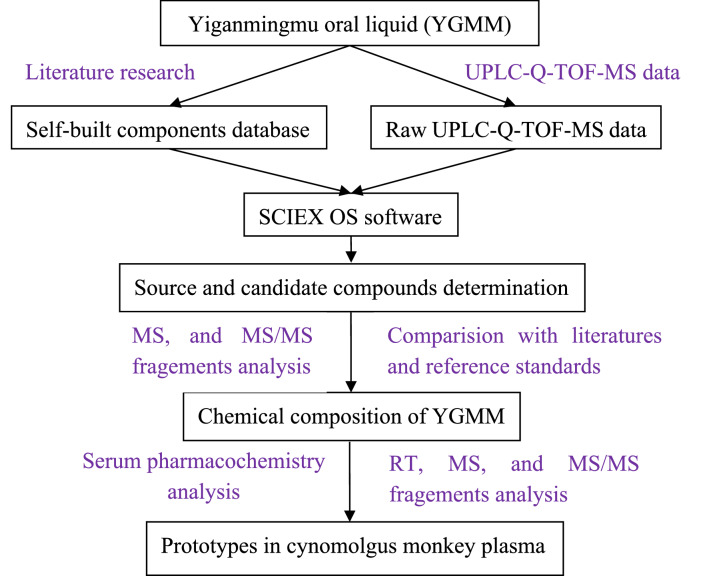
The data analysis strategy for identification of chemical constituents in YGMM and prototypes in cynomolgus monkey plasma

Yiganmingmu oral liquid (YGMM), a well known TCM prescription, is an over-the-counter (OTC) drug registered and approved by CFDA (Approval No. B20050056) for treating soreness and weakness of waist and knees, dizziness, memory loss, and fatigue [6]. On the basis of the two classical TCM formulas [7], Siwu Tang and Gugen Tang created by a famous doctor named Shiduo Chen in Qing dynasty, the medicinal herbs contained in the prescription of YGMM was extended to 12 flavors including *Rehmannia glutinosa* (RG, Shudihuang), *Angelica sinensis* (AS, Danggui), *Paeonia lactiflora* Pall (PL, Baishao), *Polygonatum odoratum* (PO, Yuzhu), *Ophiopogon japonicas* (OJ, Maidong), *Chrysanthemum morifolium* (CM, Juhua), *Ligusticum chuanxiong* (LC, Chuanxiong), *Anemone altaica* (AA, Jiujiechangpu), *Citrus reticulate* (CR, Chenpi), *Cassiae semen* (CS, Juemingzi), *Lycii fructus* (LF, Gouqizi), and *Bupleuri radix* (BR, Chaihu). Recently our research showed that YGMM possessed good hepatoprotective activity on isoniazid-rifampicin induced liver injuries in rats [6]. However, the pharmacodynamic material basis of YGMM is still unclear. Compared to the other animal models used in experiments, cynomolgus monkey is a more useful preclinical model due to their nonhuman primates and are more similar to humans in genetics and pathophysiology, and have been applied recently for the serum pharmacochemistry analysis of Yizhi Granule [8] by our group. In the present study, we established a comprehensive data analysis strategy for the chemical components identification of YGMM and the absorbed prototypical ingredients in the plasma of cynomolgus monkey after oral administration for the first time. The self built components database was helpful to enhance the efficiency of constituents characterization (Fig. 1), thus, a self built components database containing the reported chemical components of each individual medicinal herbs [9–46] of YGMM was used for the rapid screening and identification of chemical components in vitro and prototypes in vivo. This study might provide an useful analytical strategy for elucidating the material basis of YGMM and a promising experimental data for its material basis and quality control studies.

## Materials and methods

### Chemicals and materials

YGMM were provided by Guangxi Hebabiz Pharmaceutical Co., Ltd. (Lot No. 130418). 16 reference standards (purity ≥ 95%) including Z-Ligustilide, senkyunolide H, senkyunolide I, vanillic acid, gallic acid, 3-*O*-feruloylquinic acid, isochlorogenic acid A, kaempferitrin, mudanpioside D, cassiaside B2, ophiopogonin D, hesperetin, naringin, rutin, quercetin, and ophiopogonanoe B were all purchased from Chengdu Biopurify Phytochemicals Ltd. (Sichuan, China). LC–MS grade acetonitrile and methanol were purchased from Merck (Darmstadt, Germany). LC–MS grade formic acid was obtained from Sigma-Aldrich (Mo, USA). Ultrapure water was purified using a Milli-Q 89 water purification system (Millipore, Billerica, MA, USA). The other reagents were all analytical grade.

### Animals and drug administration

Male cynomolgus monkeys (7 years old, 7.0 ± 0.5 kg weight) were provided by Guangxi cynomolgus medicine applied engineering technology research center (Guangxi province), and were housed in an animal room individually with a suspended stainless steel cage at the environment condition set as follow: room temperature and relative humidity was kept with a 12 h dark/light cycle at 24–26 °C, and 50–70%, respectively. Certified primate pellet diet and clean water were provided every day. Fruits were supplemented regularly for nutrition as is standard practice. All experiments were conducted in accordance with the Regulations of Experimental Animal Administration issued by the State Commission of Science and Technology of the People’s Republic of China. Experimental animal protocols were approved by the Animal Ethics Committee of Guangxi University of Chinese Medicine, and all procedures were following the relevant regulations and guidelines.

### Preparation of sample solution

0.5 mL of YGMM was diluted with 2 mL methanol and the dilution was filtered through 0.22 µm filter membrane before LC–MS analysis. All samples were stored at 4 °C until use. Six male cynomolgus monkeys were fasted with only access to water for 12 h prior to the experiment. 30 mL of YGMM was orally administered to each monkey. At the time point of 0.5 h, 1 h, 2 h and 3 h after dosing via intragastric gavage, 1 mL blood was collected by venipuncture respectively and then centrifuged for 10 min at 3000 rpm/min at 4 °C to obtain the supernatant. The supernatant from different time points were mixed together to give the plasma sample and was frozen at − 80 °C before analysis. After reconstitution, 6 mL of acetonitrile was added to the plasma and vortex-mixed for 1 min and then was centrifuged at 12,000 rpm/min at 4 °C for 10 min. The supernatant was purified by solid-phase extraction and then dried under nitrogen gas at the temperature of 45 °C. The residues were dissolved in 2 mL of 50% methanol and then centrifuged at 12,000 rpm/min for 10 min at 4 °C. Sample solutions were filtered through 0.22 µm filter membrane and then 4 μL of the solution was injected into the LC–MS system for analysis. A certain amount of 16 reference standards were dissolved in methanol to obtain the standard solutions. Before LC–MS analysis, they were mixed together and then filtered through millipore filters to give the mixed standard solution.

### Chromatography and mass spectrometry conditions

The separation of the components in YGMM and biosamples were conducted on a ACQUITY UPLC BEH C18 column (2.1 mm × 100 mm, 1.7 μm, Waters Corporation, USA) using the mobile phase consisted of solvent A (HCOOH: H_2_O = 0.1: 100, v/v) and solvent B (CH_3_CN) on the Shimadzu Nexera Prominence liquid chromatogram system at the gradient eluting procedure optimized as follows: 0–25 min, 5–10% B; 25–40 min, 10–16% B; 40–50 min, 16–45% B, 50–57 min, 45–60% B, and 57–65 min, 100% B. The flow rate was set at 0.4 mL/min. The column and autosampler temperature were maintained at 40 °C and 4 °C, respectively. The inject volume for YGMM and mixed standard solution was 2 µL for each, and for the plasma sample was 4 µL.

Mass spectrometric detection was conducted on the AB SCIEX X500R quadrupole-time of flight (QTOF) coupled with high resolution mass spectrum (HRMS) (Applied Biosystems SCIEX, US) at full scan mode from *m/z* 100 to 2000 under ESI mode operating in both positive and negative modes. The MS, and MS/MS data of the compounds was acquired in the information-dependent acquirement (IDA) technology mode. The optimized parameters for IDA were set as follows: ion source gas 1 (GS1): 55 psi, ion source gas 2 (GS2): 55 psi, curtain gas: 35 psi, temperature: 600 °C, and CAD gas: 7. For TOF MS: mass range of the components, *m/z* 100–2000, declustering potential (DP): ± 80 V, collision energy (CE): ± 35 V, and CE spread: 0 V. For TOF MSMS: mass range of the fragments, *m/z* 100–2000, declustering potential (DP): 80 V, collision energy (CE): ± 35 V, and CE spread: 15 V, and accumulation time: 0.05 s. Data acquisition and analysis were controlled by SCIEX OS software (Ver. 1.3.1, AB SCIEX Co.).

### Self built components database of YGMM

The systematic information on chemical constituents isolated or identified from the 12 individual herbs in YGMM was collected and sorted out by retrieving the published literatures involving chemical constituents studies, fingerprint of medicinal materials studies, reviews, and master’s and doctoral dissertations, etc. As a result, a self built components database included compound name, and chemical formulas of each compound was established for further structural elucidation. All the compounds collected in the self built database were listed in Additional file 1: Table S1.

### The comprehensive research strategy and data processing

In order to rapidly characterize the chemical profile of YGMM, an investigate research strategy that integrated the UPLC-Q-TOF-MS method and self built components database was established. As shown in Fig. 1, firstly the chemical components of all the individual medical herbs were collected in a excel table to give the self built components database, which contained the compound name and chemical formula for each compound. Secondly, the raw MS data of YGMM was acquired using the established UPLC-Q-TOF-MS method. After that the excel table was imported into the SCIEX OS software, and data filtering and screening were automatically performed. Adducts including H^+^, Na^+^, and K^+^ were selected for positive mode, and for negative mode, adducts including Cl^+^, HCOO^+^ and H^−^ were selected. The extracted ion chromatograms (XIC) Width was set as 0.02 Da. The confidence levels of compounds for the qualitative rules were set as follow: mass accuracy tolerance set at ± 10 ppm, and combined score weight for it was 60%; different isotope ratio tolerance set at ± 10 ppm, and combined score weight for it was 40%. The third step of the data analysis strategy was to screen out the candidate compounds of YGMM and confirm the source of each component by using the above-mentioned screening rules. By analyzing the MS/MS fragmentation patterns, and also by comparison with standard compounds and those data reported in the literatures, the chemical components in YGMM were finally confirmed from the screened candidate compounds. Serum pharmacochemistry analysis of the prototypes in cynomolgus monkey plasma after oral administration was carried out by using the same UPLC-Q-TOF-MS method in IDA mode. XIC mode was applied to extract the prototypes by comparison the retention time, MS, and MS/MS fragments data with those identified components in YGMM.

## Results

**Fig. 2:**
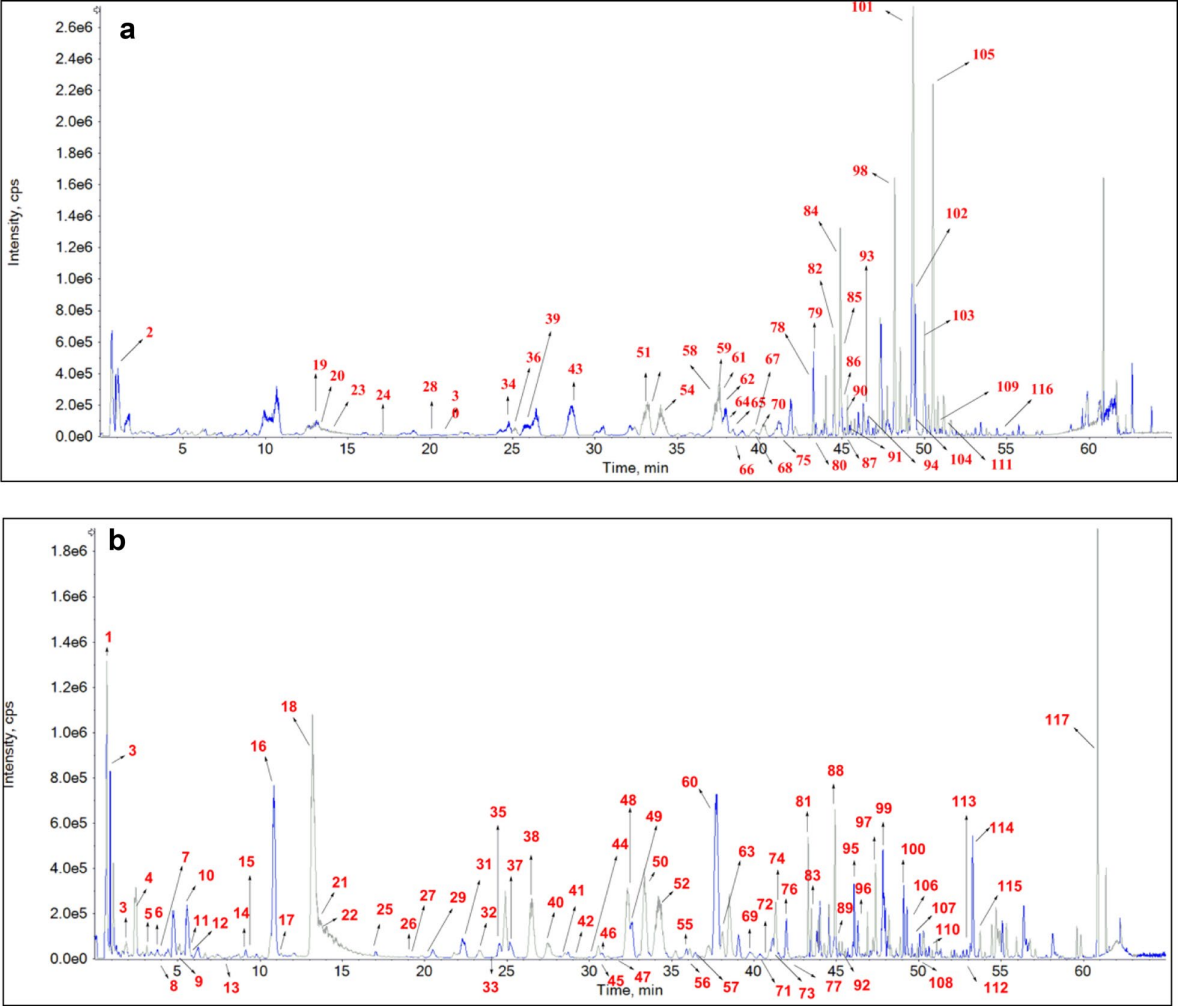
The BPC of YGMM in positive (**a**) and negative (**b**) ion modes

**Fig. 3:**
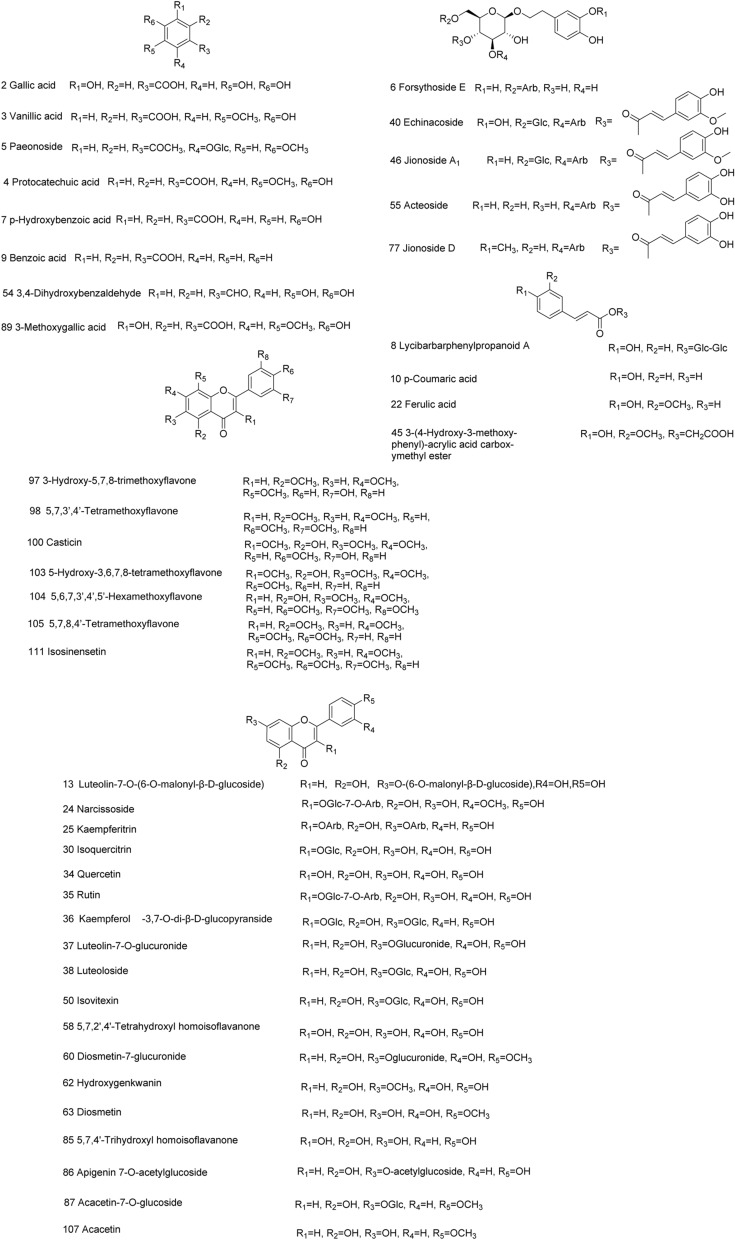
The chemical structures of main identified constituents in YGMM

By searching the published literatures of 12 single herb medicines, a total of 667 components were collected in the self built components database (Additional file 1: Table S1). The solution of YGMM was analyzed in both positive and negative ion modes by using UPLC-Q-TOF-MS in the IDA mode. The base peak chromatograms (BPC) in positive and negative modes of YGMM are showed in Fig. 2. Using the established data analysis strategy, 415 compounds were initially screened out as candidate compounds in YGMM, as shown in Additional file 1: Table S2. A total of 117 compounds and 61 prototypes were unambiguously identified or tentatively characterized in YGMM and plasma of cynomolgus monkey, respectively, as shown in Table 1, which included the information of retention time, molecular formula, mass weight, mass error, and main fragment of each compound. The chemical structures of the main compounds in YGMM were shown in Fig. 3, and all the compounds identified were shown in Additional file 1: Fig.S1.

**Table 1:** Identification of chemical constituents of YGMM and prototypes in cynomolgus monkeys plasma

No.	Retention time	Adduct/charge	Precursor mass	Found at mass	Mass error (ppm)	Formula	Compound name	MS/MS fragment ions (*m/z*)	Source	Prototypes
1	0.98	[M−H]^−^	117.0193	117.0193	− 0.3	C_4_H_6_O_4_	Succinic acid	117.0910 [M−H]^−^	AA	
2^a^	1.06	[M+H]^+^	171.0290	171.029	1.3	C_7_H_6_O_5_	Gallic acid	153.0196 [M+H–H_2_O]^+^, 135.0087 [M+H–2H_2_O]^+^, 109.026 [M+H–CO_2_]^+^	BC, RP, LC	P
3^a^	1.85	[M−H]^−^	167.0350	167.0351	0.6	C_8_H_8_O_4_	Vanillic acid	167.0360 [M−H]^−^, 123.0445 [M−H–CO_2_]^−^	AS, LC	P
4	1.99	[M−H]^−^	153.0193	153.0195	0.8	C_7_H_6_O_4_	Protocatechuic acid	109.0293 [M−H–CO_2_]^−^	RG	
5	2.01	[M+HCOO]^−^	373.1140	373.1141	0.3	C_15_H_20_O_8_	Paeonoside	373.1300 [M+HCOO]^−^, 329.0862 [M+HCOO–CO_2_]^−^	RP	P
6	3.06	[M−H]^−^	461.1664	461.1663	− 0.4	C_20_H_30_O_12_	Forsythoside E	461.1664 [M−H]^−^, 315.1104 [M−H–Rha]^−^, 135.0445 [M−H–Rha–Glc–H_2_O]^−^	RG	P
7	3.13	[M−H]^−^	137.0244	137.0243	− 0.9	C_7_H_6_O_3_	*p*-Hydroxybenzoicacid	137.0241 [M−H]^−^, 119.0142 [M−H–H_2_O]^−^	LC	P
8	3.77	[M+HCOO]^−^	533.1512	533.1517	0.9	C_21_H_28_O_13_	Lycibarbarphenylpropanoid A	487.1468 [M−H]−, 163.0396 [M−H–2Glc]^−^, 119.0500 [M−H–2Glc–CO_2_]^−^	LB	
9	5.34	[M+HCOO]^−^	167.0350	167.0348	− 0.8	C_7_H_6_O_2_	Benzoic acid	167.0357 [M+HCOO]^−^, 123.0450 [M+HCOO–CO_2_]^−^	RP	
10	5.50	[M−H]^−^	163.0401	163.0401	0.5	C_9_H_8_O_3_	*p*-Coumaric acid	163.0395 [M−H]^−^, 119.0499 [M−H–CO_2_]^−^	AS	P
11^a^	5.53	[M−H]^−^	367.1035	367.1036	0.3	C_17_H_20_O_9_	3-*O*-Feruloylquinic acid	193.0503 [M−H–quinic acid]^−^, 191.0566 [M−H–quinic acid–H_2_]^−^, 173.0488 [M−H–quinic acid–H_2_–H_2_O]^−^, 149.0612 [M−H–quinic acid–CO_2_]^−^, 134.0373 [M−H–quinic acid–CO_2_–CH_3_]^−^	AA	
12	5.72	[M−H]^−^	345.1555	345.1554	− 0.3	C_16_H_26_O_8_	Rehmapicroside	345.1166 [M−H]^−^, 183.1012 [M−H–Glc]^−^	RG	P
13	7.85	[M+HCOO]^−^	533.0937	533.0942	1	C_24_H_22_O_14_	Luteolin-7-*O*-(6-*O*-malonyl-β-d-glucoside)	533.1282 [M+HCOO]^−^, 487.1433 [M−H]^−^, 443.2878 [M−H–CO_2_]^−^	CM	
14^a^	8.90	[M−H]^−^	223.0976	223.0976	− 0.1	C_12_H_16_O_4_	Senkyunolide H	223.0244 [M−H]^−^, 207.9998 [M−CH_3_]^−^, 179.1067 [M−H–CO_2_]^−^	LC	P
15	9.13	[M−H]^−^	119.0502	119.0501	− 1	C_8_H_8_O	1-Phenyl-ethanone	119.0504 [M−H]−, 101.9300 [M−H–H_2_O]^−^	AS	
16	10.72	[M−H]^−^	387.1661	387.1662	0.4	C_18_H_28_O_9_	Tuberonic acid glucoside	387.1659 [M−H]^−^, 207.1028 [M−H–Glc–H_2_O]^−^, 163.1133 [M−H–Glc–H_2_O–CO_2_]^−^, 119.0341 [M−H–Glc–H_2_O–2CO_2_]^−^	CM	
17	11.74	[M−H]^−^	225.1132	225.1133	0.2	C_12_H_18_O_4_	Chuanxiongnolide R2	225.1117 [M−H]^−^, 207.0666 [M−H–H_2_O]^−^	LC	P
18	13.20	[M−H]^−^	479.1559	479.1555	− 0.7	C_23_H_28_O_11_	Paeoniflorin	327.1070 [M−H–C_7_H_6_O_2_]^−^, 121.0295 [M−H–C_16_H_22_O_9_]^−^	RP	P
19	13.21	[M+H]^+^	301.1070	301.1074	1.2	C_17_H_16_O_5_	(3*R*)-5,7-Dihydroxy-6-methyl-3-(4′-hydroxybenzyl)-chroman-4-one	283.1127 [M+H–H_2_O]^+^, 151.0746 [M+H–C_9_H_11_O_2_]^+^	PO	P
20	13.21	[M+H]^+^	463.16	463.1604	1.2	C_23_H_26_O_10_	Lactiflorin	342.0189 [M+K–Glc]^+^	RP	P
21	13.23	[M−H]^−^	569.1876	569.1873	− 0.5	C_26_H_34_O_14_	Torachrysone-8-*O*-β-d-gentiobioside	569.1258 [M−H]^−^, 479.1419 [M−H–C_3_H_6_O_3_]^−^, 449.1419 [M−H–C_4_H_8_O_4_]−	CO	
22	13.26	[M−H]^−^	193.0506	193.0507	0.5	C_10_H_10_O_4_	Ferulic acid	178.0272 [M−H–CH_3_]^−^, 134.0370 [M−H–CH_3_–CO_2_]^−^	LB, AS, LC	P
23	13.82	[M+H]^+^	595.166	595.1654	− 0.6	C_27_H_30_O_15_	Emodin-8-*O*-β-gentiobioside	577.1539 [M+H–H_2_O]^+^, 541.1365 [M+H–2H_2_O]^+^	CO	
24	17.01	[M+H]^+^	625.176	625.1758	− 0.8	C_28_H_32_O_16_	Narcissoside	625.1789 [M+H]^+^, 607.1656 [M+H–H_2_O]+, 589.1546 [M+H–2H_2_O]^+^, 571.1449 [M+H–3H_2_O]^+^	LB, BC	P
25^a^	17.03	[M+HCOO]^−^	623.1618	623.1612	− 0.9	C_27_H_30_O_14_	Kaempferitrin	623.1593 [M+HCOO]^−^, 533.1302 [M+HCOO–C_3_H_6_O_3_]−, 503.1192 [M+HCOO–C_4_H_8_O_4_]−, 413.0875 [M+HCOO–Arb–H_2_O]^−^, 383.0766 [M+HCOO–2C_4_H_8_O_4_]^−^	RP	P
26	19.04	[M−H]^−^	393.1191	393.1189	− 0.5	C_19_H_22_O_9_	6-Hydroxymusizin-8-*O*-β-d-glucoside	393.1171 [M−H]^−^, 273.0750 [M−H–C_4_H_8_O_4_]−, 231.0660 [M−H–Glc]^−^	CO	
27	19.64	[M−H]^−^	563.1406	563.1404	− 0.5	C_26_H_28_O_14_	Vicenin III	473.1076 [M−H–C_3_H_6_O_3_]^−^, 443.0987 [M−H–C_4_H_8_O_4_]^−^, 383.0774 [M−H–Glc–H_2_O]−, 365.0665 [M−H–Glc–2H_2_O]^−^, 353.0663 [M−H–C_4_H_8_O_4_–C_3_H_6_O_3_]^−^	CR	P
28	20.02	[M+H]^+^	209.117	209.1174	1	C_12_H_16_O_3_	Senkyunolide G	153.0543 [M+H–C_4_H_8_]^+^, 135.1182 [M+H–C_4_H_8_–H_2_O]^+^	LC	P
29	20.96	[M−H]^−^	417.1191	417.1189	− 0.6	C_21_H_22_O_9_	Liquiritin	417.1231 [M−H], 255.0658 [M−H–Glc]^−^, 135.0.87 [M−H–Glc–C_8_H_8_O]^−^, 119.0497 [M−H–Glc–C_7_H_4_O_3_]^−^	BC	P
30	21.80	[M+K]^+^	503.059	503.0567	− 3.9	C_21_H_20_O_12_	Isoquercitrin	503.1526 [M+H]^+^, 341.1019 [M+H–Glc]^+^	BC, LB	P
31	22.26	[M+HCOO]^−^	333.0616	333.0587	− 8.7	C_15_H_12_O_6_	Eriodictyol	333.1388 [M+HCOO]^−^, 289.1531 [M+HCOO–CO_2_]^−^	CR	P
32	22.33	[M−H]^−^	449.1089	449.1085	− 0.9	C_21_H_22_O_11_	Isookanin-7-*O*-β-diglucopyranoside	287.0550 [M−H–Glc]^−^, 151.0032 [M−H–C_8_H_8_O_2_]−, 135.0449 [M−H–C_7_H_7_O_4_]^−^	CM	P
33	24.67	[M−H]^−^	328.119	328.1189	− 0.6	C_18_H_19_NO_5_	*N*-trans-feruloyloctopamine	310.1071 [M−H–H_2_O]^−^	PO	
34^a^	24.87	[M+H]^+^	303.05	303.0501	0.7	C_15_H_10_O_7_	Quercetin	303.0512 [M+H]^+^, 257.0466 [M+H–H_2_O–CO]^+^, 229.0491 [M+H–H_2_O–2CO]^+^, 201.0544 [M+H–H_2_O–3CO]^+^, 153.0188 [M+H–C_8_H_6_O_3_]^+^, 137.0244 [M+H–H_2_O–C_8_H_6_O_3_]^+^	LB	P
35^a^	24.89	[M−H]^−^	609.1461	609.145	− 1.8	C_27_H_30_O_16_	Rutin	609.1426 [M−H]^−^, 301.0340 [M−H–Arb–Glc]^−^	BC	P
36	24.90	[M+H]^+^	611.161	611.1601	− 1	C_27_H_30_O_16_	Kaempferol 3,7-*O*-di-β-d-glucopyranside	611.1826 [M+H]^+^ , 303.0513 [M+H–Glc–Arb]^+^	RP	P
37	25.21	[M−H]^−^	461.0725	461.0721	− 1	C_21_H_18_O_12_	Luteolin-7-*O*-glucuronide	461.0720 [M−H]−, 285.0389 [M−H–C_9_H_8_O_6_]^−^	CM	P
38	26.49	[M−H]^−^	447.0933	447.0928	− 1.1	C_21_H_20_O_11_	Luteoloside	447.0921 [M−H]^−^, 285.0396 [M−H–Glc]^−^	CM	
39	26.52	[M+H]^+^	287.055	287.0552	0.8	C_15_H_10_O_6_	Alaternin	287.0553 [M+H]^+^, 241.0492 [M+H–H_2_O–CO]+, 151.0191 [M+H–C_8_H_6_O_2_–H_2_]^+^	CO	
40	26.79	[M−H]^−^	785.251	785.2502	− 1	C_35_H_46_O_20_	Echinacoside	785.2497 [M−H]^−^, 623.21289 [M−H–Glc]^−^	RG	
41	27.85	[M−H]^−^	893.2932	893.2925	− 0.8	C_38_H_54_O_24_	Torachrysone tetraglucoside	893.2927 [M−H]^−^, 245.0819 [M−H–4Glc]^−^	CO	
42	28.53	[M−H]^−^	187.0976	187.0974	− 0.9	C_9_H_16_O_4_	Anchoicacid	187.0973 [M−H]^−^, 169.0873 [M−H–H_2_O]−, 143.1072 [M−H–CO_2_]^−^, 125.0971 [M−H–CO_2_–H_2_O]^−^	AS	
43^a^	28.71	[M+Na]^+^	247.094	247.0938	− 0.9	C_12_H_16_O_4_	Senkyunolide I	247.0944 [M+H]^+^	AS	P
44	29.52	[M−H]^−^	627.1931	627.1924	− 1	C_28_H_36_O_16_	Cassialactone gentiobioside	627.1943 [M−H]^−^, 303.0867 [M−H–2Glc]^−^, 259.0975 [M−H–2Glc–CO_2_]^−^	CO	
45	30.42	[M− H]^−^	251.0561	251.0559	− 0.8	C_12_H_12_O_6_	3-(4-Hydroxy-3-methoxy-phenyl)-acrylic acid carbox-ymethyl ester	251.0554 [M−H]^−^, 233.0444 [M−H–H_2_O]^−^, 207.0666 [M−H–CO_2_]^−^	PO	
46	30.67	[M−H]^−^	799.2666	799.2659	− 0.9	C_36_H_48_O_20_	Jionoside A1	799.2662 [M−H]^−^, 623.2191 [M−H–C_10_H_10_O_3_]^−^, 605.2081 [M−H–C_10_H_10_O_3_–H_2_O]^−^	RG	P
47	30.75	[M−H]^−^	271.0612	271.0611	− 0.2	C_15_H_12_O_5_	Naringenin chalcone	271.0608 [M−H]^−^, 256.0374 [M−H–CH_3_]^−^, 228.0417 [M−H–CH_3_–CO_2_]^−^	CR	P
48	32.33	[M−H]^−^	515.1195	515.1189	− 1.2	C_25_H_24_O_12_	Isochlorogenic acid B	515.1168 [M−H]^−^, 353.0859 [M−H–C_9_H_6_O_3_]^−^, 335.0768 [M−H–C_9_H_6_O_3_–H_2_O]^−^, 191.0548 [M−H–2C_9_H_6_O_3_]^−^, 173.0447 [M−H–2C_9_H_6_O_3_–H_2_O]^−^	CM, BC	P
49^a^	32.54	[M+Cl]^−^	615.1486	615.1482	− 0.7	C_27_H_32_O_14_	Naringin	579.1714 [M−H]^−^, 271.0606 [M−H–Arb–Glc]^−^, 151.0037 [M−H–Arb–Glc–C_8_H_8_O]^−^	CR	P
50	33.19	[M+HCOO]^−^	477.1038	477.1011	− 5.9	C_21_H_20_O_10_	Isovitexin	477.1739 [M−H]^−^, 431.0987 [M−H–CO–H_2_O]^−^, 269.0457 [M−H–CO–H_2_O–Glc]^−^	CR	P
51	33.33	[M+H]^+^	433.113	433.1124	− 1.1	C_21_H_20_O_10_	Vitexin	271.0598 [M+H–Glc]^+^	CR	P
52	33.35	[M−H]^−^	431.0984	431.0979	− 1.1	C_21_H_20_O_10_	Puerarin	431.0954 [M−H]^−^, 269.0441 [M−H–Glc]^−^	BC	P
53	33.52	[M+H]^+^	221.081	221.081	0.6	C_12_H_12_O_4_	Senkyunolide E	206.1642 [M+H–CH_3_]^+^, 165.0197 [M+H–2CO]^+^	LC	P
54	34.13	[M+H]^+^	139.039	139.0383	− 5	C_7_H_6_O_3_	3,4-Dihydroxybenzaldehyde	121.0391 [M+H–H_2_O]^+^	AA	P
55	35.89	[M−H]^−^	623.1981	623.1975	− 1	C_29_H_36_O_15_	Acteoside	623.1955 [M−H]^−^, 461.1654 [M−H–C_9_H_6_O_3_]^−^	RG	P
56	36.04	[M−H]^−^	255.0663	255.0661	− 0.8	C_15_H_12_O_4_	(+)-Pinocembrin	135.0086 [M−H–C_7_H_4_O_3_]^−^, 119.0502 [M−H–C_8_H_8_O]^−^	CR	
57^a^	37.23	[M−H]^−^	301.0718	301.0715	− 0.7	C_16_H_14_O_6_	Hesperetin	301.0711 [M−H]^−^, 286.0475 [M−H–CH_3_]^−^, 257.0822 [M−H–CO_2_]^−^, 151.0038 [M−H–C_9_H_10_O_2_]^−^	CR	P
58	37.63	[M+H]^+^	303.086	303.0863	− 0.2	C_16_H_14_O_6_	5,7,2′,4′-Tetrahydroxyl homoisoflavanone	303.0866 [M+H]+, 177.0552 [M+H–C_9_H_8_O_5_–H_2_O]^+^	PO	
59	37.63	[M+H]^+^	611.197	611.196	− 1.7	C_28_H_34_O_15_	Hesperidin	465.1386 [M+H–Xyl]^+^, 303.0867 [M+H–Xyl–Glc]^+^	CR	P
60	37.79	[M−H]^−^	475.0882	475.0885	0.6	C_22_H_20_O_12_	Diosmetin 7-glucuronide	475.0883 [M−H]^−^, 299.0560 [M−H–C_6_H_8_O_6_]^−^, 284.0235 [M−H–C_6_H_8_O_6_–CH_3_]^−^	CM	
61	37.99	[M+H]^+^	463.123	463.1231	− 0.8	C_22_H_22_O_11_	Homoplantaginin	301.0713 [M+H–Glc]^+^, 286.0487 [M+H–Glc–CH_3_]^+^	CR	
62	38.00	[M+H]^+^	301.071	301.0709	0.9	C_16_H_12_O_6_	Hydroxygenkwanin	286.0498 [M+H–CH_3_]^+^	CR	
63	38.07	[M−H]^−^	299.0561	299.0563	0.5	C_16_H_12_O_6_	Diosmetin	299.0335 [M−H]^−^, 284.0335 [M−H–CH_3_]^−^, 256.0381 [M−H–CH_3_–CO]^−^, 227.0363 [M−H–CH_3_–2CO]^−^	CR	
64	38.41	[M+H]^+^	609.181	609.1809	− 0.8	C_28_H_32_O_15_	Physcion diglucoside	609.1849 [M+H]^+^, 463.1258 [M+H–Arb]^+^, 301.0716 [M+H–Arb–Glc]+	CO	
65^a^	38.42	[M+H]^+^	517.134	517.1335	− 1	C_25_H_24_O_12_	Isochlorogenic acid A	163.0395 [M+H–C_16_H_16_O_8_–H_2_O]+, 145.0293 [M+H–C_16_H_16_O_8_–2H_2_O]+	CM	
66	38.99	[M+H]^+^	255.065	255.0654	0.9	C_15_H_10_O_4_	Chrysophanol	209.0651 [M+H–H_2_O–CO]^+^	CO	
67	39.02	[M+H]^+^	435.129	435.1285	− 0.3	C_21_H_22_O_10_	Prunin	435.1298 [M+H]^+^, 273.0765 [M+H–Glc]^+^	CR	P
68	39.73	[M+H]^+^	557.186	557.1859	− 1	C_25_H_32_O_14_	Cassitoroside	557.1876 [M+H]^+^, 263.0931 [M+H–Glc–Arb]^+^	CO	
69^a^	39.80	[M−H]^−^	509.1664	509.1618	− 9.2	C_24_H_30_O_12_	Mudanpioside D	509.1921 [M−H]^−^, 463.2204 [M−H–H_2_O–CO]^−^	RP	
70	39.90	[M+H]^+^	314.139	314.1384	− 0.9	C_18_H_19_NO_4_	*N*-trans-Feruloyltyramine	177.0565 [M+H–C_8_H_8_O–NH_3_]^+^	LB	
71^a^	39.91	[M+HCOO]^−^	965.278	965.2787	0.7	C_39_H_52_O_25_	Cassiaside B2	919.2719 [M−H]^−^, 271.0615 [M−H–4Glc]^−^	CO	
72	39.97	[M−H]^−^	312.1241	312.1242	0.1	C_18_H_19_NO_4_	*N*-*trans*-feruloyltyramine	312.1253 [M−H]^−^, 297.0999 [M−H–CH_3_]^−^, 190.0507 [M−H–C_8_H_10_O]^−^, 178.0510 [M−H–CH_3_–C_8_H_8_O]^−^, 148.0532 [M−H–CH_3_–C_8_H_8_O–CH_2_O]^−^	PO	
73	41.48	[M+HCOO]^−^	623.1618	623.1617	0	C_27_H_30_O_14_	Chrysophanol-1-*O*-β-gentiobioside	253.0503 [M−H–2Glc]^−^	CO	
74	41.74	[M+HCOO]^−^	803.2251	803.2253	0.2	C_33_H_42_O_20_	Rubrofusarin triglucoside	803.4430 [M+HCOO]^−^, 757.4372 [M−H]−, 595.3829 [M−H–Glc]^−^	CO	
75	41.93	[M+H]^+^	597.181	597.1809	− 0.8	C_27_H_32_O_15_	Emodin-1-*O*-β-gentiobioside	417.1165 [M+H–Glc–H_2_O]^+^	CO	
76	41.96	[M−H]^−^	595.1668	595.1663	− 0.9	C_27_H_32_O_15_	Naringenin-6,8-di-C-glucoside	271.0596 [M−H–2Glc]^−^, 256.0372 [M−H–2Glc–CH_3_]^−^	CM	P
77	42.72	[M−H]^−^	637.2138	637.2132	− 0.9	C_30_H_38_O_15_	Jionoside D	637.2134 [M−H]^−^, 461.1664 [M−H–Arb–CH_2_O]^−^	RG	
78	43.02	[M+H]^+^	771.416	771.417	1.1	C_39_H_62_O_15_	Ophiopogonin R	771.2822 [M+H]^+^, 753.4137 [M+H–H_2_O]^+^, 591.3478 [M+H–H_2_O–Glc]^+^	OJ	
79	43.89	[M+H]^+^	1211.569	1211.5688	− 0.3	C_56_H_90_O_28_	Typaspidoside H	1211.5652 [M+H]^+^, 593.3691 [M+H–3Glc–Xyl]^+^, 575.3576 [M+H–3Glc–Xyl–H_2_O]^+^, 557.3466 [M+H–3Glc–Xyl–2H_2_O]^+^	PO	
80	43.90	[M+H]^+^	917.474	917.4744	0.4	C_45_H_72_O_19_	Polygoside A	917.4740 [M+H]^+^, 899.4642 [M+H–H_2_O]^+^, 881.4608 [M+H–2H_2_O]^+^, 737.4113 [M+H–H_2_O–Glc]^+^, 719.4014 [M+H–2H_2_O–Glc]^+^, 701.3944 [M+H–2H_2_O–Glc–H_2_O]^+^, 593.3722 [M+H–Glc–Glc]^+^, 429.3003 [M+H–3Glc–H_2_]^+^, 411.2900 [M+H–3Glc–H_2_–H_2_O], 393.2793 [M+H–3Glc–H_2_–2H_2_O]	PO	
81	43.93	[M−H]^−^	1079.528	1079.5263	− 1.6	C_51_H_84_O_24_	(25*S*)-26-*O*-(β-d-glucopyranosyl)-furost-5-en3β,22α,26-triol 3-*O*-β-d-glucopyranosyl-(1 → 2)-β-d-glucopyranosyl-(1 → 4)-β-d-gluco-pyranoside	1079.5283 [M−H]^−^, 1061.5114 [M−H–H_2_O]^−^, 737.4175 [M−H–2Glc]^−^, 575.3614 [M−H–3Glc]^−^, 557.3412 [M−H–3Glc–H_2_O]^−^, 395.2946 [M−H–4Glc–H_2_O]^−^, 413.3060 [M−H–4Glc–H_2_O]^−^	PO	
82	44.05	[M+H]^+^	567.171	567.1716	1.3	C_26_H_30_O_14_	Cassiaside B	417.1195 [M+H–Xyl–H_2_O]^+^, 273.0756 [M+H–Xyl–Glc]^+^	CO	P
83	44.54	[M+HCOO]^−^	331.0823	331.0804	− 5.9	C_16_H_14_O_5_	Isosakuranetin	316.0577 [M−H–CH_3_]^−^, 298.0483 [M−H–CH_3_–CO]^−^	CR	P
84	44.54	[M+H]^+^	595.202	595.2015	− 1	C_28_H_34_O_14_	Poncirin	449.1397 [M+H–Rha]^+^, 287.0904 [M+H–Rha–Glc]^+^	CR	P
85	44.55	[M+H]^+^	287.091	287.0919	1.7	C_16_H_14_O_5_	5,7,4′-Trihydroxyl homoisoflavanone	287.0923 [M+H]^+^, 153.0190 [M+H–C_8_H_8_O]^+^	PO	
86	44.86	[M+H]^+^	475.123	475.123	− 1	C_23_H_22_O_11_	Apigenin 7-*O*-acetylglucoside	475.1240 [M+H]^+^, 271.0595 [M+H–C_8_H_12_O_6_]^+^	CM	
87	44.92	[M+H]^+^	447.129	447.1273	− 2.8	C_22_H_22_O_10_	Acacetin-7-*O*-galactoside	285.0753 [M+H–Glc]^+^, 270.0526 [M+H–Glc–CH_3_]^+^, 242.0575 [M+H–Glc–CH_3_–CO]^+^, 153.0185 [M+H–Glc–C_9_H_8_O]^+^	CM	
88	44.97	[M−H]^−^	1195.5753	1195.5749	− 0.3	C_56_H_92_O_27_	Ophiopogonin F	1195.5726 [M−H]^−^, 739.4273 [M−H–2Glc–Xyl]^−^, 577.3731 [M−H–2Glc–Xyl–Glc]^−^, 559.3653 [M−H–2Glc–Xyl–Glc–H_2_O]^−^, 415.3208 [M−H–2Glc–Xyl–2Glc]^−^, 397.3106 [M−H–2Glc–Xyl–2Glc–H_2_O]^−^	OJ	
89	45.25	[M+Cl]−	219.0066	219.0066	0	C_8_H_8_O_5_	3-Methoxygallic acid	201.1643 [M+Cl–H_2_O]^−^, 175.1512 [M+Cl–H_2_O–CO_2_]^−^	RP	
90	45.27	[M+H]^+^	447.222	447.2211	− 3	C_21_H_34_O_10_	(Z)-(1*S*,5*R*)-β-pinen-10-yl-β-vicianoside	447.2211 [M+H]^+^, 285.0754 [M+H–C_6_H_10_O_5_]^+^	RP	
91	45.54	[M+H]^+^	507.15	507.149	− 1.4	C_24_H_26_O_12_	2-Gluco-chrysoobtusin	345.0960 [M+H–Glc]^+^, 330.0747 [M+H–Glc–CH_3_]^+^, 312.0632 [M+H–Glc–CH_3_–H_2_O]^+^	CO	P
92	45.55	[M+HCOO]^−^	551.1406	551.1402	− 0.8	C_24_H_26_O_12_	Obtusin glucoside	551.2850 [M+HCOO]^−^, 389.1246 [M+HCOO–Glc]^−^, 374.1044 [M+HCOO-Glc-CH_3_]−	CO	P
93	46.35	[M+H]^+^	189.091	189.0909	− 0.8	C_12_H_12_O_2_	(Z)-Butylidenephthalide	189.0913 [M+H]^+^, 171.0814 [M+H–H_2_O]^+^, 133.0287 [M+H–2CO]^+^	AS	P
94^a^	46.60	[M+H]^+^	191.107	191.1066	− 0.5	C_12_H_14_O_2_	Z-Ligustilide	191.1060 [M+H]^+^, 149.0606 [M+H–C_3_H_6_], 135.0442 [M+H–2CO]^+^	AS	P
95	46.61	[M−H]^−^	207.1027	207.1026	− 0.5	C_12_H_16_O_3_	Senkyunolide K	207.1735 [M−H]^−^, 189.0926 [M−H–H_2_O]^−^	LC	
96	48.08	[M−H]^−^	373.1293	373.129	− 0.8	C_20_H_22_O_7_	5,6,7,3′,4′-Pentamethoxyflavanone	373.1265 [M−H]^−^, 358.1054 [M−H–CH_3_]^−^, 343.0803 [M−H–2CH_3_]^−^	CR	P
97	48.15	[M+HCOO]^−^	373.0929	373.0928	− 0.2	C_18_H_16_O_6_	3-Hydroxy-5,7,8-trimethoxyflavone	373.0922 [M+HCOO]^−^, 358.0689 [M−H–CH_3_]^−^, 343.0458 [M−H–2CH_3_]^−^, 328.0233 [M−H–3CH_3_]^−^	CR	
98	48.57	[M+H]^+^	315.086	315.0867	1.2	C_17_H_14_O_6_	5,7,3′,4′-Tetramethoxyflavone	315.0872 [M+H]^+^, 300.0638 [M+H–CH_3_]^+^, 271.0604 [M+H–CO_2_]^+^	CR	
99^a^	48.64	[M−H]^−^	203.0714	203.0712	− 0.9	C_12_H_12_O_3_	Senkyunolide C	203.0708 [M−H]^−^, 173.0243 [M−H–CH_2_O]^−^, 160.0164 [M−H–H_2_O–CO]^−^	AS	
100	49.07	[M−H]^−^	373.0929	373.0926	− 0.9	C_19_H_18_O_8_	Casticin	373.0934 [M−H], 358.0689 [M−H–CH_3_], 343.0453 [M−H–2CH_3_]^−^, 328.0220 [M−H–3CH_3_]^−^, 315.0512 [M−H–2CO]^−^, 300.0274 [M−H–2CO–CH_3_]^−^	CM	
101	49.09	[M+H]^+^	331.118	331.1176	0.0	C_18_H_18_O_6_	(3*R*)-5,7-Dihydroxyl-6-methyl-8-methoxyl-3-(4′-hydroxylbenzyl)-chroman-4-one	331.1179 [M+H]^+^, 225.0761 [M+H–C_7_H_6_O]^+^, 210.0539 [M+H–C_7_H_6_O–CH_3_]^+^, 107.0494 [M+H–C_11_H_12_O_5_]^+^	PO	
102	49.10	[M+Na]^+^	427.136	427.1369	1.2	C_21_H_24_O_8_	5,6,7,8,3′,4′-Hexamethoxyflavanone	427.1381 [M+Na]^+^, 412.1150 [M+Na–CH_3_]^+^, 397.0894 [M+Na–2CH_3_], 381.0973 [M+Na–CO–H_2_O], 263.0543 [M+Na–C_10_H_12_O_2_], 248.0283 [M+Na–C_10_H_12_O_2_–CH_3_], 233.0046 [M+Na–C_10_H_12_O_2_–2CH_3_]	CR	
103	49.25	[M+H]^+^	359.113	359.1129	1.1	C_19_H_18_O_7_	5-Hydroxy-3,6,7,8-tetramethoxyflavone	359.1128 [M+H]^+^, 344.0891 [M+H–CH_3_]^+^, 326.0790 [M+H–CH_3_–H_2_O]^+^, 329.0661 [M+H–2CH_3_]^+^, 315.0871 [M+H–CO_2_]^+^	CR	P
104	49.31	[M+H]^+^	403.139	403.1372	-3.8	C_21_H_22_O_8_	5,6,7,3′,4′,5′-Hexamethoxyflavone	403.1355 [M+H]^+^, 388.1138 [M+H–CH_3_]^+^, 373.0892 [M+H–2CH_3_]^+^, 355.0811 [M+H–2CH_3_–H_2_O]^+^	CR	P
105	49.46	[M+H]^+^	343.118	343.1171	-1.6	C_19_H_18_O_6_	5,7,8,4′-Tetramethoxyflavone	343.1187 [M+H]^+^, 328.0945 [M+H–CH_3_]^+^, 325.0719 [M+H–H_2_O]^+^, 313.0719 [M+H–2CH_3_]^+^, 299.0910 [M+H–CO_2_]^+^	CR	P
106^a^	49.67	[M−H]^−^	313.1081	313.1081	-0.1	C_18_H_18_O_5_	Ophiopogonanoe B	207.0654 [M−H–C_7_H_6_O]^−^, 192.0422 [M−H–C_7_H_6_O–CH3]^−^, 179.0654 [M−H–C_7_H_6_O–CO]^−^	OJ	P
107	50.29	[M−H]^−^	283.0612	283.0611	− 0.3	C_16_H_12_O_5_	Acacetin	283.0565 [M−H]^−^, 268.0362 [M−H–CH_3_]^−^, 240.0415 [M−H–H_2_O–CO]^−^	CM	
108	50.30	[M−H]^−^	359.1136	359.1136	0	C_19_H_20_O_7_	3-Hydroxy-5,6,7,4′-tetramethoxyflavanone	359.1133 [M−H]^−^, 344.0903 [M−H–CH_3_]^−^, 329.0672 [M−H–2CH_3_]^−^	CR	P
109	50.33	[M+H]^+^	419.134	419.1337	0.1	C_21_H_22_O_9_	3-Hydroxy-5,6,7,8,3′,4′-hexamethoxyflavone	419.1339 [M+H]^+^, 404.1096 [M+H–CH_3_]^+^, 389.0872 [M+H–2CH_3_]^+^, 361.0912 [M+H–2CH_3_–CO]^+^, 343.0803 [M+H–2CH_3_–CO–H_2_O]^+^	CR	P
110	50.40	[M+HCOO]^−^	329.0667	329.066	-2.1	C_16_H_12_O_5_	Emodin-3-methyl ether	314.0248 [M+HCOO–CH_3_]^−^, 299.0196 [M+HCOO–2CH_3_]^−^, 271.0243 [M+HCOO–2CH_3_–CO]^−^, 243.029.3 [M+HCOO–2CH_3_–2CO]^−^	CO	P
111	50.53	[M+H]^+^	373.128	373.1273	− 2.4	C_20_H_20_O_7_	Isosinensetin	373.1265 [M+H]^+^, 358.1045 [M+H–CH_3_]^+^, 343.0800 [M+H–2CH_3_]^+^, 325.0715 [M+H–2CH_3_–H_2_O]^+^	CR	P
112	51.09	[M+HCOO]^−^	915.4595	915.4588	− 0.8	C_45_H_72_O_19_	Schidigerasaponin A1	869.4501 [M−H]^−^, 737.4100 [M−H–Xyl]^−^	PO	P
113	51.24	[M−H]^−^	925.5166	925.5155	− 1.2	C_48_H_78_O_17_	Saikosaponin C	925.5119 [M−H]^−^, 907.5071 [M−H–H_2_O]^−^	BC	P
114	53.82	[M−H]^−^	327.1238	327.1238	0	C_19_H_20_O_5_	Lophiopogonanone B	327.1230 [M−H]^−^, 205.0507 [M−H–C9H11O]^−^	OJ	P
115^a^	54.02	[M−H]^−^	853.4591	853.4596	0.6	C_44_H_70_O_16_	Ophiopogonin D	853.4574 [M−H]^−^, 721.4158 [M−H–Xyl]^−^, 575.3584 [M−H–Xyl–Glc]^−^	OJ	P
116	57.15	[M+H]^+^	205.086	205.0859	− 0.1	C_12_H_12_O_3_	Senkyunolide B	205.1614 [M+H]^+^, 149.0249 [M+H–2CO]^+^	AS	P
117	61.37	[M−H]^−^	255.233	255.2319	− 4.1	C_16_H_32_O_2_	Palmitic acid	255.2308 [M−H]^−^, 237.2230 [M−H–H_2_O]^−^	LC	

^a^Identified by comparison with reference standards

P: Prototypes were detected in cynomolgus monkey plasma; Glc: glucose; Rha: rhamnose; Xyl: xylose; *Rehmannia glutinosa* (RG, Shudihuang); *Angelica sinensis* (AS, Danggui); *Paeonia lactiflora* Pall (PL, Baishao); *Polygonatum odoratum* (PO, Yuzhu); *Ophiopogon japonicas* (OJ, Maidong); *Chrysanthemum morifolium* (CM, Juhua); *Ligusticum chuanxiong *(LC, Chuanxiong); *Anemone altaica* (AA, Jiujiechangpu); *Citrus reticulate* (CR, Chenpi); *Cassiae Semen *(CS, Juemingzi); *Lycii fructus* (LF, Gouqizi); *Bupleuri Radix *(BR, Chaihu)

### Identification of phenolic compounds in YGMM

Phenolic compounds are well known bioactive secondary metabolites in medicinal plants, which have been proved to possess antioxidative, antimicrobial, and anticarcinogenic activities. In their structures, usually there are more than one phenolic hydroxyl group attached to one or more benzene rings. Besides hydroxyl group, other common substituents in their structures are methoxy, carboxylic acid, glucose, rhamnose and xylose moieties. In the negative ion mode, neutral losses of 18 Da (H_2_O), 28 Da (CO), and 44 Da (CO_2_) were often detected in the MS/MS spectra [47]. In our study, 17 phenolic acids were detected in YGMM. Compound **2** gave the [M+H]^+^ ion at *m/z* 171.0290, which corresponded to the molecular formula of C_8_H_8_O_4_. In its MS/MS spectra, the fragment ions of *m/z* 153.0196 [M+H–H_2_O]^+^, 135.0087 [M+H–2H_2_O]^+^, and 109.0260 [M+H–CO_2_]^+^ were observed, which indicated the hydroxyl and carboxyl groups in the chemical structure, and thus compound **2** were identified as gallic acid. Compound **11** produced precursor ions at *m/z* 367.1036 [M−H]^−^ in the negative ion mode, and its molecular formula was supposed as C_17_H_20_O_9_. Fragments of this compound were found at *m/z* 193.0503, 191.0566, 173.0488, 149.0612, and 134.0373 in the high-collision-energy scan, which mainly resulted from the successively losses of one ferulic acid, one quinic acid, one H_2_O and one CO_2_. The detail fragment pathways of compound **11** were proposed in Additional file 1: Fig. S2 and was unambiguously identified as 3-*O*-feruloylquinic acid after confirmed with reference standard and those data reported in literature [48]. Based on the high resolution MS data and characteristic fragmentation patterns, other phenolic acids were identified respectively (Table 1).

### Identification of flavonoids in YGMM

In total of 25 flavonoids were identified in YGMM, which could be classified as flavones, flavonols, flavanones, and flavonols. RDA cleavages at B^1,3−^ position was the major fragmentation pathway observed in their MS/MS spectra. Other neutral losses such as CH_3_ (15 Da), CH_2_O (30 Da), H_2_O (18 Da), CO (28 Da), CO_2_ (44 Da), glucose (162 Da), rhamnose (146 Da), and xylose (132 Da) were also usually detected. The major fragmentation pathway of a representative compound was proposed and shown in Additional file 1: Fig. S3.

Compound **32** showed a protonated ion at *m/z* 449.1085, indicating the chemical formula of C_21_H_22_O_11_. Using the high-collision-energy scan mode, fragment ions at *m/z* 287.0550, 151.0032, and 135.0449 were observed. Ion at *m/z* 287.0550 was generated by the loss of one glucose (162 Da) group. Daughter ions at *m/z* 151.0032, and 135.0449 were the characterical fragments of flavones after RDA cleavage at B^1,3−^ position [4]. Thus, compound **32** were tentatively identified as isookanin-7-*O*-β-diglucopyranoside. The similar fragmentation behaviors could also been observed in the MS/MS spectra of compound **57**. Beside the characterical ion generated by the RDA cleavage at position B^1,3−^, other neutral loss such as H_2_O (18 Da) and CO_2_ (44 Da) were also used for identification, which resulted in the deduction of compound **57** as hesperetin, and it was firmly identified by comparison with the reference compound. Based on the similar fragmentation pathways, other flavonoids and their glycosides were identified, respectively.

Though sharing the same basic aglycone as the other flavonoids, the number and position of substituents such as hydroxyl and methyl groups on different rings (A, B, and C) of polymethoxylated flavones detected in YGMM resulted in their different molecular formulas and high resolution mass values. Compound **102** were taken as an example to characterize the typical MS/MS fragmentation behaviors of these polymethoxylated compounds. As shown in Additional file 1: Fig. S4. Compound **102** gave the [M+Na]^+^ ion at *m/z* 427.1369 and its molecular formula was established as C_21_H_24_O_8_. The consequently losses of methyl groups produced the fragment ions at *m/z* 412.1150 [M+Na–CH_3_]^+^, and 397.0894 [M+Na–2CH_3_]^+^, respectively. Fragment ion at 381.0973 [M+Na–CO–H_2_O]^+^ was corresponded with the losses of one CO and one H_2_O molecules. Beside the fragment ions mentioned above, fragment ion at *m/z* 263.0543 corresponded to [M+Na–C_10_H_12_O_2_]^+^, which was generated by the characteristic RDA cleavage at B^1,3−^ position. Fragment ions at *m/z* 248.0283 [M+Na–C_10_H_12_O_2_–CH_3_]^+^ and 233.0046 [M+Na–C_10_H_12_O_2_–2CH_3_]^+^ were also observed after expelling one and two methyl groups, respectively. Thus, compound **102** were tentatively identified as 5,6,7,8,3′,4′-hexamethoxyflavanone [49].

### Identification of phthalide compounds in YGMM

Phthalide compounds were bioactive components of LC and AS corresponding for their pharmacological properties, such as blood vessel protection, anti-thrombotic, anti-hypertensive, anti-atherosclerosis, anti-inflammatory, and anti-asthma effects. Neutral losses such as CH_3_ (15 Da), C_4_H_8_ (56 Da, side chain), H_2_O (18 Da), CO (28 Da), and CO_2_ (44 Da) were also usually detected. The major fragmentation pathways were proposed and shown in Additional file 1: Fig. S5. Based on these similar fragmentation patterns and reference standards, other phthalide compounds were identified respectively.

### Identification of monoterpenes in YGMM

In total of 5 monoterpenes from RG and RP were identified in YGMM. Neutral losses including H_2_O (18 Da), CH_2_O (30 Da), CO (28 Da), glycose (162 Da), and *p*-hydroxybenzoic acid (138 Da) of these compounds in the MS/MS spectra were helpful to confirm the existence of substituents such as hydroxyl, carbonyl, *p*-hydroxybenzoic acid, and glycosyl groups in the molecules. For example, in the MS/MS spectra of compound **18** (Additional file 1: Fig. S6), fragment ions at *m/z* 327.1070 (fragment ion), and 121.0295 (group ion) were detected, which corresponding to [M−H–C_7_H_5_O_2_(benzoic acid)–CH_2_O]^−^, and [C_7_H_5_O_2_]^−^, respectively. Thus, compound **18** were assigned as peoniflorin, and also was confirmed by comparison with the MS and MS/MS data with reference standard. Using the same method, compound **12**, **20**, **69**, and **90** were tentatively characterized as rehmapicroside, lactiflorin, mudanpioside D, and (Z)-(1*S*,5*R*)-β-pinen-10-yl-β-vicianoside, respectively.

### Identification of triterpenoid saponins in YGMM

Triterpenoid saponins were mainly classified into tetracyclic and pentacyclic types due to the ring numbers in their structures. In negative ion mode, triterpenoid saponins usually showed intense deprotonated ion, due to the existence of one or more hydroxyl groups in the structure, and were apt to expel the glucose (162 Da), rhamnose (146 Da) and xylose (132 Da) moieties [50]. The species and amount of glycosyl groups could be deduced from the fragment mass different. Major fragmentation pathways proposed for a typical triterpenoid saponin (compound **80**) were showed in Additional file 1: Fig.S7. Compound **80** showed a quasi-molecular ion [M+H]^+^ at *m/z* 917.4744, and its molecule formula was established as C_45_H_72_O_19_. In its MS/MS spectra, characterical ions at *m/z* 899.4642 and 881.4608 were generated by successive losses of one H_2_O (18 Da) and two H_2_O (36 Da) molecules, respectively. The other characterical ions at *m/z* 737.4113, 593.3722, and 429.3003 represented the successive losses of one, two, and three glucose (162 Da) units, respectively. Thus, compound **80** was tentatively characterized as polygoside A. Fragmentation patterns of the other triterpenoid saponins could be observed at their sugar or carbon side chains and thus identified.

### Identification of other compounds in YGMM

Using the same data analysis strategy, other compounds (see Table 1) were identified by detail analysis of their MS, and MS/MS data, and comparison of their fragmentation behaviors with those previously reported in the literature. For example, the Fragmentation patterns of the representative anthraquinone (emodin-3-methyl ether, compound **110**) were displayed in Additional file 1: Fig. S8.

### Identification of prototypes in cynomolgus monkey plasma

**Fig. 4:**
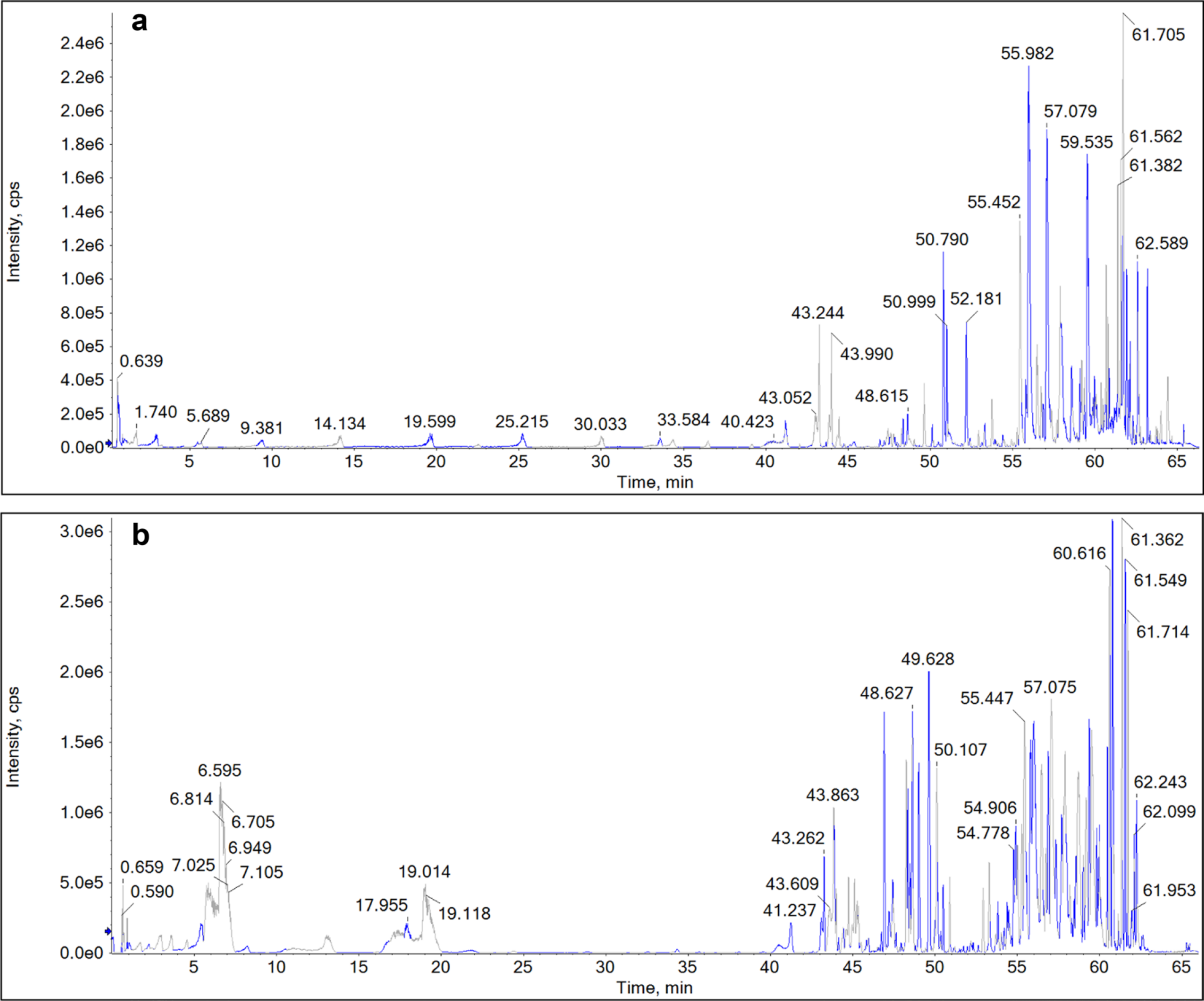
The BPC of drugged cynomolgus monkey plasma in the positive (**a**) and negative (**b**) ions modes

**Fig. 5:**
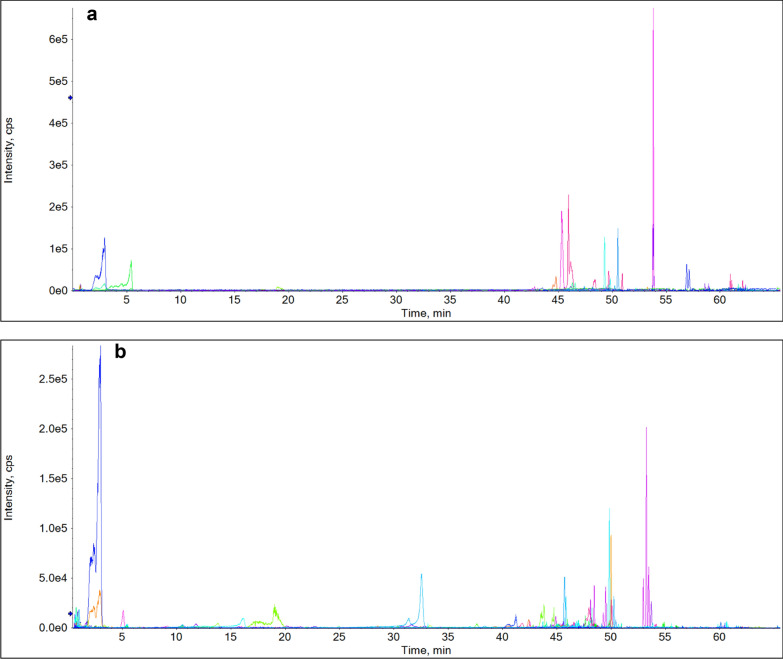
The extracted ion chromatograms (XICs) of prototypes in drugged cynomolgus monkey plasma in the positive (**a**) and negative (**b**) ions modes

Serum pharmacochemistry analysis was conducted to study the absorbed constituents in vivo, using cynomolgus monkey as animal model. The chemical profiles study of YGMM were helpful for the fast investigation of the absorbed prototypes in vivo. The blank and drugged blood samples were analyzed in both negative and positive modes by the established UPLC-Q-TOF-MS method (Fig. 4). The extracted ion chromatograms (XIC) of the absorbed prototypes of YGMM were shown in Fig. 5 and Table 1, respectively, and these compounds were confirmed by comparing their accurate mass measurements and retention times with the identified components in YGMM. As a result, a total of 61 prototypes were screened out.

## Discussion

Although the chemical composition of each single herbal medicine contained in YGMM have been studied and reported, the chemical profiles of YGMM have not been systematically characterized. To comprehensive characterize the constituents of such complex system is challenging, due to the various type structures such as flavonoids, phenolic acids, monoterpenes, and anthraquinones, etc. The established data analysis strategy in this study might be helpful to the fast characterization of chemical profiles in YGMM, and the self built database have been proved greatly helpful in the chemical identification because it could greatly reduces the scope of analysis and improves the accuracy of compound identification [4, 48]. As shown in Fig. 1, based on the reported chemical components from the 12 single herbal medicines, self built database could exclude many isomers from the other herbal medicines, and the candidate compounds were fast screened out by matching the experimental MS values with those theoretical value, and finally, the identified compounds were confirmed by comparisons of retention times, and MS/MS fragment patterns with standard compounds or those data reported.

Although TCM contains complex chemical components, only the ones that can be absorbed into the blood may produce effects [8]. Serum pharmacochemistry analysis is an effective tool to study absorbed compounds in blood, which may unveil the potential biomarkers in YGMM. Cynomolgus monkeys are a useful preclinical model [51] and have been applied in the pharmacokinetic study by our group [8]. The absorbed prototypes of YGMM in cynomolgus monkey plasma were studied by using the established data analysis strategy and the results might be used for its quality control research. The work of identifying metabolites from YGMM in vivo was still in progress by our lab.

## Conclusions

In this study, a rapid and effective data analysis strategy based on UPLC-Q-TOF-MS and self built components database was applied to the fast identification of the chemical constituents of YGMM in vitro and prototypes in vivo. 667 compounds were collected from the literatures of the 12 single herb medicines. With the help of the SCIEX OS software, 415 compounds were initially screened as candidate compounds in YGMM. Among which, a total of 117 compounds were identified or tentatively characterized, by detail analysis of their accurate mass measurements, characteristic neutral loss, MS/MS fragment pathways, and also by comparisons with standards and those data reported in the literatures, and these compounds were tentatively distributed to 12 medicinal materials, which included 17 phenolic acids, 25 flavonoids, 4 alkaloids, 10 phthalides, 5 monoterpenes, 8 triterpenoid saponins, 9 anthraquinones, and 39 other compounds. Serum pharmacochemistry analysis of the plasma of cynomolgus monkey resulted in the identification of 61 prototypes, which included 13 phenolic acids, 21 flavonoids, 8 phthalides, 3 monoterpenes, 4 triterpenoid saponins, and 12 other compounds. To the best of our knowledge, it was the first comprehensive study of the chemical profile of YGMM and its prototypes in vivo. Our study might provide a scientific basis for further research on pharmacological effects, action mechanism and quality control of YGMM.

## Supplementary Information

**Additional file 1: Table S1.** The chemical compounds of YGMM collected from literatures. **Table S2.** The screened candidate compounds in YGMM. **Figure S1.** The chemical structures of identified constituents in YGMM. **Figure S2.** The possible fragment pathway of 3-*O*-feruloylquinic acid. **Figure S3.** The possible fragment pathway of quercetin. **Figure S4.** The possible fragment pathway of 5,6,7,8,3′,4′-hexamethoxyflavanone. **Figure S5.** The possible fragment pathway of Z-Ligustilide. **Figure S6.** The possible fragment pathway of paeoniflorin. **Figure S7.** The possible fragment pathway of polygoside A. **Figure S8.** The possible fragment pathway of emodin-3-methyl ether.

## Data Availability

The research data generated from this study is included within the article and Additional file.

## Abbreviations

YGMM: Yiganmingmu oral liquid
TCM: Traditional Chinese medicine
ESI-MS: Electrospray ionization-mass spectrometer
LC–MS: Liquid chromatography paired with mass spectrometry
UPLC-Q-TOF-MS: Ultra performance liquid chromatography quadrupole time of flight mass spectrometer
BPC: Base peak chromatograms
RDA: Retro Diels Alder
XIC: Extracted ion chromatograms
