# Supplementary material for: Comprehensive characterization of the chemical constituents in Yiganmingmu oral liquid and the absorbed prototypes in cynomolgus monkey plasma after oral administration by UPLC-Q-TOF-MS based on the self built components database
Source: Chin Med. 2021 Apr 28;16:35. doi: 10.1186/s13020-021-00443-0 (PMC8080328; doi:10.1186/s13020-021-00443-0)
Supplement: Supplementary file 1 — Additional file 1: Table S1. The chemical compounds of YGMM collected from literatures. Table S2. The screened candidate compounds in YGMM. Figure S1. The chemical structures of identified constituents in YGMM. Figure S2. The possible fragment pathway of 3-O-feruloylquinic acid. Figure S3. The possible fragment pathway of quercetin. Figure S4. The possible fragment pathway of 5,6,7,8,3′,4′-hexamethoxyflavanone. Figure S5. The possible fragment pathway of Z-Ligustilide. Figure S6. The possible fragment pathway of paeoniflorin. Figure S7. The possible fragment pathway of polygoside A. Figure S8. The possible fragment pathway of emodin-3-methyl ether. [file 13020_2021_443_MOESM1_ESM.docx]

# Additional file 1

# Comprehensive characterization of the chemical constituents in Yiganmingmu oral liquid and the absorbed prototypes in cynomolgus monkey plasma after oral administration by UPLC-Q-TOF-MS based on the self built components database

Wei wei^1,2,3*^, Siwei Li^4*^, Linyou Cheng^3^, Erwei Hao, Xiaotao Hou^4^, Hua Zhou^3^, Jiagang Deng^2*^ and Xinsheng Yao^1*^

1. College of Pharmacy and International Cooperative Laboratory of Traditional Chinese Medicine Modernization and Innovative Drug Development of Chinese Ministry of Education, Jinan University, Guangzhou 510632, China

2. Guangxi Key Laboratory of Efficacy Study on Chinese Materia Medica, Guangxi University of Chinese Medicine, Nanning, Guangxi 530200, China

3. National and Region joint Engineering Center for Anticancer Drug Development, Guangxi Hebabiz Pharmaceutical Co. Ltd., Qinzhou 535000, China

4. Faculty of Pharmacy, Guangxi University of Chinese Medicine, Nanning 530200, China

Table S1. The chemical compounds of YGMM collected from literatures

| No. | Name | Formula | Source |
| --- | --- | --- | --- |
| 1 | Leonuride | C_15_H_24_O_9_ | RG |
| 2 | 5-Hydroxymethyl-2-furoic acid | C_6_H_6_O_4_ | RG |
| 3 | 5-Hydroxymethylfurfural | C_6_H_6_O_3_ | RG |
| 4 | 6-O-E-Feruloylajugol | C_25_H_32_O_12_ | RG |
| 5 | 8-Epiloganic acid | C_16_H_24_O_10_ | RG |
| 6 | Acteoside | C_29_H_36_O_15_ | RG |
| 7 | Adenosine | C_10_H_13_N_5_O_4_ | RG |
| 8 | Afrormosine | C_15_H_24_O_9_ | RG |
| 9 | Apigenin | C_15_H_10_O_5_ | RG |
| 10 | Aucubin | C_15_H_22_O_9_ | RG |
| 11 | Catalpol | C_15_H_22_O_10_ | RG |
| 12 | Citric acid | C_6_H_8_O_7_ | RG |
| 13 | Decaffeoyl-verbascoside | C_20_H_30_O_12_ | RG |
| 14 | Dihydrocatalpol | C_15_H_24_O_10_ | RG |
| 15 | Diosmetin | C_16_H_12_O_6_ | RG |
| 16 | Echinacoside | C_35_H_46_O_20_ | RG |
| 17 | Forsythiaside | C_29_H_36_O_15_ | RG |
| 18 | Forsythoside E | C_20_H_30_O_12_ | RG |
| 19 | Geniposide | C_17_H_24_O_10_ | RG |
| 20 | Gentisic acid | C_7_H_6_O_4_ | RG |
| 21 | Guanosine | C_10_H_13_N_5_O_5_ | RG |
| 22 | Isoacteoside | C_29_H_36_O_15_ | RG |
| 23 | Jioglutoside B1 | C_37_H_50_O_20_ | RG |
| 24 | Jionoside A1 | C_36_H_48_O_20_ | RG |
| 25 | Jionoside B1 | C_37_H_50_O_20_ | RG |
| 26 | Jionoside D | C_30_H_38_O_15_ | RG |
| 27 | Leucosceptoside A | C_30_H_38_O_15_ | RG |
| 28 | Luteolin | C_15_H_10_O_6_ | RG |
| 29 | Martynoside | C_31_H_40_O_15_ | RG |
| 30 | Melittoside | C_21_H_32_O_15_ | RG |
| 31 | Monomelittoside | C_15_H_22_O_10_ | RG |
| 32 | Oleanolic acid | C_30_H_48_O_3_ | RG |
| 33 | p-hydroxybenzoic acid | C_7_H_6_O_3_ | RG |
| 34 | Protocatechuic acid | C_7_H_6_O_4_ | RG |
| 35 | Purpureaside B | C_35_H_46_O_20_ | RG |
| 36 | Rehmaionoside A | C_19_H_34_O_8_ | RG |
| 37 | Rehmannioside A | C_21_H_32_O_15_ | RG |
| 38 | Rehmannioside C | C_21_H_34_O_14_ | RG |
| 39 | Rehmannioside D | C_27_H_42_O_20_ | RG |
| 40 | Rehmapicroside | C_16_H_26_O_8_ | RG |
| 41 | Salidroside | C_14_H_20_O_7_ | RG |
| 42 | Uridine | C_9_H_12_N_2_O_6_ | RG |
| 43 | Ursolic acid | C_30_H_48_O_3_ | RG |
| 44 | Verbascoside | C_29_H_36_O_15_ | RG |
| 45 | β-sitosterol | C_29_H_50_O | RG |
| 46 | Z-ligustilide | C_12_H_14_O_2_ | LC |
| 47 | Senkyunolide A | C_12_H_16_O_2_ | LC |
| 48 | Senkyunolide B | C_12_H_12_O_3_ | LC |
| 49 | Senkyunolide D | C_12_H_12_O_4_ | LC |
| 50 | Senkyunolide E | C_12_H_12_O_4_ | LC |
| 51 | Senkyunolide F | C_12_H_14_O_3_ | LC |
| 52 | Senkyunolide G | C_12_H_16_O_3_ | LC |
| 53 | Senkyunolide H | C_12_H_16_O_4_ | LC |
| 54 | Senkyunolide J | C_12_H_18_O_4_ | LC |
| 55 | Senkyunolide K | C_12_H_16_O_3_ | LC |
| 56 | Senkyunolide L | C_16_H_22_O_4_ | LC |
| 57 | Senkyunolide N | C_16_H_24_O_3_ | LC |
| 58 | Senkyunolide O | C_12_H_16_O_5_ | LC |
| 59 | Butylphthalide | C_12_H_14_O_2_ | LC |
| 60 | Z-butylidenephthalide | C_12_H_12_O_2_ | LC |
| 61 | 3-Butylidene-7-hydroxyphthalide | C_12_H_12_O_3_ | LC |
| 62 | 4-Hydroxy-3-butylphthalide | C_12_H_14_O_3_ | LC |
| 63 | 4，7-Dihydroxy-3-butylphthalide | C_12_H_14_O_4_ | LC |
| 64 | Cnidilide | C_12_H_18_O_2_ | LC |
| 65 | 7-Epoxyligustilide | C_12_H_14_O_3_ | LC |
| 66 | 3-Carboxyrthyl-phthalide | C_10_H_8_O_4_ | LC |
| 67 | Chuanxiongnolide Ｒ2 | C_12_H_18_O_4_ | LC |
| 68 | 7-Dihydroxy-3-butylphthalide | C_12_H_18_O_5_ | LC |
| 69 | Levistolide A | C_24_H_28_O_4_ | LC |
| 70 | Tokinolide B | C_24_H_28_O_4_ | LC |
| 71 | Ferulic acid | C_10_H_10_O_4_ | LC |
| 72 | Caffeic acid | C_9_H_8_O_4_ | LC |
| 73 | protocatechuic acid | C_7_H_6_O_4_ | LC |
| 74 | p-Hydroxybenzoicacid | C_7_H_6_O_3_ | LC |
| 75 | Vanillic acid | C_8_H_8_O_4_ | LC |
| 76 | Vanillin | C_8_H_8_O_3_ | LC |
| 77 | Sedanonic acid | C_12_H_18_O_3_ | LC |
| 78 | Gallic acid | C_7_H_6_O_5_ | LC |
| 79 | Chlorogenic acid | C_16_H_18_O_9_ | LC |
| 80 | Tetramethylpyrazine | C_8_H_12_N_2_ | LC |
| 81 | Adenine | C_5_H_5_N_5_ | LC |
| 82 | Scopoletin | C_10_H_8_O_4_ | LC |
| 83 | Daidzein | C_15_H_10_O_4_ | LC |
| 84 | Palmitic acid | C_16_H_32_O_2_ | LC |
| 85 | Isoleucine | C_6_H_13_NO_2_ | AS |
| 86 | Leucine | C_6_H_13_NO_2_ | AS |
| 87 | Chlorogenic acid | C_16_H_18_O_9_ | AS |
| 88 | Caffeic acid | C_9_H_8_O_4_ | AS |
| 89 | Phthalic acid | C_8_H_6_O_4_ | AS |
| 90 | Vanillic acid | C_8_H_8_O_4_ | AS |
| 91 | p-Coumaric acid | C_9_H_8_O_3_ | AS |
| 92 | Ferulic Acid | C_10_H_10_O_4_ | AS |
| 93 | Senkyunolide I | C_12_H_16_O_4_ | AS |
| 94 | Anchoicacid | C_9_H_16_O_4_ | AS |
| 95 | Senkyunolide H | C_12_H_16_O_4_ | AS |
| 96 | n-Butylidenephthalide | C_12_H_12_O_2_ | AS |
| 97 | Coniferyl ferulate | C_20_H_20_O_6_ | AS |
| 98 | 2-Valeryl-benzoic acid | C_12_H_14_O_3_ | AS |
| 99 | (Z)-6,7-epoxyligustilide | C_12_H_14_O_3_ | AS |
| 100 | Senkyunolide G | C_12_H_16_O_3_ | AS |
| 101 | Senkyunolide F | C_12_H_14_O_3_ | AS |
| 102 | (Z)-3-Butylidene-7-hydroxyphthalide | C_12_H_12_O_3_ | AS |
| 103 | 1-Phenyl-Ethanone | C_8_H_8_O | AS |
| 104 | Senkyunolide C | C_12_H_12_O_3_ | AS |
| 105 | Senkyunolide B | C_12_H_12_O_3_ | AS |
| 106 | Senkyunolide E | C_12_H_12_O_3_ | AS |
| 107 | Senkyunolide A | C_12_H_16_O_2_ | AS |
| 108 | Butylphthalide | C_12_H_14_O_2_ | AS |
| 109 | E-Ligustilide | C_12_H_14_O_2_ | AS |
| 110 | Ligustrazine | C_12_H_12_O_2_ | AS |
| 111 | Z-Ligustilide | C_12_H_14_O_2_ | AS |
| 112 | (E)-Butylidenephthalide | C_12_H_12_O_2_ | AS |
| 113 | (Z)-Butylidenephthalide | C_12_H_12_O_2_ | AS |
| 114 | Senkyunolide O | C_24_H_28_O_4_ | AS |
| 115 | Senkyunolide P | C_24_H_28_O_4_ | AS |
| 116 | Angelicide | C_24_H_28_O_4_ | AS |
| 117 | Levistilide A | C_24_H_28_O_4_ | AS |
| 118 | Riligustilide | C_24_H_28_O_4_ | AS |
| 119 | (4S)-perillic acid 6-O-α- L-arabinopyranosyl- (1' →6' ' )-β-D-glucopyranosyl | C_21_H_32_O_11_ | RP |
| 120 | 4-Hydroxybenzoic acid | C_7_H_6_O_3_ | RP |
| 121 | AlbiflorinR1 | C_23_H_28_O_11_ | RP |
| 122 | Aplopaeonoside | C_20_H_28_O_12_ | RP |
| 123 | Benzoylalbiflorin | C_30_H_32_O_12_ | RP |
| 124 | Benzoyloxypaeoniflorin | C_30_H_31_O_13_ | RP |
| 125 | Gallic acid | C_7_H_6_O_5_ | RP |
| 126 | Paeonol | C_9_H_10_O_3_ | RP |
| 127 | (1-O-β-D-glucopyransoyl-paeonisuf-frone) | C_16_H_24_O_9_ | RP |
| 128 | (Z)-(1S,5R)-β-pinen-10-yl-β-vicianoside | C_21_H_34_O_10_ | RP |
| 129 | 1,2,3,4,6-penta-O-galloyl-β-D- glucopyranoside | C_41_H_32_O_26_ | RP |
| 130 | 1,2,3,6-Tetragalloylglucose | C_34_H_28_O_22_ | RP |
| 131 | 1,2,3,6-Tetra-O-galloyl-β-D-glucose | C_34_H_27_O_22_ | RP |
| 132 | 1,3,6-Tri-O-galloyl-beta-D-glucose | C_27_H_24_O_18_ | RP |
| 133 | 1-O-β-D-glucopyranosyl-8-O-benzoylpaeonisuffrone | C_23_H_28_O_10_ | RP |
| 134 | 3', 6'-Di-O-gal-loylpaeoniflorin | C_37_H_36_O_19_ | RP |
| 135 | 3,4-Dihydroxybenzoic acid | C_7_H_6_O_4_ | RP |
| 136 | 30- norheder-agenin | C_29_H_44_O_4_ | RP |
| 137 | 3-Methoxygallic acid | C_8_H_8_O_5_ | RP |
| 138 | 3β-Hydroxy-20(29)-lupaene-28-oic acid | C_30_H_48_O_3_ | RP |
| 139 | 4"-Hydroxyl-albiflorin | C_23_H_28_O_12_ | RP |
| 140 | 4,9-Dihydroxy-8,10-dehydrothymol-1-O-β-D-glucoside | C_16_H_22_O_8_ | RP |
| 141 | 6'- O- β- D- glu-copyranosylalbiflorin | C_29_H_38_O_16_ | RP |
| 142 | 6' -O-galloylsucrose | C_19_H_26_O_15_ | RP |
| 143 | 6-Hydroxycoumarin | C_9_H_6_O_3_ | RP |
| 144 | 6-O-copyranosyl-lactinolide | C_16_H_26_O_9_ | RP |
| 145 | 6-O-galloylsucrose | C_13_H_16_O_10_ | RP |
| 146 | 8-Debenzoylpaeoniflorin | C_16_H_24_O_10_ | RP |
| 147 | Adenosine | C_10_H_13_N_5_O_4_ | RP |
| 148 | Ailexperphenoside A | C_21_H_30_O_11_ | RP |
| 149 | Albiflorin | C_23_H_28_O_11_ | RP |
| 150 | Astragaloside | C_21_H_20_O_11_ | RP |
| 151 | Benzoic acid | C_7_H_6_O_2_ | RP |
| 152 | Benzoylpaeoniflorin | C_30_H_32_O_13_ | RP |
| 153 | Benzoylpaeoniflorin sulfonate | C_30_H_32_O_14_S | RP |
| 154 | Betulonicacid | C_30_H_46_O_3_ | RP |
| 155 | Catechol | C_15_H_14_O_6_ | RP |
| 156 | Citric acid | C_6_H_8_O_7_ | RP |
| 157 | Daucosterol | C_35_H_60_O_6_ | RP |
| 158 | Diglucosyl Gallic Acid | C_19_H_26_O_15_ | RP |
| 159 | Dipropylphthalate | C_14_H_18_O_4_ | RP |
| 160 | Ellagic acid | C_14_H_6_O_8_ | RP |
| 161 | Emodin-8-O-β-D-glucoside | C_13_H_16_O_7_ | RP |
| 162 | Epigallocatechin gallate | C_22_H_18_O_11_ | RP |
| 163 | Ethyl gallate | C_9_H_10_O_5_ | RP |
| 164 | Ethyl palmitate | C_18_H_36_O_2_ | RP |
| 165 | Galloylpaeoniflorin | C_30_H_32_O_15_ | RP |
| 166 | Galloylpaeoniflorin | C_30_H_32_O_15_ | RP |
| 167 | Galloylpaeoniflorin | C_30_H_32_O_15_ | RP |
| 168 | Gentiopicroside | C_23_H_28_O_12_ | RP |
| 169 | Gentiopicroside | C_30_H_48_O_3_ | RP |
| 170 | Gentiopicroside | C_30_H_48_O_4_ | RP |
| 171 | Gentiopicroside | C_30_H_50_O_2_ | RP |
| 172 | Glucopyranose gallate | C_13_H_16_O_10_ | RP |
| 173 | Hederagenin | C_30_H_48_O_4_ | RP |
| 174 | Isomaltopaeoniflorin | C_29_H_38_O_16_ | RP |
| 175 | Kaempferitrin | C_27_H_30_O_14_ | RP |
| 176 | Kaempferol | C_15_H_10_O_6_ | RP |
| 177 | Kaempferol 3,7-O-di-β-D-glucopyranside | C_27_H_30_O_16_ | RP |
| 178 | KaeMpferol 3-O-D-glucopyranoside | C_21_H_18_O_12_ | RP |
| 179 | KaeMpferol-3-O-α-L-rhaMnoside | C_21_H_20_O_10_ | RP |
| 180 | Lactiflorin | C_23_H_26_O_10_ | RP |
| 181 | Leontoside A | C_14_H_18_O_9_ | RP |
| 182 | Methyl gallate | C_8_H_8_O_5_ | RP |
| 183 | Mudanpinoicacid A | C_30_H_46_O_3_ | RP |
| 184 | Mudanpioside A | C_31_H_34_O_13_ | RP |
| 185 | Mudanpioside B | C_31_H_34_O_14_ | RP |
| 186 | Mudanpioside D | C_24_H_30_O_12_ | RP |
| 187 | Mudanpioside H | C_30_H_32_O_14_ | RP |
| 188 | Mudanpioside E | C_24_H_29_O_13_ | RP |
| 189 | Mudanpioside F | C_16_H_24_O_8_ | RP |
| 190 | Mudanpioside G | C_16_H_24_O_8_ | RP |
| 191 | Mudanpioside H | C_30_H_32_O_14_ | RP |
| 192 | Mudanpioside I | C_23_H_28_O_11_ | RP |
| 193 | Naringenin | C_15_H_12_O_5_ | RP |
| 194 | Paeonidanin | C_24_H_30_O_11_ | RP |
| 195 | Paeonidanin E | C_46_H_54_O_21_ | RP |
| 196 | Paeoniflorin | C_23_H_28_O_11_ | RP |
| 197 | Paeoniflorin sulfite | C_23_H_28_O_13_S | RP |
| 198 | paeonilactone B | C_10_H_12_O_4_ | RP |
| 199 | Paeonilactone C | C_17_H_18_O_6_ | RP |
| 200 | Paeonin D | C_37_H_36_O_16_ | RP |
| 201 | Paeonisuffrone | C_10_H_14_O_4_ | RP |
| 202 | Paeonoside | C_15_H_20_O_8_ | RP |
| 203 | Pentagalloylglucose | C_41_H_32_O_26_ | RP |
| 204 | Protocatechualdehyde | C_7_H_6_O_3_ | RP |
| 205 | Pyrogallol | C_6_H_6_O_3_ | RP |
| 206 | Resveratrol | C_14_H_12_O_3_ | RP |
| 207 | Scabioside C | C_18_H_24_O_14_ | RP |
| 208 | Sucralose | C_12_H_22_O_11_ | RP |
| 209 | β-D-glucopyranosyl benzoate | C_21_H_20_O_10_ | RP |
| 210 | β-gentiobiosyl paeoniflorin | C_29_H_38_O_16_ | RP |
| 211 | Ophiofurospiside K | C_51_H_84_O_23_ | OJ |
| 212 | 5, 7-dihydroxy-6, 8-dimethyl-3-(2'- hydroxy, 3', 4'-methylenedioxybenzyl) chromone | C_19_H_16_O_7_ | OJ |
| 213 | 5,7, 2',4'-tetradihydroxy-8-methoyl-6-methyl-homoisoflavanone | C_18_H_18_O_7_ | OJ |
| 214 | 5,7,2’-trihydroxy-6,8-dimethyl-3-(3', 4'-methylenedioxybenzyl) chromone | C_19_H_16_O_7_ | OJ |
| 215 | 5,7,2'-trihydroxy-8-methyl-3-(3', 4'-methylenedioxybenzyl) chromone | C_17_H_16_O_5_ | OJ |
| 216 | 5,7,4'-trihydroxy ，3’-methoxyl-6,8-dimethyl-homisoflavanone. | C_19_H_20_O_6_ | OJ |
| 217 | 5,7-dihydroxy-6,8-dialdehyde-3-(4'- methoxybenzyl) chromone | C_18_H_14_O_7_ | OJ |
| 218 | 5,7-dihydroxy-6-dimethyl-3-(4'-hydroxybenzyl) chromone | C_17_H_14_O_5_ | OJ |
| 219 | 5,7-dihydroxy-8-formyl-3-(4'- methoxybenzyl) chromone | C_18_H_14_O_6_ | OJ |
| 220 | 7-dihydroxy-3-(4'-hydroxybenzyl) chromone-4-one | C_16_H_12_O_5_ | OJ |
| 221 | Dracaenoside F | C_39_H_62_O_13_ | OJ |
| 222 | Lophiopogonanone B | C_19_H_20_O_5_ | OJ |
| 223 | Methylophiopogonanone A | C_19_H_18_O_6_ | OJ |
| 224 | Methylophiopogonanone B | C_19_H_20_O_5_ | OJ |
| 225 | Methylophiopogonone A | C_19_H_16_O_6_ | OJ |
| 226 | Ophiofurospiside A | C_50_H_82_O_23_ | OJ |
| 227 | Ophiofurospiside C | C_56_H_92_O_28_ | OJ |
| 228 | Ophiofurospiside F | C_45_H_74_O_20_ | OJ |
| 229 | Ophiofurospiside L | C_50_H_82_O_23_ | OJ |
| 230 | Ophiopogonanoe A | C_18_H_16_O_6_ | OJ |
| 231 | Ophiopogonanoe B | C_18_H_18_O_5_ | OJ |
| 232 | Ophiopogonanone C | C_19_H_16_O_7_ | OJ |
| 233 | Ophiopogonanone E | C_19_H_20_O_7_ | OJ |
| 234 | Ophiopogonin B | C_39_H_62_O_12_ | OJ |
| 235 | Ophiopogonin C' | C_39_H_62_O_12_ | OJ |
| 236 | Ophiopogonin D | C_44_H_70_O_16_ | OJ |
| 237 | Ophiopogonin D' | C_44_H_70_O_16_ | OJ |
| 238 | Ophiopogonin F | C_56_H_92_O_27_ | OJ |
| 239 | Ophiopogonin G | C_56_H_92_O_27_ | OJ |
| 240 | Ophiopogonin G | C_56_H_92_O_27_ | OJ |
| 241 | Ophiopogonin H | C_56_H_92_O_29_ | OJ |
| 242 | Ophiopogonin I | C_50_H_82_O_23_ | OJ |
| 243 | Ophiopogonin J | C_56_H_92_O_27_ | OJ |
| 244 | Ophiopogonin N | C_56_H_92_O_28_ | OJ |
| 245 | Ophiopogonin O | C_50_H_80_O_24_ | OJ |
| 246 | Ophiopogonin P | C_41_H_64_O_14_ | OJ |
| 247 | Ophiopogonin Q | C_56_H_92_O_28_ | OJ |
| 248 | Ophiopogonin Ｒ | C_39_H_62_O_15_ | OJ |
| 249 | Ophiopogonin Ra | C_39_H_62_O_14_ | OJ |
| 250 | Ophiopojaponin C | C_44_H_70_O_18_ | OJ |
| 251 | Tb | C_39_H_62_O_13_ | OJ |
| 252 | Chlorogenic acid | C_16_H_18_O_9_ | CM |
| 253 | Apigenin-7-O-glucoside | C_21_H_20_O_10_ | CM |
| 254 | Luteolin-7-O-6''-malonyl-glucoside | C_24_H_22_O_14_ | CM |
| 255 | 4,5-Dicaffeoylquinic acid | C_25_H_24_O_12_ | CM |
| 256 | 3,5-Dicaffeoylquinic acid | C_25_H_24_O_12_ | CM |
| 257 | 3,4-Dicaffeoylquinic acid | C_25_H_24_O_13_ | CM |
| 258 | Acacetin-7-O-6''-malonyl-galactoside | C_25_H_24_O_13_ | CM |
| 259 | Caffeic acid | C_13_H_12_O_9_ | CM |
| 260 | Bellidifodin | C_14_H_10_O_6_ | CM |
| 261 | Apigenin | C_15_H_10_O_5_ | CM |
| 262 | Luteolin | C_15_H_10_O_6_ | CM |
| 263 | Acacetin | C_16_H_12_O_5_ | CM |
| 264 | Diosmetin | C_16_H_12_O_6_ | CM |
| 265 | Neochlorogenicacid | C_16_H_18_O_10_ | CM |
| 266 | Chlorogenic acid | C_16_H_18_O_11_ | CM |
| 267 | Cryptochlorogenic acid | C_16_H_18_O_12_ | CM |
| 268 | 1-Caffeoylquinic acid | C_16_H_18_O_9_ | CM |
| 269 | Neochlorogenic acid | C_16_H_18_O_9_ | CM |
| 270 | Chlorogenic acid | C_16_H_18_O_9_ | CM |
| 271 | Eupatorin | C_18_H_16_O_7_ | CM |
| 272 | Tuberonic acid glucoside | C_18_H_28_O_9_ | CM |
| 273 | Norswertianolin | C_19_H_18_O_11_ | CM |
| 274 | Casticin | C_19_H_18_O_8_ | CM |
| 275 | Chlorogenic acid butyl ester | C_20_H_26_O_9_ | CM |
| 276 | Apigenin-7-O-glucuronide | C_21_H_18_O_11_ | CM |
| 277 | Luteolin-7-O-glucuronide | C_21_H_18_O_12_ | CM |
| 278 | Apigenin-7-O-β-D-glucopyranoside | C_21_H_20_O_10_ | CM |
| 279 | Apigenin-4'-O-glucopyranoside | C_21_H_20_O_10_ | CM |
| 280 | Luteoloside | C_21_H_20_O_11_ | CM |
| 281 | Luteolin-7-O-glucoside | C_21_H_20_O_11_ | CM |
| 282 | Quercetin-7-O-galactoside | C_21_H_20_O_12_ | CM |
| 283 | Quercetin-3-O-glucoside | C_21_H_20_O_12_ | CM |
| 284 | Isookanin-7-O-β-diglucopyranoside | C_21_H_22_O_11_ | CM |
| 285 | Acacetin-7-O-glucuronide | C_22_H_20_O_11_ | CM |
| 286 | Diosmetin 7-glucuronide | C_22_H_20_O_12_ | CM |
| 287 | Acacetin-7-Glucoside | C_22_H_22_O_10_ | CM |
| 288 | Acacetin-7-O-galactoside | C_22_H_22_O_10_ | CM |
| 289 | Apigenin 7-O-acetylglucoside isomer | C_23_H_22_O_11_ | CM |
| 290 | Apigenin-7-O-6''-acetyl-glucoside | C_23_H_22_O_11_ | CM |
| 291 | Kaempferol-3-O-acetyl-glucoside | C_23_H_22_O_12_ | CM |
| 292 | Apigenin-7-O-6''-malonyl-glucoside | C_24_H_22_O_13_ | CM |
| 293 | Isochlorogenic acid C | C_25_H_24_O_12_ | CM |
| 294 | Isochlorogenic acid A | C_25_H_24_O_12_ | CM |
| 295 | Isochlorogenic acid B | C_25_H_24_O_12_ | CM |
| 296 | 1,3-Dicaffeoylquinic acid | C_25_H_24_O_12_ | CM |
| 297 | Dicaffeoylquinic acid isomer | C_25_H_24_O_12_ | CM |
| 298 | 3,4-Di-caffeoyl-quinic acid | C_25_H_24_O_12_ | CM |
| 299 | Acacetin-7-O-6″-malonylgactoside | C_25_H_24_O_13_ | CM |
| 300 | Diosmetin-7-O-6''-malonyl-glucoside | C_25_H_24_O_14_ | CM |
| 301 | Tasumatrol B | C_26_H_38_O_11_ | CM |
| 302 | Apigenin-7-O-rutinoside | C_27_H_30_O_14_ | CM |
| 303 | Apigenin-7-O-neohesperidoside | C_27_H_30_O_14_ | CM |
| 304 | Luteolin 7-O-rutinoside | C_27_H_30_O_15_ | CM |
| 305 | 6,8-C,C-diglucosylapigenin isomer | C_27_H_30_O_15_ | CM |
| 306 | Luteolin-7-O-rutinoside | C_27_H_30_O_15_ | CM |
| 307 | Naringenin-6,8-di-C-glucoside | C_27_H_32_O_15_ | CM |
| 308 | Buddleoside | C_28_H_32_O_14_ | CM |
| 309 | Diosmetin 7-O-rutinoside | C_28_H_32_O_15_ | CM |
| 310 | Ombuin-3β-rutinoside | C_29_H_34_O_16_ | CM |
| 311 | Malonic acid | C_7_H_8_O_7_ | CM |
| 312 | 6-Gingerol | C_17_H_26_O_4_ | BC |
| 313 | Baicalin | C_21_H_18_O_11_ | BC |
| 314 | Hydroxy-saikosaponin A | C_42_H_70_O_14_ | BC |
| 315 | Hydroxy-saikosaponin C | C_48_H_80_O_18_ | BC |
| 316 | Hydroxy-saikosaponin D | C_42_H_70_O_14_ | BC |
| 317 | Malonyl-saikosaponin A | C_45_H_70_O_16_ | BC |
| 318 | Malonyl-saikosaponin C | C_51_H_80_O_20_ | BC |
| 319 | Malonyl-saikosaponin D | C_45_H_70_O_16_ | BC |
| 320 | Saikochromoside A | C_17_H_20_O_10_ | BC |
| 321 | (-)-(5S,6S)-5,6-Dihydro-3,8,10-trihydroxy-5-(4-hydroxy-3-methoxyphenyl)-6-hydroxymethyl-2,4-dimethoxy7H-benzo[c]xanthen-7-one | C_27_H_24_O_10_ | BC |
| 322 | 2"-O-acetylsaikosaponin A | C_44_H_71_O_14_ | BC |
| 323 | 23-O-acetylsaikosaponin-A | C_44_H_70_O_14_ | BC |
| 324 | 2''-O-acetylsaikosaponin A | C_44_H_70_O_14_ | BC |
| 325 | 3"-O-acetylsaikosaponin A | C_44_H_70_O_14_ | BC |
| 326 | 3"-O-Acetylsaikosaponin D | C_44_H_70_O_14_ | BC |
| 327 | 3,7-Di-O-methylquercetin | C_17_H_14_O_7_ | BC |
| 328 | 4"-O-Acetylsaikosaponin A | C_44_H_70_O_15_ | BC |
| 329 | 4"-O-Acetylsaikosaponin D | C_44_H_70_O_15_ | BC |
| 330 | 5-hydroxy-7-acetoxylsaccharidesavone | C_17_H_12_O_5_ | BC |
| 331 | 6"-O-acetylsaikosaponin A | C_44_H_70_O_14_ | BC |
| 332 | 6''-O-acetylsaikosaponin A | C_44_H_70_O_14_ | BC |
| 333 | 6''-O-acetylsaikosaponin D | C_44_H_70_O_16_ | BC |
| 334 | Acetyl-prosaikogenin D | C_36_H_58_O_8_ | BC |
| 335 | Ammonium Glycyrrhizinate | C_42_H_62_O_16_._3_H_2_O.H_3_N | BC |
| 336 | Chikusaikoside I | C_47_H_76_O_17_ | BC |
| 337 | Chlorogenic acid | C_16_H_18_O_9_ | BC |
| 338 | Eugenin | C_10_H_12_O_2_ | BC |
| 339 | Gallic acid | C_7_H_6_O_5_ | BC |
| 340 | Ginsenoside Rb1 | C_54_H_92_O_23_ | BC |
| 341 | Ginsenoside Re | C_48_H_82_O_18_ | BC |
| 342 | Isochlorogenic acid A | C_25_H_24_O_12_ | BC |
| 343 | Isochlorogenic acid B | C_25_H_24_O_13_ | BC |
| 344 | Isoquercitrin | C_21_H_20_O_12_ | BC |
| 345 | Liquiritin | C_21_H_22_O_9_ | BC |
| 346 | Luteolin | C_15_H_10_O_6_ | BC |
| 347 | Malonyl-saikosaponin B2 | C_51_H_80_O_21_ | BC |
| 348 | Malonyl-saikosaponin E | C_45_H_70_O_15_ | BC |
| 349 | malonyl-saikosaponin F | C_51_H_82_O_20_ | BC |
| 350 | Narcissoside | C_28_H_32_O_16_ | BC |
| 351 | Prosaikogenin A | C_36_H_58_O_8_ | BC |
| 352 | Prosaikogenin D | C_36_H_58_O_8_ | BC |
| 353 | Prosaikogenin F | C_36_H_58_O_8_ | BC |
| 354 | Prosaikogenin G | C_36_H_58_O_8_ | BC |
| 355 | Puerarin | C_21_H_20_O_10_ | BC |
| 356 | Rotundifolioside I | C_47_H_76_O_16_ | BC |
| 357 | Rutin | C_27_H_30_O_16_ | BC |
| 358 | Saikochrome A | C_43_H_72_O_14_ | BC |
| 359 | Saikosaponin A | C_42_H_68_O_13_ | BC |
| 360 | Saikosaponin B1 | C_42_H_68_O_13_ | BC |
| 361 | Saikosaponin B2 | C_42_H_68_O_13_ | BC |
| 362 | Saikosaponin B3 | C_43_H_72_O_14_ | BC |
| 363 | Saikosaponin B4 | C_43_H_72_O_14_ | BC |
| 364 | Saikosaponin C | C_48_H_78_O_17_ | BC |
| 365 | Saikosaponin D | C_42_H_68_O_13_ | BC |
| 366 | Saikosaponin E | C_42_H_68_O_12_ | BC |
| 367 | Saikosaponin F | C_48_H_80_O_17_ | BC |
| 368 | Saikosaponin F | C_48_H_80_O_17_ | BC |
| 369 | Saikosaponin G | C_42_H_68_O_13_ | BC |
| 370 | Saikosaponin I | C_42_H_68_O_14_ | BC |
| 371 | Saikosaponin N | C_48_H_78_O_18_ | BC |
| 372 | Saikosaponin S | C_48_H_77_O_18_ | BC |
| 373 | Saikosaponin X | C_42_H_66_O_15_ | BC |
| 374 | Salcolin B | C_27_H_26_O_11_ | BC |
| 375 | Tartronoyl-saikosaponin D | C_45_H_70_O_16_ | BC |
| 376 | Tricin 4"-O-(threo-β-guaiacylglyceryl) Ether | C_29_H_28_O_12_ | BC |
| 377 | Tricin 4"-O-(threo-βguaiacylglyceryl) Ether⁃7"⁃O⁃β⁃D⁃glucopyranose | C_33_H_36_O_16_ | BC |
| 378 | Vittariflavone | C_24_H_26_O_12_ | BC |
| 379 | Rhamnetin | C_16_H_12_O_7_ | BC |
| 380 | 5,7,2',4'-tetrahydroxy-6-methoxy-8-methyl homoisoflavanone | C_18_H_18_O_7_ | PO |
| 381 | trans-ferulamide | C_10_H_11_NO_3_ | PO |
| 382 | 3-(4-Hydroxy-3-methoxy-phenyl)-acrylic acid carbox-ymethyl ester | C_12_H_12_O_6_ | PO |
| 383 | emodin | C_15_H_10_O_5_ | PO |
| 384 | physcion | C_16_H_12_O_5_ | PO |
| 385 | 5,7,4'-trihydroxyl homoisoflavanone | C_16_H_14_O_5_ | PO |
| 386 | (3R)-5,7-dihydroxyl-3-(2',4'-dihydroxylbenzyl)-chroman-4-one | C_16_H_14_O_6_ | PO |
| 387 | 5,7,2',4'-tetrahydroxyl homoisoflavanone | C_16_H_14_O_6_ | PO |
| 388 | (3R)-5,7-dihydroxy-6-me-thyl-3-(4′-hydroxybenzyl)-chroman-4-one | C_17_H_16_O_5_ | PO |
| 389 | (3R)-5,7-dihydroxyl-6-methyl-3-(4'-hydroxylbenzyl)-chroman-4-one | C_17_H_16_O_5_ | PO |
| 390 | 5,7,4'-trihydroxy-6-methyl homoisoflavanone | C_17_H_16_O_5_ | PO |
| 391 | 5,7,4'-trihydroxy-8-methyl homoisoflavanone | C_17_H_16_O_5_ | PO |
| 392 | 5,7,2',4'-tetrahydroxy-6-methyl-homoisoflavanone | C_17_H_16_O_6_ | PO |
| 393 | 5,7,2',4'-tetrahydroxy-8-methyl homoisoflavanone | C_17_H_16_O_6_ | PO |
| 394 | 5,7,2'-trihydroxy-4'-methoxyl homoisoflavanone | C_17_H_16_O_6_ | PO |
| 395 | 5,7,2',4'-tetrahydroxy-8-methoxyl homoisoflavanone | C_17_H_16_O_7_ | PO |
| 396 | N-trans-p-coumaroyltyramine | C_17_H_17_NO_3_ | PO |
| 397 | N-cis-p-coumaroyltyramine | C_17_H_17_NO_3_ | PO |
| 398 | N-trans-p-coumaroyloctopamine | C_17_H_17_NO_4_ | PO |
| 399 | N-trans-p-coumaroyloctopamine | C_17_H_17_NO_4_ | PO |
| 400 | N-cis-p-coumaroyloctopamine | C_17_H_17_NO_4_ | PO |
| 401 | (E)-3-(4-Hydroxy-3-methoxybenzylidene)-4-(4-hydroxyphenyl)pyrrolidin-2-one | C_18_H_17_NO_4_ | PO |
| 402 | (3R)-5,7-dihydroxy-6,8-dimethyl-3-(4′-hydroxybenzyl)-chroman-4-one | C_18_H_18_O_5_ | PO |
| 403 | (3R)-5,7-dihydroxyl-6,8-dimethyl-3-(4'-hydroxylbenzyl)-chroman-4-one | C_18_H_18_O_5_ | PO |
| 404 | 5,7,4'-trihydroxyl-6,8-dimethyl homoisoflavanone | C_18_H_18_O_5_ | PO |
| 405 | (3R)-5,7-dihydroxyl-6-methyl-8-methoxyl-3-(4'-hydroxylbenzyl)-chroman-4-one | C_18_H_18_O_6_ | PO |
| 406 | 5,7,2'-trihydroxy-8-methyl-4'-methoxyl homoisoflavanone | C_18_H_18_O_6_ | PO |
| 407 | 5,7,4'-trihydroxy-6-methyl-8-methoxyl homoisoflavanone | C_18_H_18_O_6_ | PO |
| 408 | 5,7,2'-trihydroxy-8,4'-dimethoxy homoisoflavanone | C_18_H_18_O_7_ | PO |
| 409 | N-cis-feruloyltyramine | C_18_H_19_NO_4_ | PO |
| 410 | N-trans-feruloyltyramine | C_18_H_19_NO_4_ | PO |
| 411 | N-cis-feruloyloctopamine | C_18_H_19_NO_5_ | PO |
| 412 | N-trans-feruloyloctopamine | C_18_H_19_NO_5_ | PO |
| 413 | 5,7-dihydroxyl-6-methyl-8,4'-dimethoxyl homoisoflavanone | C_19_H_20_O_6_ | PO |
| 414 | 5,7,2'-trihydroxy-6-methyl-8,4'-dimethoxyl homoisoflavanone | C_19_H_20_O_7_ | PO |
| 415 | polygodoquinone A | C_28_H_20_O_8_ | PO |
| 416 | polygonatumoside G | C_33_H_54_O_10_ | PO |
| 417 | Polygoside A | C_45_H_72_O_19_ | PO |
| 418 | Polygoside B | C_45_H_72_O_19_ | PO |
| 419 | officinalisnin II | C_45_H_76_O_19_ | PO |
| 420 | Polygodoraside B | C_50_H_80_O_24_ | PO |
| 421 | (25S)-26-O-(β-D-glucopyranosyl)-furost-5-en3β,22α,26-triol 3-O-β-D-glucopyranosyl-(1 → 2)-β-D-glucopyranosyl-(1 → 4)-β-D-gluco-pyranoside | C_51_H_84_O_24_ | PO |
| 422 | typaspidoside L | C_56_H_90_O_27_ | PO |
| 423 | Polygodoraside E | C_56_H_90_O_28_ | PO |
| 424 | typaspidoside H | C_56_H_90_O_28_ | PO |
| 425 | Polygodoraside A | C_56_H_90_O_29_ | PO |
| 426 | Polygodoraside D | C_56_H_90_O_29_ | PO |
| 427 | Polygodoraside F | C_56_H_90_O_29_ | PO |
| 428 | Polygodoraside C | C_56_H_90_O_30_ | PO |
| 429 | timosaponin H1 | C_56_H_92_O_28_ | PO |
| 430 | polygonatumoside F | C_56_H_92_O_29_ | PO |
| 431 | Polygodoraside H | C_57_H_94_O_29_ | PO |
| 432 | Polygodoraside G | C_57_H_94_O_30_ | PO |
| 433 | Trans-p-coumaramide | C_9_H_8_NO_2_ | PO |
| 434 | Emodin-6-glucoside | C_21_H_19_O_10_ | CO |
| 435 | Isorubrofusarin-6-O-β-gentiobioside | C_27_H_32_O_15_ | CO |
| 436 | Rubrofusarin-6-O-β-glucoside | C_21_H_22_O_10_ | CO |
| 437 | 1-Desmethyl- aurantio-obtusin-2- O-β-D- glucopyranoside | C_22_H_22_O_12_ | CO |
| 438 | 1-Desmethylobtusin | C_17_H_14_O_7_ | CO |
| 439 | 2-Gluco- chrysoobtusin | C_24_H_26_O_12_ | CO |
| 440 | 2-Gluco-chryso-obtusin | C_25_H_28_O_12_ | CO |
| 441 | 6-Hydroxymusizin-8-O-β-D-glucoside | C_19_H_22_O_9_ | CO |
| 442 | Alaternin | C_15_H_10_O_6_ | CO |
| 443 | Alaternin-2-O-β-D-glucoside | C_21_H_20_O_11_ | CO |
| 444 | Aloe-emodin | C_15_H_10_O_5_ | CO |
| 445 | Aloe-emodin-8-O-β-D-glucoside | C_21_H_20_O_10_ | CO |
| 446 | Aurantioobtusin | C_17_H_14_O_7_ | CO |
| 447 | Aurantio-obtusin-6-O-β-D-glucoside | C_23_H_24_O_12_ | CO |
| 448 | Cassia-lactone gentiobioside | C_28_H_36_O_16_ | CO |
| 449 | Cassiaside | C_20_H_20_O_10_ | CO |
| 450 | Cassiaside B | C_26_H_30_O_14_ | CO |
| 451 | Cassiaside B2 | C_39_H_52_O_25_ | CO |
| 452 | Cassiaside C | C_27_H_32_O_15_ | CO |
| 453 | Cassiaside C2 | C_39_H_52_O_25_ | CO |
| 454 | Cassitoroside | C_25_H_32_O_14_ | CO |
| 455 | Chrysophanol | C_15_H_10_O_4_ | CO |
| 456 | Chrysophanol 1-glucoside | C_21_H_20_O_9_ | CO |
| 457 | Chrysophanol-1-glucoside | C_33_H_40_O_19_ | CO |
| 458 | Chrysophanol-1-glucoside | C_39_H_50_O_24_ | CO |
| 459 | Chrysophanol-1-O-β-gentiobioside | C_27_H_30_O_14_ | CO |
| 460 | Citreorosein | C_15_H_10_O_6_ | CO |
| 461 | Emodin | C_15_H_10_O_5_ | CO |
| 462 | Emodin-1-O-β-gentiobioside | C_27_H_32_O_15_ | CO |
| 463 | Emodin-3-methyl ether | C_16_H_12_O_5_ | CO |
| 464 | Emodin-6-O-β-gentiobioside | C_27_H_30_O_15_ | CO |
| 465 | Gluco-aurantio-obtusin | C_23_H_24_O_12_ | CO |
| 466 | Isorubrofusarin 6-O-beta-gentiobioside | C_27_H_32_O_15_ | CO |
| 467 | Nor-rubrofusarin | C_26_H_30_O_15_ | CO |
| 468 | Nor-rubrofusarin-6-O-β-D-(6'-O-acetyl ) glucopyranoside | C_22_H_22_O_11_ | CO |
| 469 | Nor-rubrofusarin-6-O-β-D-glucophyranoside | C_20_H_20_O_10_ | CO |
| 470 | Obtusifolin | C_16_H_12_O_5_ | CO |
| 471 | Obtusin | C_24_H_26_O_12_ | CO |
| 472 | Physcion 8-glucoside | C_28_H_32_O_15_ | CO |
| 473 | Physcion-8-O-β-glucoside | C_22_H_22_O_10_ | CO |
| 474 | Pureonebio | C_19_H_18_O_7_ | CO |
| 475 | Rhein | C_15_H_8_O_6_ | CO |
| 476 | Rhein-8-glucoside | C_21_H_18_O_11_ | CO |
| 477 | Rubrofusarin-6-O-β-gentiobioside | C_26_H_30_O_15_ | CO |
| 478 | Rubrofusatin triglucoside | C_33_H_42_O_20_ | CO |
| 479 | Torachrysone tetraglucoside | C_38_H_54_O_24_ | CO |
| 480 | Torachrysone-8-O-β-D-gentiobioside | C_26_H_34_O_14_ | CO |
| 481 | Protocatechualdehyde | C_7_H_6_O_3_ | LB |
| 482 | Caffeic acid | C_9_H_8_O_4_ | LB |
| 483 | Catechin | C_15_H_16_O_7_ | LB |
| 484 | Epicatechin | C_15_H_14_O_6_ | LB |
| 485 | Ferulic Acid | C_10_H_10_O_4_ | LB |
| 486 | Rutinum | C_27_H_30_O_16_ | LB |
| 487 | Chlorogenic acid | C_16_H_18_O_9_ | LB |
| 488 | Lycibarbarspermidine A | C_31_H_43_N_3_O_11_ | LB |
| 489 | Lycibarbarspermidine B | C_31_H_43_N_3_O_12_ | LB |
| 490 | Lycibarbarspermidine C | C_31_H_43_N_3_O_13_ | LB |
| 491 | Lycibarbarspermidine D | C_31_H_43_N_3_O_14_ | LB |
| 492 | Lycibarbarspermidine E | C_31_H_43_N_3_O_15_ | LB |
| 493 | Withanolide A | C_28_H_38_O_6_ | LB |
| 494 | Withanolide B | C_28_H_38_O_5_ | LB |
| 495 | β-Carotene | C_40_H_56_ | LB |
| 496 | β-Cryptoxanthin | C_40_H_56_O | LB |
| 497 | Zeaxanthin | C_40_H_56_O_2_ | LB |
| 498 | Zeaxanthin monopalmitate | C_56_H_86_O_3_ | LB |
| 499 | Zeaxanthin dipalmitate | C_72_H_116_O_4_ | LB |
| 500 | Zeaxanthin monomyristate | C_54_H_82_O_3_ | LB |
| 501 | Zeaxanthin dimyristate | C_68_H_108_O_4_ | LB |
| 502 | β-Cryptoxanthin palmitate | C_56_H_86_O_2_ | LB |
| 503 | Lyciumamide A | C_36_H_36_N_2_O_8_ | LB |
| 504 | Lyciumamide B | C_36_H_36_N_2_O_8_ | LB |
| 505 | Lyciumamide C | C_28_H_29_NO_7_ | LB |
| 506 | Lyciumide A | C_18_H_19_NO_4_ | LB |
| 507 | Quercitrin | C_21_H_20_O_11_ | LB |
| 508 | Quercetin | C_15_H_10_O_7_ | LB |
| 509 | Rutin | C_27_H_30_O_16_ | LB |
| 510 | Narcissoside | C_28_H_32_O_16_ | LB |
| 511 | 3-O-Sophoroside-quercetin | C_27_H_30_O_17_ | LB |
| 512 | Isoquercitrin | C_21_H_20_O_12_ | LB |
| 513 | Linolenic acid | C_18_H_30_O_2_ | LB |
| 514 | Oleic acid | C_18_H_34_O_2_ | LB |
| 515 | Linoleic acid | C_18_H_32_O_2_ | LB |
| 516 | Lycibarbarphenylpropanoids A | C_21_H_28_O_13_ | LB |
| 517 | Lycibarbarphenylpropanoids B | C_21_H_28_O_13_ | LB |
| 518 | Lycibarbarphenylpropanoids C | C_22_H_30_O_14_ | LB |
| 519 | Lycibarbarphenylpropanoids D | C_22_H_30_O_14_ | LB |
| 520 | Lycibarbarphenylpropanoids E | C_22_H_32_O_13_ | LB |
| 521 | Lycibarbarphenylpropanoids F | C_23_H_32_O_13_ | LB |
| 522 | Lycibarbarphenylpropanoids G | C_23_H_32_O_13_ | LB |
| 523 | Lycibarbarphenylpropanoids H | C_24_H_34_O_14_ | LB |
| 524 | Lycibarbarphenylpropanoids I | C_24_H_34_O_14_ | LB |
| 525 | E-p-coumaric acid | C_9_H_8_O_3_ | LB |
| 526 | Emodin | C_15_H_10_O_5_ | LB |
| 527 | p-Hydroxybenzaldehyde | C_7_H_6_O_2_ | LB |
| 528 | (3S,4R)-6-hydroxy-4-(4-hydroxy-3,5-dimethoxyphenyl)-3-(hydroxymethyl)-N-(4-hydroxyphenethyl)-5,7-dimethoxy-3,4-dihydronaphthalene-2-carboxamide | C_30_H_33_NO_9_ | LB |
| 529 | (2S,3S,E)-3-{-2-(4-hydroxy-3,5-methoxyphenyl)-3-hydroxymethyl-2,3-dihydrobenzo[b][1,4]dioxin-6-yl}-N-(4-hydroxyphenethyl)-acrylamide | C_28_H_29_NO_8_ | LB |
| 530 | (2R,3R,E)-3-{-2-(4-hydroxy-3,5-methoxyphenyl)-3-hydroxymethyl-2,3-dihydrobenzo[b][1,4]dioxin-6-yl}-N-(4-hydroxyphenethyl)-acrylamide | C_28_H_29_NO_8_ | LB |
| 531 | N-trans-Feruloyltyramine | C_18_H_19_NO_4_ | LB |
| 532 | N-Acetyltyramine | C_9_H_13_NO_2_ | LB |
| 533 | N-p-trans-Coumaroyltyramine | C_17_H_17_NO_3_ | LB |
| 534 | Dihydro-N-Caffeoyl Tyramine | C_17_H_19_NO_4_ | LB |
| 535 | N-Caffeoyltyramine | C_17_H_17_NO_4_ | LB |
| 536 | Dihydro-feruloyl-5-methoxytyramine | C_19_H_23_NO_5_ | LB |
| 537 | 3-(4-hydroxy-3-methoxyphenyl)-N-[2-(4-hydroxyphenyl)-2-methoxyethyl]acrylamide | C_19_H_21_NO_5_ | LB |
| 538 | N-acetyl-N'-trans-feruloylputrescine | C_16_H_22_N_2_O_4_ | LB |
| 539 | erythro-canabisine H | C_28_H_31_NO_8_ | LB |
| 540 | grossamide K | C_28_H_29_NO_7_ | LB |
| 541 | (1,2-trans)-N3-(4-acetamidobutyl)-1-(3,4-dihydroxyphey)-7-hydroxy-N2-(4-hydroxyphenethyl)-6,8-dimethoxy-1,2-dihydronaphthalene-2,3-dicarboxamide | C_34_H_39_N_3_O_9_ | LB |
| 542 | 1,2-dihydro-6,8-dimethoxy-7-hydroxy-1-(3,4-dihydroxyphenyl)-N1,N2-bis[2-(4-hydroxyphenyl)ethyl]-2,3-naphthalene dicarboxamide | C_36_H_36_N_2_O_9_ | LB |
| 543 | Cannabisin F | C_36_H_36_N_2_O_8_ | LB |
| 544 | Cannabisin D | C_36_H_36_N_2_O_8_ | LB |
| 545 | (E)-2-(4,5-dihydroxy-2-{3-[(4-hydroxyphenethyl)amino]-3-oxopropyl}phenyl)-3-(4-hydroxy-3,5-dimethoxyphenyl)-N-(4-acetamidobutyl)acrylamide | C_34_H_41_N_3_O_9_ | LB |
| 546 | (E)-2-(4,5-dihydroxy-2-{3-[(4-hydroxyphenethyl)amino]-3-oxopropyl}phenyl)-3-(4-hydroxy-3-methoxyphenyl)-N-(4-acetamidobutyl)acrylamide | C_33_H_39_N_3_O_8_ | LB |
| 547 | (E)-2-(4,5-dihydroxy-2-{3-[(4-hydroxyphenethyl)amino]-3-oxopropyl}phenyl)-3-(4-hydroxy-3,5-dimethoxyphenyl)-N-(4-hydroxyphenethyl)acrylamide | C_36_H_38_N_2_O_9_ | LB |
| 548 | 3-Benzofurancarboxamide | C_36_H_36_N_2_O_8_ | LB |
| 549 | (+)-Lyoniresinol-3α-O-β-D-glucopyranoside | C_28_H_38_O_13_ | LB |
| 550 | Lipoxin | C_18_H_32_O_5_ | LB |
| 551 | Scopolamine | C_10_H_8_O_4_ | LB |
| 552 | 2,4-Dihydroxy-3,5-dimethoxybenzaldehyde | C_9_H_10_O_4_ | LB |
| 553 | Lycifuranone A | C_16_H_22_O_3_ | LB |
| 554 | Nobiletin | C_21_H_22_O_8_ | LB |
| 555 | 5-Hydroxymethylfurfural | C_6_H_6_O_3_ | LB |
| 556 | Scopoletin glucoside | C_16_H_18_O_9_ | LB |
| 557 | 2,6,2',6'-Tetramethoxy-4,4'-bis(2,3-epoxy-hydroxypropyl)biphenyl | C_22_H_26_O_8_ | LB |
| 558 | 4-（9H-β-carbolin-1-yl）-4-oxo-butyric acid | C_15_H_12_N_2_O_3_ | AA |
| 559 | Carboxymethyl isoferulate | C_12_H_12_O_6_ | AA |
| 560 | Isoferulic Acid | C_10_H_10_O_4_ | AA |
| 561 | Cirsiumaldehyde | C_12_H_10_O_5_ | AA |
| 562 | Daucossterol | C_35_H_60_O_6_ | AA |
| 563 | Succinic Acid | C_4_H_6_O_4_ | AA |
| 564 | Cinnamic acid | C_9_H_8_O_2_ | AA |
| 565 | 3,4-Dihydroxybenzaldehyde | C_7_H_6_O_3_ | AA |
| 566 | CiMigenol 3-beta-D-xylopyranoside | C_35_H_56_O_9_ | AA |
| 567 | Caffeic acid | C_9_H_8_O_4_ | AA |
| 568 | Adenosine | C_10_H_13_N_5_O_4_ | AA |
| 569 | Uridine | C_9_H_12_N_2_O_6_ | AA |
| 570 | Vanillic acid 4-β-D-glucoside | C_14_H_18_O_9_ | AA |
| 571 | Methyl chlorogenate | C_17_H_20_O_9_ | AA |
| 572 | 3-O-Feruloylquinic | C_17_H_20_O_9_ | AA |
| 573 | Esculetin | C_9_H_6_O_4_ | AA |
| 574 | 3,4-Dihydroxyhydrocinnamic acid | C_9_H_10_O_4_ | AA |
| 575 | Ferulic Acid | C_10_H_10_O_4_ | AA |
| 576 | Hydroferulic acid | C_10_H_12_O_4_ | AA |
| 577 | 5-Hydroxymethyl-2-furaldehyde | C_6_H_6_O_3_ | AA |
| 578 | 3,4-Dihydroxybenzyl alcohol | C_7_H_8_O_3_ | AA |
| 579 | isosinensetin | C_20_H_20_O_7_ | CR |
| 580 | sinensetin | C_20_H_20_O_7_ | CR |
| 581 | tangerine | C_20_H_20_O_7_ | CR |
| 582 | Gentiopicroside | C_27_H_32_O_14_ | CR |
| 583 | Hesperidin | C_28_H_34_O_15_ | CR |
| 584 | Nobiletin | C_21_H_22_O_8_ | CR |
| 585 | 3,5,6,7,8,3’,4’-Heptemthoxyflavone | C_22_H_24_O_9_ | CR |
| 586 | Tangeretin | C_20_H_20_O_7_ | CR |
| 587 | 3’,4’-Dihydroxy-7,5’-dimethoxyflavone | C_17_H_14_O_6_ | CR |
| 588 | 7-Hydroxy-5,6,8,4’-tetramethoxyflavone | C_19_H_18_O_7_ | CR |
| 589 | Monohydroxytrimethoxyflavone | C_18_H_16_O_6_ | CR |
| 590 | 3-Hydroxy-5,7,8-trimethoxyflavone | C_18_H_16_O_6_ | CR |
| 591 | 5-Hydroxy-7,8,3’,4’-tetramethoxyflavone | C_19_H_18_O_7_ | CR |
| 592 | 8-Hydroxy-5,6,7,3’,4’,5’-hexamethoxyflavone | C_21_H_22_O_9_ | CR |
| 593 | Monohydroxytrimethoxyflavone | C_18_H_16_O_6_ | CR |
| 594 | 6-Hydroxy-5,7,8,4’-tetramethoxyflavone | C_20_H_20_O_8_ | CR |
| 595 | 5-Hydroxy-3,6,7,8-tetramethoxyflavone | C_19_H_18_O_7_ | CR |
| 596 | 5,6,7,3’,4’-Pentamethoxyflavanone | C_20_H_22_O_7_ | CR |
| 597 | 7-Hydroxy-5,6,8,3’,4’-pentamethoxyflavone | C_20_H_20_O_8_ | CR |
| 598 | 3-Hydroxy-5,6,7,4’-tetramethoxyflavanone | C_19_H_20_O_7_ | CR |
| 599 | 5,7,8,3',4'-Pentamethoxyflavone | C_20_H_20_O_7_ | CR |
| 600 | Monohydroxytrimethoxyflavone | C_18_H_16_O_6_ | CR |
| 601 | 3’-Hydroxy-5,6,7,8,4'-pentamethoxyflavone/4’-Hydroxy-5,6,7,8,3'-pentamethoxyflavone | C_20_H_20_O_8_ | CR |
| 602 | 3- Hydroxy-5,6,7,8,3’,4’-hexamethoxyflavone | C_21_H_22_O_9_ | CR |
| 603 | Monohydroxypentamethoxyflavanone | C_20_H_22_O_8_ | CR |
| 604 | 5,7,3’,4’-Tetramethoxyflavone | C_17_H_14_O_6_ | CR |
| 605 | 5,7,8,3’,4’,5’-Hexamethoxyflavone | C_21_H_22_O_8_ | CR |
| 606 | 5,6,7,3',4'-Pentamethoxyflavone | C_20_H_20_O_7_ | CR |
| 607 | 5,6,7,4’-Tetramethoxyflavone | C_19_H_18_O_6_ | CR |
| 608 | 5,7,8,3’,4’-Pentamethoxyflavanone | C_20_H_22_O_7_ | CR |
| 609 | 5,6,7,3’,4’,5’-Hexamethoxyflavone | C_21_H_22_O_8_ | CR |
| 610 | 5,7,4’-Trimethoxyflavone | C_18_H_16_O_5_ | CR |
| 611 | 5,6,7,8,3',4'-Hexamethoxyflavanone | C_21_H_24_O_8_ | CR |
| 612 | 5,6,7,8,3',4'-Hexamethoxyflavone | C_21_H_22_O_8_ | CR |
| 613 | 5,7,8,4’-Tetramethoxyflavone | C_19_H_18_O_6_ | CR |
| 614 | 3,5,6,7,8,3',4'-Heptamethoxyflavone | C_22_H_24_O_9_ | CR |
| 615 | 5-Hydroxy-6,7,3’,4’-tetram ethoxyflavone | C_19_H_18_O_7_ | CR |
| 616 | 5-Hydroxy-6,7,8,3’,4’-pentamethoxyflavanone | C_20_H_22_O_8_ | CR |
| 617 | 5-Hydroxy-3,6,7,8,3’,4’-hexamethoxyflavone | C_21_H_22_O_9_ | CR |
| 618 | 5,6,7,8,4'-Pentamethoxyflavone | C_20_H_20_O_7_ | CR |
| 619 | 5-Hydroxy-6,7,8,3',4'-pentamethoxyflavone | C_20_H_20_O_8_ | CR |
| 620 | Hexamethoxyflavone | C_21_H_22_O_8_ | CR |
| 621 | Monohydroxytrimethoxyflavone | C_18_H_16_O_6_ | CR |
| 622 | 6,7,8,3’,4’-Pentamethoxyflvanone | C_20_H_22_O_7_ | CR |
| 623 | 5-Hydroxy-7,3’,4’-trimethoxyflavone | C_18_H_16_O_6_ | CR |
| 624 | 5,6,7,8,3’,4’-Hexamethoxyflvanone | C_21_H_24_O_8_ | CR |
| 625 | 5-Hydroxy-6,7,8,3’,4’,5’-hexamethoxyflavone(Gardenin A) | C_21_H_22_O_9_ | CR |
| 626 | 5-Hydroxy-6,7,8,4’-tetramethoxyflavone(Gardenin B) | C_19_H_18_O_7_ | CR |
| 627 | Pentamethoxyflvanone | C_20_H_22_O_7_ | CR |
| 628 | vicenin Ⅱ | C_27_H_30_O_15_ | CR |
| 629 | Apigenin 7-O-(2G-rhamnosyl)gentiobioside | C_33_H_40_O_19_ | CR |
| 630 | Orientin | C_21_H_20_O_11_ | CR |
| 631 | Eriocitrin | C_27_H_32_O_15_ | CR |
| 632 | Eriodictyol | C_15_H_12_O_6_ | CR |
| 633 | Vitexin | C_21_H_20_O_10_ | CR |
| 634 | Vicenin Ⅲ | C_26_H_28_O_14_ | CR |
| 635 | Kaempferol 3-rutinoside | C_27_H_30_O_15_ | CR |
| 636 | Isovitexin | C_21_H_20_O_10_ | CR |
| 637 | Prunin | C_21_H_22_O_10_ | CR |
| 638 | Naringin | C_27_H_32_O_14_ | CR |
| 639 | Naringenin chalcone | C_15_H_12_O_5_ | CR |
| 640 | Naringenin | C_15_H_12_O_5_ | CR |
| 641 | Hesperetin | C_16_H_14_O_6_ | CR |
| 642 | Apigenin | C_15_H_10_O_5_ | CR |
| 643 | Rhoifolin | C_27_H_30_O_14_ | CR |
| 644 | Hydroxygenkwanin | C_16_H_12_O_6_ | CR |
| 645 | Diosmin | C_28_H_32_O_15_ | CR |
| 646 | Neohesperidin | C_28_H_34_O_15_ | CR |
| 647 | Methyl hesperidin | C_29_H_36_O_15_ | CR |
| 648 | Isorhamnetin-3-O-neohespeidoside | C_28_H_32_O_16_ | CR |
| 649 | Homoplantaginin | C_22_H_22_O_11_ | CR |
| 650 | Pectolinarigenin | C_17_H_14_O_6_ | CR |
| 651 | (2S)-poncirenin | C_22_H_24_O_10_ | CR |
| 652 | Poncirin | C_28_H_34_O_14_ | CR |
| 653 | Isosakuranetin | C_16_H_14_O_5_ | CR |
| 654 | Luteolin | C_15_H_10_O_6_ | CR |
| 655 | Diosmetin | C_16_H_12_O_6_ | CR |
| 656 | Isorhamnetol | C_16_H_12_O_7_ | CR |
| 657 | Pureonebio | C_17_H_14_O_7_ | CR |
| 658 | Isosinensetin | C_20_H_20_O_7_ | CR |
| 659 | Sinensetin | C_20_H_20_O_7_ | CR |
| 660 | Eupatilin | C_18_H_16_O_7_ | CR |
| 661 | (+)-Pinocembrin | C_15_H_12_O_4_ | CR |
| 662 | Artemisetin | C_20_H_20_O_8_ | CR |
| 663 | 6-Demethoxytangeretin | C_19_H_18_O_6_ | CR |
| 664 | Chrysosplenetin | C_19_H_18_O_8_ | CR |
| 665 | Tangeretin | C_20_H_20_O_7_ | CR |
| 666 | Demethylnobiletin | C_20_H_20_O_8_ | CR |
| 667 | Gardenin B | C_19_H_18_O_7_ | CR |

Table S2. The screened candidate compounds in YGMM.

| No. | Retention Time | Adduct/Charge | Precursor Mass | Found At Mass | Mass Error (ppm) | Name | Formula | Source |
| --- | --- | --- | --- | --- | --- | --- | --- | --- |
| 1 | 0.56 | [M+H]+ | 313.055 | 313.0534 | -6.5 | Caffeic acid | C_13_H_12_O_9_ | CM, LB, AA, AS, LC |
| 2 | 0.59 | [M+H]+ | 519.171 | 519.172 | 2.2 | Lycibarbarphenylpropanoids D | C_22_H_30_O_14_ | LB |
| 3 | 0.59 | [M+H]+ | 601.192 | 601.1952 | 6.1 | Benzoylpaeoniflorin | C_30_H_32_O_13_ | RP |
| 4 | 0.6 | [M+K]+ | 423.069 | 423.0688 | -0.1 | Saikochromoside A | C_17_H_20_O_10_ | BC |
| 5 | 0.6 | [M+H]+ | 527.155 | 527.1578 | 5.7 | Salcolin B | C_27_H_26_O_11_ | BC |
| 6 | 0.6 | [M+H]+ | 545.132 | 545.135 | 4.9 | Paeoniflorin sulfite | C_23_H_28_O_13_S | RP |
| 7 | 0.6 | [M+FA-H]- | 591.1931 | 591.1937 | 1 | Lycibarbarphenylpropanoids H or Lycibarbarphenylpropanoids I | C_24_H_34_O_14_ | LB |
| 8 | 0.6 | [M+FA-H]- | 629.1876 | 629.1913 | 6 | Benzoylalbiflorin | C_30_H_32_O_12_ | RP |
| 9 | 0.62 | [M+K]+ | 384.121 | 384.12 | -2.1 | Dihydro-feruloyl-5-methoxytyramine | C_19_H_23_NO_5_ | LB |
| 10 | 0.62 | [M-H]- | 939.1109 | 939.1119 | 1.1 | 1,2,3,4,6-penta-O-galloyl-β-D- glucopyranoside | C_41_H_32_O_26_ | RP |
| 11 | 0.63 | [M-H]- | 343.1187 | 343.1184 | -0.9 | 5,7-Dihydroxyl-6-methyl-8,4'-dimethoxyl homoisoflavanone | C_19_H_20_O_6_ | PO |
| 12 | 0.64 | [M-H]- | 341.1089 | 341.1084 | -1.5 | Sucralose | C_12_H_22_O_11_ | RP |
| 13 | 0.64 | [M+FA-H]- | 389.1242 | 389.1214 | -7.3 | 5,7,4'-trihydroxy ，3’-methoxyl-6,8-dimethyl-homisoflavanone. | C_19_H_20_O_6_ | OJ |
| 14 | 0.64 | [M-H]- | 389.1242 | 389.1214 | -7.3 | Monohydroxypentamethoxyflavanone | C_20_H_22_O_8_ | CR |
| 15 | 0.64 | [M+H]+ | 509.144 | 509.1487 | 8.8 | (-)-(5S,6S)-5,6-Dihydro-3,8,10-trihydroxy-5-(4-hydroxy-3-methoxyphenyl)-6-hydroxymethyl-2,4-dimethoxy7H-benzo[c]xanthen-7-one | C_27_H_24_O_10_ | BC |
| 16 | 0.65 | [M+FA-H]- | 343.0823 | 343.0812 | -3.4 | 5,7-dihydroxy-6-dimethyl-3-(4'-hydroxybenzyl) chromone or 5,7-dihydroxy-8-dimethyl-3-(4'-hydroxybenzyl) chromone | C_17_H_14_O_5_ | OJ |
| 17 | 0.65 | [M-H]- | 523.1821 | 523.1775 | -8.8 | 6-O-E-Feruloylajugol | C_25_H_32_O_12_ | RG |
| 18 | 0.66 | [M+Cl]- | 377.0434 | 377.0406 | -7.2 | 5,7-dihydroxy-6,8-dialdehyde-3-(4'- methoxybenzyl) chromone | C_18_H_14_O_7_ | OJ |
| 19 | 0.66 | [M+Cl]- | 437.1009 | 437.1017 | 1.9 | Nobiletin | C_21_H_22_O_8_ | LB |
| 20 | 0.67 | [M-H]- | 203.0197 | 203.0199 | 1 | Malonic acid | C_7_H_8_O_7_ | CM |
| 21 | 0.68 | [M+H]+ | 315.123 | 315.1229 | 0.6 | (3R)-5,7-Dihydroxyl-6,8-dimethyl-3-(4'-hydroxylbenzyl)-chroman-4-one | C_18_H_18_O_5_ | PO |
| 22 | 0.69 | [M+Cl]- | 515.1326 | 515.1318 | -1.5 | AlbiflorinR1 | C_23_H_28_O_11_ | RP |
| 23 | 0.7 | [M+H]+ | 163.063 | 163.0612 | -9.9 | Trans-p-coumaramide | C_9_H_8_NO_2_ | PO |
| 24 | 0.7 | [M+Cl]- | 495.1275 | 495.1321 | 9.4 | Aplopaeonoside or Diglucosyl Gallic Acid | C_20_H_28_O_12_ | RP |
| 25 | 0.70 | [M+Na]+ | 709.216 | 709.213 | -4.4 | Rehmannioside D | C_27_H_42_O_20_ | RG |
| 26 | 0.71 | [M-H]- | 331.0671 | 331.0673 | 0.6 | Glucopyranose gallate | C_13_H_16_O_10_ | RP |
| 27 | 0.72 | [M+H]+ | 168.102 | 168.1023 | 2.1 | N-Acetyltyramine | C_9_H_13_NO_2_ | LB |
| 28 | 0.72 | [M+FA-H]- | 312.095 | 312.0946 | -1.3 | Adenosine | C_10_H_13_N_5_O_4_ | AA, RP, RG |
| 29 | 0.73 | [M-H]- | 141.0193 | 141.0198 | 3.3 | 5-Hydroxymethyl-2-furoic acid | C_6_H_6_O_4_ | RG |
| 30 | 0.83 | [M+Cl]- | 822.0688 | 822.0762 | 9 | 1,2,3,6-Tetra-O-galloyl-β-D-glucose | C_34_H_27_O_22_ | RP |
| 31 | 0.84 | [M+FA-H]- | 833.1054 | 833.1056 | 0.2 | 1,2,3,6-Tetragalloylglucose | C_34_H_28_O_22_ | RP |
| 32 | 0.85 | [M+K]+ | 415.1 | 415.0995 | -1.4 | 8-Epiloganic acid | C_16_H_24_O_10_ | RG |
| 33 | 0.87 | [M+H]+ | 136.062 | 136.062 | 2 | Adenine | C_5_H_5_N_5_ | LC |
| 34 | 0.88 | [M+H]+ | 165.055 | 165.0551 | 2.8 | E-p-coumaric acid | C_9_H_8_O_3_ | LB |
| 35 | 0.88 | [M+Na]+ | 215.016 | 215.0167 | 2.1 | Citric acid | C_6_H_8_O_7_ | RP, RG |
| 36 | 0.88 | [M+H]+ | 284.099 | 284.0995 | 1.8 | Guanosine | C_10_H_13_N_5_O_5_ | RG |
| 37 | 0.90 | [M-H]- | 243.0623 | 243.0623 | 0.2 | Uridine | C_9_H_12_N_2_O_6_ | RG, AA |
| 38 | 0.91 | [M+H]+ | 194.081 | 194.0813 | 0.7 | Trans-ferulamide | C_10_H_11_NO_3_ | PO |
| 39 | 0.91 | [M-H]- | 506.182 | 506.1795 | -5.1 | (2R,3R,E)-3-{-2-(4-hydroxy-3,5-methoxyphenyl)-3-hydroxymethyl-2,3-dihydrobenzo[b][1,4]dioxin-6-yl}-N-(4-hydroxyphenethyl)-acrylamide | C_28_H_29_NO_8_ | LB |
| 40 | 0.98 | [M-H]- | 117.0193 | 117.0193 | -0.3 | Succinic Acid | C_4_H_6_O_4_ | AA |
| 41 | 1.04 | [M+Cl]- | 466.0672 | 466.0701 | 6.2 | Emodin-6-glucoside | C_21_H_19_O_10_ | CO |
| 42 | 1.06 | [M+H]+ | 171.029 | 171.029 | 1.3 | Gallic acid | C_7_H_6_O_5_ | BC, RP, LC |
| 43 | 1.07 | [M+H]+ | 132.102 | 132.102 | 0.6 | Isoleucine | C_6_H_13_NO_2_ | AS |
| 44 | 1.07 | [M+K]+ | 401.084 | 401.084 | -1.2 | Monomelittoside | C_15_H_22_O_10_ | RG |
| 45 | 1.08 | [M-H]- | 130.0874 | 130.0874 | 0.6 | leucine | C_6_H_13_NO_2_ | AS |
| 46 | 1.09 | [M+FA-H]- | 171.0299 | 171.0299 | 0.2 | 5-Hydroxymethylfurfural | C_6_H_6_O_3_ | LB, RG |
| 47 | 1.16 | [M-H]- | 195.0663 | 195.0662 | -0.6 | paeonilactone B | C_10_H_12_O_4_ | RP |
| 48 | 1.25 | [M-H]- | 137.0244 | 137.0245 | 0.6 | 4-Hydroxybenzoic acid | C_7_H_6_O_3_ | RP |
| 49 | 1.26 | [M-H]- | 183.0299 | 183.03 | 0.5 | Methyl gallate | C_8_H_8_O_5_ | RP |
| 50 | 1.28 | [M+FA-H]- | 243.0874 | 243.0874 | -0.2 | Paeonisuffrone | C_10_H_14_O_4_ | RP |
| 51 | 1.64 | [M-H]- | 147.0452 | 147.0453 | 0.7 | Cinnamic acid | C_9_H_8_O_2_ | AA |
| 52 | 1.66 | [M+H]+ | 127.039 | 127.0391 | 0.8 | 5-Hydroxymethyl-2-furaldehyde | C_6_H_6_O_3_ | AA |
| 53 | 1.66 | [M+H]+ | 127.039 | 127.0391 | 0.8 | Pyrogallol | C_6_H_6_O_3_ | RP |
| 54 | 1.85 | [M-H]- | 167.035 | 167.0351 | 0.6 | Vanillic acid | C_8_H_8_O_4_ | AS, LC |
| 55 | 1.94 | [M+FA-H]- | 185.0455 | 185.0457 | 1 | 3,4-Dihydroxybenzyl alcohol | C_7_H_8_O_3_ | AA |
| 56 | 1.96 | [M+H]+ | 155.034 | 155.034 | 0.8 | 3,4-Dihydroxybenzoic acid | C_7_H_6_O_4_ | RP |
| 57 | 1.96 | [M+H]+ | 155.034 | 155.034 | 0.8 | protocatechuic acid | C_7_H_6_O_4_ | LC |
| 58 | 1.99 | [M-H]- | 153.0193 | 153.0195 | 0.8 | Gentisic acid or Protocatechuic acid | C_7_H_6_O_4_ | RG |
| 59 | 2.01 | [M+FA-H]- | 373.114 | 373.1141 | 0.3 | Paeonoside | C_15_H_20_O_8_ | RP |
| 60 | 2.12 | [M+FA-H]- | 493.0988 | 493.1019 | 6.5 | Astragaloside | C_21_H_20_O_11_ | RP |
| 61 | 2.39 | [M+H]+ | 163.039 | 163.0389 | -0.4 | 6-Hydroxycoumarin | C_9_H_6_O_3_ | RP |
| 62 | 2.4 | [M+FA-H]- | 399.0933 | 399.0931 | -0.5 | 1-Caffeoylquinic acid | C_16_H_18_O_9_ | CM |
| 63 | 2.4 | [M+FA-H]- | 399.0933 | 399.0931 | -0.5 | Chlorogenic acid | C_16_H_18_O_9_ | LB, BC, AS, LC, CM |
| 64 | 2.52 | [M-H]- | 329.0878 | 329.0877 | -0.2 | Leontoside A | C_14_H_18_O_9_ | RP |
| 65 | 2.63 | [M+FA-H]- | 393.1402 | 393.1403 | 0.1 | Leonuride | C_15_H_24_O_9_ | RG |
| 66 | 2.64 | [M-H]- | 219.0663 | 219.0678 | 6.8 | Senkyunolide D | C_12_H_12_O_4_ | LC |
| 67 | 2.73 | [M-H]- | 197.0455 | 197.0456 | 0.2 | Ethyl gallate | C_9_H_10_O_5_ | RP |
| 68 | 2.92 | [M-H]- | 347.1348 | 347.1347 | 0 | Afrormosine | C_15_H_24_O_9_ | RG |
| 69 | 3.06 | [M-H]- | 461.1664 | 461.1663 | -0.4 | Decaffeoyl-verbascoside or Forsythoside E | C_20_H_30_O_12_ | RG |
| 70 | 3.13 | [M-H]- | 137.0244 | 137.0243 | -0.9 | p-Hydroxybenzoicacid | C_7_H_6_O_3_ | LC |
| 71 | 3.31 | [M-H]- | 343.1398 | 343.14 | 0.4 | Mudanpioside G | C_16_H_24_O_8_ | RP |
| 72 | 3.32 | [M+FA-H]- | 183.0299 | 183.0316 | 9.3 | Protocatechualdehyde | C_7_H_6_O_3_ | LB, RP |
| 73 | 3.38 | [M+FA-H]- | 345.1191 | 345.1192 | 0.3 | Salidroside | C_14_H_20_O_7_ | RG |
| 74 | 3.47 | [M+FA-H]- | 391.1246 | 391.1231 | -3.8 | Aucubin | C_15_H_22_O_9_ | RG |
| 75 | 3.77 | [M+FA-H]- | 533.1512 | 533.1517 | 0.9 | Lycibarbarphenylpropanoids A or Lycibarbarphenylpropanoids B | C_21_H_28_O_13_ | LB |
| 76 | 4.02 | [M+H]+ | 497.165 | 497.1657 | 0.8 | 4"-Hydroxyl-albiflorin | C_23_H_28_O_12_ | RP |
| 77 | 4.03 | [M+Na]+ | 519.147 | 519.1453 | -3.9 | Gentiopicroside | C_23_H_28_O_12_ | RP |
| 78 | 4.06 | [M-H]- | 361.114 | 361.112 | -5.6 | Catalpol | C_15_H_22_O_10_ | RG |
| 79 | 4.57 | [M+FA-H]- | 227.0561 | 227.055 | -5 | 2,4-Dihydroxy-3,5-dimethoxybenzaldehyde | C_9_H_10_O_4_ | LB |
| 80 | 4.64 | [M-H]- | 181.0506 | 181.0507 | 0.6 | 3,4-Dihydroxyhydrocinnamic acid | C_9_H_10_O_4_ | AA |
| 81 | 4.69 | [M+FA-H]- | 197.0455 | 197.046 | 2.2 | Vanillin | C_8_H_8_O_3_ | LC |
| 82 | 4.7 | [M+H]+ | 179.034 | 179.0343 | 2.2 | Esculetin | C_9_H_6_O_4_ | AA |
| 83 | 4.72 | [M+H]+ | 355.102 | 355.1022 | -0.5 | Scopoletin glucoside | C_16_H_18_O_9_ | LB |
| 84 | 4.74 | [M-H]- | 421.0776 | 421.076 | -3.8 | Norswertianolin | C_19_H_18_O_11_ | CM |
| 85 | 4.88 | [M-H]- | 457.1715 | 457.1717 | 0.3 | Ailexperphenoside A | C_21_H_30_O_11_ | RP |
| 86 | 4.96 | [M-H]- | 135.0928 | 135.0929 | 1.1 | Tetramethylpyrazine | C_8_H_12_N_2_ | LC |
| 87 | 5.12 | [M-H]- | 121.0295 | 121.0296 | 1 | p-Hydroxybenzaldehyde | C_7_H_6_O_2_ | LB |
| 88 | 5.31 | [M-H]- | 369.0827 | 369.0829 | 0.4 | Neochlorogenicacid | C_16_H_18_O_10_ | CM |
| 89 | 5.34 | [M+FA-H]- | 167.035 | 167.0348 | -0.8 | Benzoic acid | C_7_H_6_O_2_ | RP |
| 90 | 5.5 | [M-H]- | 163.0401 | 163.0401 | 0.5 | p-Coumaric acid | C_9_H_8_O_3_ | AS |
| 91 | 5.53 | [M-H]- | 367.1035 | 367.1036 | 0.3 | 3-O-Feruloylquinic or Methyl chlorogenate | C_17_H_20_O_9_ | AA |
| 92 | 5.57 | [M+FA-H]- | 563.1618 | 563.1621 | 0.6 | Lycibarbarphenylpropanoids C | C_22_H_30_O_14_ | LB |
| 93 | 5.72 | [M-H]- | 345.1555 | 345.1554 | -0.3 | Rehmapicroside | C_16_H_26_O_8_ | RG |
| 94 | 5.98 | [M+H]+ | 235.06 | 235.0603 | 0.9 | Cirsiumaldehyde | C_12_H_10_O_5_ | AA |
| 95 | 7.85 | [M-H]- | 533.0937 | 533.0942 | 1 | Luteolin-7-O-6''-malonyl-glucoside | C_24_H_22_O_14_ | CM |
| 96 | 8.05 | [M+H]+ | 627.156 | 627.156 | 0.8 | 3-O-Sophoroside-quercetin | C_27_H_30_O_17_ | LB |
| 97 | 8.9 | [M-H]- | 223.0976 | 223.0976 | -0.1 | Senkyunolide H | C_12_H_16_O_4_ | LC |
| 98 | 8.9 | [M-H]- | 223.0976 | 223.0976 | -0.1 | Senkyunolide H | C_12_H_16_O_4_ | AS |
| 99 | 9.13 | [M-H]- | 119.0502 | 119.0501 | -1 | 1-Phenyl-Ethanone | C_8_H_8_O | AS |
| 100 | 10.63 | [M+H]+ | 411.165 | 411.1642 | -1.9 | Chlorogenic acid butyl ester | C_20_H_26_O_9_ | CM |
| 101 | 10.64 | [M+H]+ | 227.128 | 227.1277 | -0.3 | Senkyunolide J | C_12_H_18_O_4_ | LC |
| 102 | 10.69 | [M+FA-H]- | 678.2668 | 678.2625 | -6.4 | (1,2-trans)-N3-(4-acetamidobutyl)-1-(3,4-dihydroxyphey)-7-hydroxy-N2-(4-hydroxyphenethyl)-6,8-dimethoxy-1,2-dihydronaphthalene-2,3-dicarboxamide | C_34_H_39_N_3_O_9_ | LB |
| 103 | 10.72 | [M-H]- | 387.1661 | 387.1662 | 0.4 | Tuberonic acid glucoside | C_18_H_28_O_9_ | CM |
| 104 | 10.8 | [M+H]+ | 197.081 | 197.0809 | 0.5 | Hydroferulic acid | C_10_H_12_O_4_ | AA |
| 105 | 10.8 | [M+Na]+ | 503.152 | 503.1523 | -0.2 | Albiflorin | C_23_H_28_O_11_ | RP |
| 106 | 11.29 | [M-H]- | 641.2087 | 641.2083 | -0.7 | β-gentiobiosyl paeoniflorin | C_29_H_38_O_16_ | RP |
| 107 | 11.56 | [M+H]+ | 634.297 | 634.2965 | -0.9 | Lycibarbarspermidine A | C_31_H_43_N_3_O_11_ | LB |
| 108 | 11.74 | [M-H]- | 225.1132 | 225.1133 | 0.2 | Chuanxiongnolide Ｒ2 | C_12_H_18_O_4_ | LC |
| 109 | 12.39 | [M+H]+ | 193.05 | 193.0494 | -0.8 | Scopoletin | C_10_H_8_O_4_ | LC |
| 110 | 12.4 | [M+Na]+ | 215.031 | 215.0317 | 1 | 3-Carboxyrthyl-phthalide | C_10_H_8_O_4_ | LC |
| 111 | 12.4 | [M+Na]+ | 215.031 | 215.0317 | 1 | Scopolamine | C_10_H_8_O_4_ | LB |
| 112 | 12.8 | [M-H]- | 635.089 | 635.0886 | -0.6 | 1,3,6-Tri-O-galloyl-beta-D-glucose | C_27_H_24_O_18_ | RP |
| 113 | 12.81 | [M+FA-H]- | 742.2676 | 742.2717 | 5.4 | Lycibarbarspermidine E | C_31_H_43_N_3_O_15_ | LB |
| 114 | 13.2 | [M-H]- | 479.1559 | 479.1555 | -0.7 | Mudanpioside I or Paeoniflorin | C_23_H_28_O_11_ | RP |
| 115 | 13.21 | [M+H]+ | 301.107 | 301.1074 | 1.2 | (3R)-5,7-Dihydroxy-6-me-thyl-3-(4′-hydroxybenzyl)-chroman-4-one | C_17_H_16_O_5_ | PO |
| 116 | 13.21 | [M+H]+ | 463.16 | 463.1604 | 1.2 | Lactiflorin | C_23_H_26_O_10_ | RP |
| 117 | 13.21 | [M+H]+ | 741.224 | 741.228 | 5.9 | Apigenin 7-O-(2G-rhamnosyl)gentiobioside | C_33_H_40_O_19_ | CR |
| 118 | 13.21 | [M+Na]+ | 965.305 | 965.3048 | -0.2 | Paeonidanin E | C_46_H_54_O_21_ | RP |
| 119 | 13.23 | [M-H]- | 569.1876 | 569.1873 | -0.5 | Torachrysone-8-O-β-D-gentiobioside | C_26_H_34_O_14_ | CO |
| 120 | 13.26 | [M-H]- | 193.0506 | 193.0507 | 0.5 | Ferulic Acid | C_10_H_10_O_4_ | LB, AS, LC |
| 121 | 13.26 | [M-H]- | 193.0506 | 193.0507 | 0.5 | Ferulic Acid or Isoferulic Acid | C_10_H_10_O_4_ | AA |
| 122 | 13.69 | [M-H]- | 241.1081 | 241.108 | -0.8 | 7-Dihydroxy-3-butylphthalide | C_12_H_18_O_5_ | LC |
| 123 | 13.81 | [M+Cl]- | 629.1279 | 629.1257 | -3.4 | Kaempferol 3-rutinoside | C_27_H_30_O_15_ | CR |
| 124 | 13.82 | [M+H]+ | 595.166 | 595.1654 | -0.6 | Emodin-6-O-β-gentiobioside | C_27_H_30_O_15_ | CO |
| 125 | 13.83 | [M+K]+ | 633.122 | 633.1166 | -7.9 | 6,8-C,C-diglucosylapigenin isomer or Luteolin 7-O-rutinoside | C_27_H_30_O_15_ | CM |
| 126 | 14.99 | [M+Cl]- | 583.086 | 583.0897 | 6.4 | Diosmetin-7-O-6''-malonyl-glucoside | C_25_H_24_O_14_ | CM |
| 127 | 17.01 | [M+H]+ | 625.176 | 625.1758 | -0.8 | Narcissoside | C_28_H_32_O_16_ | LB, BC |
| 128 | 17.03 | [M+FA-H]- | 623.1618 | 623.1612 | -0.9 | Kaempferitrin | C_27_H_30_O_14_ | RP |
| 129 | 17.25 | [M+FA-H]- | 669.1672 | 669.1687 | 2.2 | Isorhamnetin-3-O-neohespeidoside | C_28_H_32_O_16_ | CR |
| 130 | 19.04 | [M-H]- | 393.1191 | 393.1189 | -0.5 | 6-Hydroxymusizin-8-O-β-D-glucoside | C_19_H_22_O_9_ | CO |
| 131 | 19.64 | [M-H]- | 563.1406 | 563.1404 | -0.5 | Vicenin Ⅲ | C_26_H_28_O_14_ | CR |
| 132 | 20.02 | [M+H]+ | 209.117 | 209.1174 | 1 | Senkyunolide G | C_12_H_16_O_3_ | LC |
| 133 | 20.02 | [M+H]+ | 209.117 | 209.1174 | 1 | Senkyunolide G | C_12_H_16_O_3_ | AS |
| 134 | 20.81 | [M+FA-H]- | 463.1246 | 463.1241 | -1.1 | C14 | C_21_H_22_O_9_ | CR |
| 135 | 20.96 | [M-H]- | 417.1191 | 417.1189 | -0.6 | Liquiritin | C_21_H_22_O_9_ | BC |
| 136 | 21.72 | [M-H]- | 239.0925 | 239.0923 | -0.8 | Senkyunolide O | C_12_H_16_O_5_ | LC |
| 137 | 21.8 | [M+K]+ | 503.059 | 503.0567 | -3.9 | Isoquercitrin | C_21_H_20_O_12_ | BC, LB |
| 138 | 21.8 | [M+K]+ | 503.059 | 503.0567 | -3.9 | Quercetin-7-O-galactoside or Quercetin-3-O-glucoside | C_21_H_20_O_12_ | CM |
| 139 | 22.26 | [M+FA-H]- | 333.0616 | 333.0587 | -8.7 | Eriodictyol | C_15_H_12_O_6_ | CR |
| 140 | 22.33 | [M-H]- | 449.1089 | 449.1085 | -0.9 | Isookanin-7-O-β-diglucopyranoside | C_21_H_22_O_11_ | CM |
| 141 | 22.61 | [M+FA-H]- | 499.3429 | 499.3405 | -4.9 | Betulonicacid | C_30_H_46_O_3_ | RP |
| 142 | 23.11 | [M+Na]+ | 367.136 | 367.1345 | -5 | Mudanpioside F | C_16_H_24_O_8_ | RP |
| 143 | 24.67 | [M-H]- | 310.1085 | 310.1088 | 1 | (E)-3-(4-Hydroxy-3-methoxybenzylidene)-4-(4-hydroxyphenyl)pyrrolidin-2-one | C_18_H_17_NO_4_ | PO |
| 144 | 24.67 | [M-H]- | 328.119 | 328.1189 | -0.6 | N-cis-feruloyloctopamine or N-trans-feruloyloctopamine | C_18_H_19_NO_5_ | PO |
| 145 | 24.87 | [M+H]+ | 303.05 | 303.0501 | 0.7 | Quercetin | C_15_H_10_O_7_ | LB |
| 146 | 24.89 | [M-H]- | 609.1461 | 609.145 | -1.8 | Rutin | C_27_H_30_O_16_ | BC |
| 147 | 24.89 | [M+Cl]- | 645.1228 | 645.1224 | -0.7 | Rutin or Rutinum | C_27_H_30_O_16_ | LB |
| 148 | 24.9 | [M+H]+ | 611.161 | 611.1601 | -1 | Kaempferol 3,7-O-di-β-D-glucopyranside | C_27_H_30_O_16_ | RP |
| 149 | 25.19 | [M+K]+ | 501.043 | 501.0386 | -8.8 | KaeMpferol 3-O-D-glucopyranoside | C_21_H_18_O_12_ | RP |
| 150 | 25.21 | [M-H]- | 461.0725 | 461.0721 | -1 | Luteolin-7-O-glucuronide | C_21_H_18_O_12_ | CM |
| 151 | 26.49 | [M-H]- | 447.0933 | 447.0928 | -1.1 | Luteoloside or Luteolin-7-O-glucoside | C_21_H_20_O_11_ | CM |
| 152 | 26.49 | [M+FA-H]- | 493.0988 | 493.0985 | -0.6 | Alaternin-2-O-β-D-glucoside | C_21_H_20_O_11_ | CO |
| 153 | 26.49 | [M+FA-H]- | 493.0988 | 493.0985 | -0.6 | Orientin | C_21_H_20_O_11_ | CR |
| 154 | 26.52 | [M+H]+ | 287.055 | 287.0552 | 0.8 | Alaternin or Citreorosein | C_15_H_10_O_6_ | CO |
| 155 | 26.52 | [M+H]+ | 287.055 | 287.0552 | 0.8 | Kaempferol | C_15_H_10_O_6_ | RP |
| 156 | 26.52 | [M+H]+ | 287.055 | 287.0552 | 0.8 | Luteolin | C_15_H_10_O_6_ | BC, RG, CM |
| 157 | 26.79 | [M-H]- | 785.251 | 785.2502 | -1 | Echinacoside or Purpureaside B | C_35_H_46_O_20_ | RG |
| 158 | 27.68 | [M-H]- | 165.0557 | 165.0556 | -0.8 | Paeonol | C_9_H_10_O_3_ | RP |
| 159 | 27.85 | [M-H]- | 893.2932 | 893.2925 | -0.8 | Torachrysone tetraglucoside | C_38_H_54_O_24_ | CO |
| 160 | 28.53 | [M-H]- | 187.0976 | 187.0974 | -0.9 | Anchoicacid | C_9_H_16_O_4_ | AS |
| 161 | 28.71 | [M+Na]+ | 247.094 | 247.0938 | -0.9 | Senkyunolide I | C_12_H_16_O_4_ | AS |
| 162 | 28.79 | [M+Na]+ | 229.084 | 229.0855 | 8.5 | (Z)-6,7-epoxyligustilide or 2-Valeryl-benzoic acid | C_12_H_14_O_3_ | AS |
| 163 | 28.79 | [M+Na]+ | 229.084 | 229.0855 | 8.5 | 4-Hydroxy-3-butylphthalide | C_12_H_14_O_3_ | LC |
| 164 | 28.79 | [M-H]- | 783.1778 | 783.1779 | 0.1 | 3', 6'-Di-O-gal-loylpaeoniflorin | C_37_H_36_O_19_ | RP |
| 165 | 29.52 | [M-H]- | 627.1931 | 627.1924 | -1 | Cassia-lactone gentiobioside | C_28_H_36_O_16_ | CO |
| 166 | 30.42 | [M-H]- | 251.0561 | 251.0559 | -0.8 | 3-(4-Hydroxy-3-methoxy-phenyl)-acrylic acid carbox-ymethyl ester | C_12_H_12_O_6_ | PO |
| 167 | 30.42 | [M-H]- | 251.0561 | 251.0559 | -0.8 | Carboxymethyl isoferulate | C_12_H_12_O_6_ | AA |
| 168 | 30.67 | [M-H]- | 799.2666 | 799.2659 | -0.9 | Jionoside A1 | C_36_H_48_O_20_ | RG |
| 169 | 30.75 | [M-H]- | 271.0612 | 271.0611 | -0.2 | Naringenin chalcone | C_15_H_12_O_5_ | CR |
| 170 | 31.63 | [M-H]- | 163.0765 | 163.0763 | -1 | Eugenin | C_10_H_12_O_2_ | BC |
| 171 | 32.2 | [M+FA-H]- | 561.125 | 561.1252 | 0.4 | 3,4-Di-caffeoyl-quinic acid or 4,5-Dicaffeoylquinic acid | C_25_H_24_O_12_ | CM |
| 172 | 32.33 | [M-H]- | 515.1195 | 515.1189 | -1.2 | Isochlorogenic acid B | C_25_H_24_O_12_ | CM, BC |
| 173 | 32.54 | [M+Cl]- | 615.1486 | 615.1482 | -0.7 | Naringin | C_27_H_32_O_14_ | CR |
| 174 | 33.01 | [M+H]+ | 447.092 | 447.0924 | 0.5 | Rhein-8-glucoside | C_21_H_18_O_11_ | CO |
| 175 | 33.01 | [M+Na]+ | 469.074 | 469.0749 | 1.7 | Apigenin-7-O-glucuronide or Apigenin-4'-O-glucopyranoside | C_21_H_18_O_11_ | CM |
| 176 | 33.01 | [M+Na]+ | 469.074 | 469.0749 | 1.7 | Baicalin | C_21_H_18_O_11_ | BC |
| 177 | 33.19 | [M+FA-H]- | 477.1038 | 477.1011 | -5.9 | Aloe-emodin-8-O-β-D-glucoside | C_21_H_20_O_10_ | CO |
| 178 | 33.19 | [M+FA-H]- | 477.1038 | 477.1011 | -5.9 | Isovitexin | C_21_H_20_O_10_ | CR |
| 179 | 33.33 | [M+H]+ | 271.06 | 271.0603 | 0.6 | Aloe-emodin | C_15_H_10_O_5_ | CO |
| 180 | 33.33 | [M+H]+ | 433.113 | 433.1124 | -1.1 | Vitexin | C_21_H_20_O_10_ | CR |
| 181 | 33.34 | [M+K]+ | 471.069 | 471.0686 | -0.5 | β-D-glucopyranosyl benzoate | C_21_H_20_O_10_ | RP |
| 182 | 33.35 | [M-H]- | 431.0984 | 431.0979 | -1.1 | Puerarin | C_21_H_20_O_10_ | BC |
| 183 | 33.52 | [M+H]+ | 221.081 | 221.081 | 0.6 | Senkyunolide E | C_12_H_12_O_4_ | LC |
| 184 | 34.13 | [M+H]+ | 139.039 | 139.0383 | -5 | 3,4-Dihydroxybenzaldehyde | C_7_H_6_O_3_ | AA |
| 185 | 34.13 | [M+H]+ | 139.039 | 139.0383 | -5 | p-hydroxybenzoic acid | C_7_H_6_O_3_ | RG |
| 186 | 34.53 | [M+K]+ | 617.127 | 617.1246 | -3.5 | Apigenin-7-O-rutinoside | C_27_H_30_O_14_ | CM |
| 187 | 35.89 | [M-H]- | 623.1981 | 623.1975 | -1 | Acteoside or Isoacteoside | C_29_H_36_O_15_ | RG |
| 188 | 35.91 | [M+FA-H]- | 669.2036 | 669.2036 | 0 | Methyl hesperidin | C_29_H_36_O_15_ | CR |
| 189 | 36.04 | [M-H]- | 255.0663 | 255.0661 | -0.8 | (+)-Pinocembrin | C_15_H_12_O_4_ | CR |
| 190 | 37.17 | [M+H]+ | 479.118 | 479.119 | 1.3 | 1-Desmethyl- aurantio-obtusin-2- O-β-D- glucopyranoside | C_22_H_22_O_12_ | CO |
| 191 | 37.23 | [M-H]- | 301.0718 | 301.0715 | -0.7 | (3R)-5,7-Dihydroxyl-3-(2',4'-dihydroxylbenzyl)-chroman-4-one | C_16_H_14_O_6_ | PO |
| 192 | 37.23 | [M-H]- | 301.0718 | 301.0715 | -0.7 | Hesperetin | C_16_H_14_O_6_ | CR |
| 193 | 37.23 | [M+FA-H]- | 577.1199 | 577.1187 | -2.1 | Acacetin-7-O-6''-malonyl-galactoside | C_25_H_24_O_13_ | CM |
| 194 | 37.63 | [M+H]+ | 303.086 | 303.0863 | -0.2 | 5,7,2',4'-Tetrahydroxyl homoisoflavanone | C_16_H_14_O_6_ | PO |
| 195 | 37.63 | [M+H]+ | 449.144 | 449.1439 | -0.8 | (2S)-poncirenin | C_22_H_24_O_10_ | CR |
| 196 | 37.63 | [M+H]+ | 611.197 | 611.196 | -1.7 | Neohesperidin | C_28_H_34_O_15_ | CR |
| 197 | 37.63 | [M+H]+ | 649.159 | 649.1537 | -7.5 | Benzoylpaeoniflorin sulfonate | C_30_H_32_O_14_S | RP |
| 198 | 37.68 | [M-H]- | 631.1668 | 631.1665 | -0.6 | Galloylpaeoniflorin | C_30_H_32_O_15_ | RP |
| 199 | 37.79 | [M-H]- | 475.0882 | 475.0885 | 0.6 | Diosmetin 7-glucuronide | C_22_H_20_O_12_ | CM |
| 200 | 37.99 | [M+H]+ | 463.123 | 463.1231 | -0.8 | Homoplantaginin | C_22_H_22_O_11_ | CR |
| 201 | 37.99 | [M+H]+ | 463.123 | 463.1231 | -0.8 | Nor-rubrofusarin-6-O-β-D-(6'-O-acetyl ) glucopyranoside | C_22_H_22_O_11_ | CO |
| 202 | 38 | [M+H]+ | 301.071 | 301.0709 | 0.9 | Hydroxygenkwanin | C_16_H_12_O_6_ | CR |
| 203 | 38.00 | [M+H]+ | 301.071 | 301.0709 | 0.9 | Diosmetin | C_16_H_12_O_6_ | RG, CM |
| 204 | 38.07 | [M+FA-H]- | 509.1148 | 509.1192 | 8.6 | Scabioside C | C_18_H_24_O_14_ | RP |
| 205 | 38.28 | [M+FA-H]- | 859.2877 | 859.2897 | 2.3 | Jioglutoside B1 | C_37_H_50_O_20_ | RG |
| 206 | 38.41 | [M+H]+ | 609.181 | 609.1809 | -0.8 | Physcion 8-glucoside | C_28_H_32_O_15_ | CO |
| 207 | 38.42 | [M+H]+ | 517.134 | 517.1335 | -1 | 1,3-Dicaffeoylquinic acid or Isochlorogenic acid A or Isochlorogenic acid C | C_25_H_24_O_12_ | CM |
| 208 | 38.43 | [M+K]+ | 555.09 | 555.0891 | -1.5 | Isochlorogenic acid A | C_25_H_24_O_12_ | BC |
| 209 | 38.99 | [M+H]+ | 255.065 | 255.0654 | 0.9 | Chrysophanol | C_15_H_10_O_4_ | CO |
| 210 | 38.99 | [M+H]+ | 255.065 | 255.0654 | 0.9 | Daidzein | C_15_H_10_O_4_ | LC |
| 211 | 39.02 | [M+H]+ | 435.129 | 435.1285 | -0.3 | Prunin | C_21_H_22_O_10_ | CR |
| 212 | 39.02 | [M+H]+ | 435.129 | 435.1285 | -0.3 | Rubrofusarin-6-O-β-glucoside | C_21_H_22_O_10_ | CO |
| 213 | 39.43 | [M+Na]+ | 661.174 | 661.1735 | -0.6 | Ombuin-3β-rutinoside | C_29_H_34_O_16_ | CM |
| 214 | 39.73 | [M+H]+ | 557.186 | 557.1859 | -1 | Cassitoroside | C_25_H_32_O_14_ | CO |
| 215 | 39.8 | [M-H]- | 509.1664 | 509.1618 | -9.2 | Mudanpioside D | C_24_H_30_O_12_ | RP |
| 216 | 39.82 | [M+FA-H]- | 477.1038 | 477.104 | 0.2 | KaeMpferol-3-O-α-L-rhaMnoside | C_21_H_20_O_10_ | RP |
| 217 | 39.87 | [M+FA-H]- | 539.177 | 539.1772 | 0.3 | Paeonidanin | C_24_H_30_O_11_ | RP |
| 218 | 39.9 | [M+H]+ | 314.139 | 314.1384 | -0.9 | N-trans-Feruloyltyramine | C_18_H_19_NO_4_ | LB |
| 219 | 39.91 | [M+FA-H]- | 965.278 | 965.2787 | 0.7 | Cassiaside B2 or Cassiaside C2 | C_39_H_52_O_25_ | CO |
| 220 | 39.97 | [M-H]- | 312.1241 | 312.1242 | 0.1 | Lyciumide A | C_18_H_19_NO_4_ | LB |
| 221 | 39.97 | [M-H]- | 312.1241 | 312.1242 | 0.1 | N-cis-feruloyltyramine or N-trans-feruloyltyramine | C_18_H_19_NO_4_ | PO |
| 222 | 41.48 | [M+FA-H]- | 623.1618 | 623.1617 | 0 | Apigenin-7-O-neohesperidoside | C_27_H_30_O_14_ | CM |
| 223 | 41.48 | [M+FA-H]- | 623.1618 | 623.1617 | 0 | Rhoifolin | C_27_H_30_O_14_ | CR |
| 224 | 41.48 | [M+FA-H]- | 623.1618 | 623.1617 | 0 | Chrysophanol-1-O-β-gentiobioside | C_27_H_30_O_14_ | CO |
| 225 | 41.74 | [M+FA-H]- | 803.2251 | 803.2253 | 0.2 | Rubrofusatin triglucoside | C_33_H_42_O_20_ | CO |
| 226 | 41.93 | [M+H]+ | 273.076 | 273.0756 | -0.5 | Naringenin | C_15_H_12_O_5_ | CR |
| 227 | 41.93 | [M+H]+ | 417.118 | 417.1187 | 1.6 | Chrysophanol 1-glucoside | C_21_H_20_O_9_ | CO |
| 228 | 41.93 | [M+H]+ | 597.181 | 597.1809 | -0.8 | Eriocitrin | C_27_H_32_O_15_ | CR |
| 229 | 41.93 | [M+H]+ | 597.181 | 597.1809 | -0.8 | Cassiaside C | C_27_H_32_O_15_ | CO |
| 230 | 41.93 | [M+H]+ | 597.181 | 597.1809 | -0.8 | Emodin-1-O-β-gentiobioside or Isorubrofusarin 6-O-beta-gentiobioside | C_27_H_32_O_15_ | CO |
| 231 | 41.96 | [M-H]- | 595.1668 | 595.1663 | -0.9 | Naringenin-6,8-di-C-glucoside | C_27_H_32_O_15_ | CM |
| 232 | 41.96 | [M+FA-H]- | 641.1723 | 641.1727 | 0.6 | Isorubrofusarin-6-O-β-gentiobioside | C_27_H_32_O_15_ | CO |
| 233 | 42.72 | [M-H]- | 637.2138 | 637.2132 | -0.9 | Jionoside D or Leucosceptoside A | C_30_H_38_O_15_ | RG |
| 234 | 43.02 | [M+H]+ | 771.416 | 771.417 | 1.1 | Ophiopogonin Ｒ | C_39_H_62_O_15_ | OJ |
| 235 | 43.08 | [M+K]+ | 663.169 | 663.1672 | -2 | Verbascoside | C_29_H_36_O_15_ | RG |
| 236 | 43.12 | [M-H]- | 687.1931 | 687.1928 | -0.4 | Tricin 4"-O-(threo-βguaiacylglyceryl) Ether⁃7"⁃O⁃β⁃D⁃glucopyranose | C_33_H_36_O_16_ | BC |
| 237 | 43.17 | [M+FA-H]- | 1287.5499 | 1287.5499 | 0 | Polygodoraside C | C_56_H_90_O_30_ | PO |
| 238 | 43.2 | [M+H]+ | 1227.564 | 1227.5656 | 1.3 | Polygodoraside A or Polygodoraside D or Polygodoraside F | C_56_H_90_O_29_ | PO |
| 239 | 43.28 | [M-H]- | 837.4642 | 837.4571 | -8.5 | 4"-O-Acetylsaikosaponin A | C_44_H_70_O_15_ | BC |
| 240 | 43.44 | [M+Na]+ | 513.1 | 513.1009 | 1 | Kaempferol-3-O-acetyl-glucoside | C_23_H_22_O_12_ | CM |
| 241 | 43.58 | [M-H]- | 221.0819 | 221.0819 | -0.4 | 4，7-Dihydroxy-3-butylphthalide | C_12_H_14_O_4_ | LC |
| 242 | 43.69 | [M+FA-H]- | 680.2825 | 680.2865 | 5.9 | (E)-2-(4,5-dihydroxy-2-{3-[(4-hydroxyphenethyl)amino]-3-oxopropyl}phenyl)-3-(4-hydroxy-3,5-dimethoxyphenyl)-N-(4-acetamidobutyl)acrylamide | C_34_H_41_N_3_O_9_ | LB |
| 243 | 43.75 | [M-H]- | 457.0776 | 457.0773 | -0.7 | Epigallocatechin gallate | C_22_H_18_O_11_ | RP |
| 244 | 43.88 | [M+Na]+ | 1235.567 | 1235.559 | -6.3 | Ophiofurospiside C | C_56_H_92_O_28_ | OJ |
| 245 | 43.88 | [M+Na]+ | 1235.567 | 1235.559 | -6.3 | Timosaponin H1 | C_56_H_92_O_28_ | PO |
| 246 | 43.89 | [M+H]+ | 1211.569 | 1211.5688 | -0.3 | Typaspidoside H | C_56_H_90_O_28_ | PO |
| 247 | 43.89 | [M-H]- | 1227.5652 | 1227.5613 | -3.1 | Ophiopogonin H | C_56_H_92_O_29_ | OJ |
| 248 | 43.89 | [M-H]- | 1227.5652 | 1227.5613 | -3.1 | polygonatumoside F | C_56_H_92_O_29_ | PO |
| 249 | 43.9 | [M+H]+ | 917.474 | 917.4744 | 0.4 | Polygoside A | C_45_H_72_O_19_ | PO |
| 250 | 43.93 | [M-H]- | 1079.528 | 1079.5263 | -1.6 | (25S)-26-O-(β-D-glucopyranosyl)-furost-5-en3β,22α,26-triol 3-O-β-D-glucopyranosyl-(1 → 2)-β-D-glucopyranosyl-(1 → 4)-β-D-gluco-pyranoside | C_51_H_84_O_24_ | PO |
| 251 | 44.05 | [M+H]+ | 567.171 | 567.1716 | 1.3 | Cassiaside B | C_26_H_30_O_14_ | CO |
| 252 | 44.06 | [M-H]- | 933.4701 | 933.4695 | -0.6 | Ophiofurospiside F | C_45_H_74_O_20_ | OJ |
| 253 | 44.15 | [M-H]- | 419.0984 | 419.0979 | -1.1 | Cassiaside or Nor-rubrofusarin-6-O-β-D-glucophyranoside | C_20_H_20_O_10_ | CO |
| 254 | 44.22 | [M-H]- | 809.4329 | 809.432 | -1.1 | Saikosaponin X | C_42_H_66_O_15_ | BC |
| 255 | 44.32 | [M+Na]+ | 665.205 | 665.2102 | 7.5 | 6'- O- β- D- glu-copyranosylalbiflorin or Isomaltopaeoniflorin | C_29_H_38_O_16_ | RP |
| 256 | 44.51 | [M+H]+ | 887.463 | 887.4677 | 4.7 | Ophiopojaponin C | C_44_H_70_O_18_ | OJ |
| 257 | 44.54 | [M+FA-H]- | 331.0823 | 331.0804 | -5.9 | Isosakuranetin | C_16_H_14_O_5_ | CR |
| 258 | 44.54 | [M+H]+ | 595.202 | 595.2015 | -1 | Poncirin | C_28_H_34_O_14_ | CR |
| 259 | 44.55 | [M+H]+ | 287.091 | 287.0919 | 1.7 | 5,7,4'-Trihydroxyl homoisoflavanone | C_16_H_14_O_5_ | PO |
| 260 | 44.55 | [M+H]+ | 617.186 | 617.1856 | -1.4 | Mudanpioside H | C_30_H_32_O_14_ | RP |
| 261 | 44.55 | [M+K]+ | 631.142 | 631.1436 | 2 | Buddleoside | C_28_H_32_O_14_ | CM |
| 262 | 44.56 | [M-H]- | 919.4908 | 919.49 | -0.9 | Officinalisnin II | C_45_H_76_O_19_ | PO |
| 263 | 44.75 | [M-H]- | 651.2294 | 651.2284 | -1.6 | Martynoside | C_31_H_40_O_15_ | RG |
| 264 | 44.76 | [M+H]+ | 251.128 | 251.1266 | -4.8 | Dipropylphthalate | C_14_H_18_O_4_ | RP |
| 265 | 44.82 | [M+FA-H]- | 653.1723 | 653.1722 | -0.1 | Diosmin | C_28_H_32_O_15_ | CR |
| 266 | 44.82 | [M-H]- | 939.1109 | 939.1019 | -9.6 | Pentagalloylglucose | C_41_H_32_O_26_ | RP |
| 267 | 44.86 | [M+H]+ | 475.123 | 475.123 | -1 | Apigenin 7-O-acetylglucoside isomer or Apigenin-7-O-6''-acetyl-glucoside | C_23_H_22_O_11_ | CM |
| 268 | 44.91 | [M+H]+ | 285.039 | 285.038 | -4.7 | Rhein | C_15_H_8_O_6_ | CO |
| 269 | 44.92 | [M+H]+ | 285.076 | 285.0757 | -0.1 | Obtusifolin | C_16_H_12_O_5_ | CO |
| 270 | 44.92 | [M+H]+ | 447.129 | 447.1273 | -2.8 | Acacetin-7-O-galactoside | C_22_H_22_O_10_ | CM |
| 271 | 44.92 | [M-H]- | 491.1195 | 491.1184 | -2.2 | Aurantio-obtusin-6-O-β-D-glucoside or Gluco-aurantio-obtusin | C_23_H_24_O_12_ | CO |
| 272 | 44.92 | [M+FA-H]- | 491.1195 | 491.1184 | -2.2 | Physcion-8-O-β-glucoside | C_22_H_22_O_10_ | CO |
| 273 | 44.93 | [M+FA-H]- | 315.051 | 315.0491 | -6.1 | Emodin | C_15_H_10_O_5_ | LB, CO, PO |
| 274 | 44.97 | [M-H]- | 1195.5753 | 1195.5749 | -0.3 | Ophiopogonin F or Ophiopogonin G or Ophiopogonin J | C_56_H_92_O_27_ | OJ |
| 275 | 44.97 | [M-H]- | 1241.5808 | 1241.5782 | -2.1 | Polygodoraside H | C_57_H_94_O_29_ | PO |
| 276 | 44.99 | [M-H]- | 269.0455 | 269.0453 | -0.8 | Apigenin | C_15_H_10_O_5_ | CM |
| 277 | 45.13 | [M+FA-H]- | 1109.5385 | 1109.537 | -1.4 | Ophiofurospiside K | C_51_H_84_O_23_ | OJ |
| 278 | 45.16 | [M+H]+ | 739.426 | 739.4256 | -1 | Dracaenoside F | C_39_H_62_O_13_ | OJ |
| 279 | 45.16 | [M+K]+ | 761.387 | 761.3856 | -2.3 | Ophiopogonin C' | C_39_H_62_O_12_ | OJ |
| 280 | 45.16 | [M-H]- | 1211.5702 | 1211.5674 | -2.4 | Ophiopogonin N or Ophiopogonin Q | C_56_H_92_O_28_ | OJ |
| 281 | 45.16 | [M-H]- | 1257.5757 | 1257.5745 | -1 | Polygodoraside G | C_57_H_94_O_30_ | PO |
| 282 | 45.17 | [M-H]- | 355.1187 | 355.1185 | -0.5 | Coniferyl ferulate | C_20_H_20_O_6_ | AS |
| 283 | 45.25 | [M+Cl]- | 219.0066 | 219.0066 | 0 | 3-Methoxygallic acid | C_8_H_8_O_5_ | RP |
| 284 | 45.27 | [M+H]+ | 447.222 | 447.2211 | -3 | (Z)-(1S,5R)-β-pinen-10-yl-β-vicianoside | C_21_H_34_O_10_ | RP |
| 285 | 45.43 | [M+Cl]- | 659.2166 | 659.21 | -9.9 | 3-Benzofurancarboxamide or Cannabisin D or Lyciumamide B | C_36_H_36_N_2_O_8_ | LB |
| 286 | 45.45 | [M-H]- | 581.224 | 581.2235 | -0.9 | (+)-Lyoniresinol-3α-O-β-D-glucopyranoside | C_28_H_38_O_13_ | LB |
| 287 | 45.54 | [M+H]+ | 507.15 | 507.149 | -1.4 | 2-Gluco- chrysoobtusin | C_24_H_26_O_12_ | CO |
| 288 | 45.55 | [M+K]+ | 545.106 | 545.1069 | 2.5 | Vittariflavone | C_24_H_26_O_12_ | BC |
| 289 | 45.55 | [M+FA-H]- | 551.1406 | 551.1402 | -0.8 | Obtusin | C_24_H_26_O_12_ | CO |
| 290 | 45.57 | [M-H]- | 581.1512 | 581.1507 | -0.8 | Nor-rubrofusarin or Rubrofusarin-6-O-β-gentiobioside | C_26_H_30_O_15_ | CO |
| 291 | 45.59 | [M+FA-H]- | 675.1931 | 675.1928 | -0.4 | Mudanpioside B | C_31_H_34_O_14_ | RP |
| 292 | 45.7 | [M+H]+ | 625.254 | 625.2604 | 9.5 | Cannabisin F | C_36_H_36_N_2_O_8_ | LB |
| 293 | 45.7 | [M+FA-H]- | 1255.5601 | 1255.5598 | -0.2 | Polygodoraside E | C_56_H_90_O_28_ | PO |
| 294 | 46 | [M-H]- | 490.1871 | 490.1868 | -0.7 | grossamide K or Lyciumamide C | C_28_H_29_NO_7_ | LB |
| 295 | 46.01 | [M+FA-H]- | 1239.5652 | 1239.5538 | -9.1 | Typaspidoside L | C_56_H_90_O_27_ | PO |
| 296 | 46.06 | [M+Cl]- | 649.1693 | 649.1629 | -9.9 | Mudanpioside A | C_31_H_34_O_13_ | RP |
| 297 | 46.07 | [M-H]- | 797.4693 | 797.4669 | -3 | Hydroxy-saikosaponin A or Hydroxy-saikosaponin D | C_42_H_70_O_14_ | BC |
| 298 | 46.10 | [M+FA-H]- | 435.2236 | 435.2234 | -0.4 | Rehmaionoside A | C_19_H_34_O_8_ | RG |
| 299 | 46.14 | [M+H]+ | 329.232 | 329.2329 | 2 | Lipoxin | C_18_H_32_O_5_ | LB |
| 300 | 46.25 | [M+H]+ | 533.129 | 533.1308 | 3.5 | 3,4-Dicaffeoylquinic acid | C_25_H_24_O_13_ | CM |
| 301 | 46.35 | [M+H]+ | 189.091 | 189.0909 | -0.8 | (E)-Butylidenephthalide or (Z)-Butylidenephthalide | C_12_H_12_O_2_ | AS |
| 302 | 46.35 | [M+H]+ | 189.091 | 189.0909 | -0.8 | Ligustrazine or n-Butylidenephthalide | C_12_H_12_O_2_ | AS |
| 303 | 46.35 | [M+H]+ | 189.091 | 189.0909 | -0.8 | Z-butylidenephthalide | C_12_H_12_O_2_ | LC |
| 304 | 46.36 | [M-H]- | 205.087 | 205.0868 | -0.9 | 7-Epoxyligustilide or Senkyunolide F | C_12_H_14_O_3_ | LC |
| 305 | 46.36 | [M-H]- | 205.087 | 205.0868 | -0.9 | Senkyunolide F | C_12_H_14_O_3_ | AS |
| 306 | 46.44 | [M+FA-H]- | 655.3699 | 655.3694 | -0.8 | Polygonatumoside G | C_33_H_54_O_10_ | PO |
| 307 | 46.6 | [M+H]+ | 191.107 | 191.1066 | -0.5 | Butylphthalide or E-Ligustilide or Z-Ligustilide | C_12_H_14_O_2_ | AS |
| 308 | 46.6 | [M+H]+ | 191.107 | 191.1066 | -0.5 | Butylphthalide or Z-ligustilide | C_12_H_14_O_2_ | LC |
| 309 | 46.61 | [M-H]- | 207.1027 | 207.1026 | -0.5 | Senkyunolide K | C_12_H_16_O_3_ | LC |
| 310 | 46.8 | [M-H]- | 680.2672 | 680.2626 | -6.8 | Lycibarbarspermidine D | C_31_H_43_N_3_O_14_ | LB |
| 311 | 47.29 | [M+Cl]- | 319.059 | 319.0601 | 3.4 | Emodin-8-O-β-D-glucoside | C_13_H_16_O_7_ | RP |
| 312 | 47.37 | [M+H]+ | 331.081 | 331.081 | -0.7 | 3,7-Di-O-methylquercetin | C_17_H_14_O_7_ | BC |
| 313 | 47.37 | [M+H]+ | 331.081 | 331.081 | -0.7 | Aurantioobtusin | C_17_H_14_O_7_ | CO |
| 314 | 47.38 | [M-H]- | 329.0667 | 329.0664 | -0.9 | 1-Desmethylobtusin | C_17_H_14_O_7_ | CO |
| 315 | 47.49 | [M-H]- | 191.1078 | 191.1075 | -1.1 | Senkyunolide A | C_12_H_16_O_2_ | LC |
| 316 | 47.49 | [M-H]- | 191.1078 | 191.1075 | -1.1 | Senkyunolide A | C_12_H_16_O_2_ | AS |
| 317 | 47.67 | [M+Na]+ | 759.19 | 759.1903 | 1 | Paeonin D | C_37_H_36_O_16_ | RP |
| 318 | 47.68 | [M+H]+ | 317.066 | 317.0656 | 0 | Isorhamnetol | C_16_H_12_O_7_ | CR |
| 319 | 47.7 | [M+FA-H]- | 255.1238 | 255.1237 | -0.2 | Sedanonic acid | C_12_H_18_O_3_ | LC |
| 320 | 47.8 | [M-H]- | 283.2643 | 283.2657 | 5 | Ethyl palmitate | C_18_H_36_O_2_ | RP |
| 321 | 47.81 | [M+H]+ | 195.138 | 195.1381 | 1 | cnidilide | C_12_H_18_O_2_ | LC |
| 322 | 48.08 | [M-H]- | 373.1293 | 373.129 | -0.8 | 5,7,8,3’,4’-Pentamethoxyflavanone | C_20_H_22_O_7_ | CR |
| 323 | 48.15 | [M+FA-H]- | 373.0929 | 373.0928 | -0.2 | 3-Hydroxy-5,7,8-trimethoxyflavone | C_18_H_16_O_6_ | CR |
| 324 | 48.16 | [M+Na]+ | 351.084 | 351.0843 | 1.2 | Ophiopogonanoe A | C_18_H_16_O_6_ | OJ |
| 325 | 48.41 | [M+FA-H]- | 694.2829 | 694.2828 | -0.1 | Lycibarbarspermidine B | C_31_H_43_N_3_O_12_ | LB |
| 326 | 48.52 | [M+Cl]- | 411.1063 | 411.1075 | 2.7 | 8-Debenzoylpaeoniflorin | C_16_H_24_O_10_ | RP |
| 327 | 48.57 | [M+H]+ | 315.086 | 315.0867 | 1.2 | 5,7,3’,4’-Tetramethoxyflavone | C_17_H_14_O_6_ | CR |
| 328 | 48.63 | [M-H]- | 493.1199 | 493.118 | -3.8 | 6' -O-galloylsucrose | C_19_H_26_O_15_ | RP |
| 329 | 48.64 | [M-H]- | 203.0714 | 203.0712 | -0.9 | (Z)-3-Butylidene-7-hydroxyphthalide or Senkyunolide C | C_12_H_12_O_3_ | AS |
| 330 | 48.64 | [M-H]- | 203.0714 | 203.0712 | -0.9 | 3-Butylidene-7-hydroxyphthalide | C_12_H_12_O_3_ | LC |
| 331 | 48.66 | [M+Cl]- | 397.1271 | 397.1295 | 6.1 | 6-O-copyranosyl-lactinolide | C_16_H_26_O_9_ | RP |
| 332 | 48.74 | [M-H]- | 331.0671 | 331.0654 | -5.2 | 6-O-galloylsucrose | C_13_H_16_O_10_ | RP |
| 333 | 48.76 | [M-H]- | 299.0925 | 299.0924 | -0.4 | (3R)-5,7-Dihydroxyl-6-methyl-3-(4'-hydroxylbenzyl)-chroman-4-one | C_17_H_16_O_5_ | PO |
| 334 | 48.76 | [M-H]- | 299.0925 | 299.0924 | -0.4 | 5,7,2'-trihydroxy-8-methyl-3-(3', 4'-methylenedioxybenzyl) chromone | C_17_H_16_O_5_ | OJ |
| 335 | 48.83 | [M+FA-H]- | 359.0772 | 359.0752 | -5.7 | Pectolinarigenin | C_17_H_14_O_6_ | CR |
| 336 | 49.07 | [M-H]- | 373.0929 | 373.0926 | -0.9 | Casticin | C_19_H_18_O_8_ | CM |
| 337 | 49.09 | [M+H]+ | 331.118 | 331.1176 | 0 | (3R)-5,7-Dihydroxyl-6-methyl-8-methoxyl-3-(4'-hydroxylbenzyl)-chroman-4-one | C_18_H_18_O_6_ | PO |
| 338 | 49.1 | [M+Na]+ | 427.136 | 427.1369 | 1.2 | 5,6,7,8,3',4'-Hexamethoxyflavanone | C_21_H_24_O_8_ | CR |
| 339 | 49.25 | [M+H]+ | 359.113 | 359.1129 | 1.1 | 5-Hydroxy-7,8,3’,4’-tetramethoxyflavone | C_19_H_18_O_7_ | CR |
| 340 | 49.25 | [M+H]+ | 359.113 | 359.1129 | 1.1 | Pureonebio | C_19_H_18_O_7_ | CO |
| 341 | 49.31 | [M+H]+ | 403.139 | 403.1372 | -3.8 | 5,6,7,3’,4’,5’-Hexamethoxyflavone | C_21_H_22_O_8_ | CR |
| 342 | 49.41 | [M+Na]+ | 335.089 | 335.0908 | 5.5 | 5,7,4’-Trimethoxyflavone | C_18_H_16_O_5_ | CR |
| 343 | 49.46 | [M+H]+ | 343.118 | 343.1171 | -1.6 | 5,7,8,4’-Tetramethoxyflavone | C_19_H_18_O_6_ | CR |
| 344 | 49.46 | [M+K]+ | 381.095 | 381.0975 | 7.7 | 4,9-Dihydroxy-8,10-dehydrothymol-1-O-β-D-glucoside | C_16_H_22_O_8_ | RP |
| 345 | 49.47 | [M+K]+ | 381.073 | 381.0738 | 0.8 | Methylophiopogonanone A | C_19_H_18_O_6_ | OJ |
| 346 | 49.59 | [M-H]- | 753.4067 | 753.4067 | 0 | Ophiopogonin Ra | C_39_H_62_O_14_ | OJ |
| 347 | 49.62 | [M+FA-H]- | 710.2778 | 710.2758 | -2.8 | Lycibarbarspermidine C | C_31_H_43_N_3_O_13_ | LB |
| 348 | 49.67 | [M-H]- | 313.1081 | 313.1081 | -0.1 | (3R)-5,7-Dihydroxy-6,8-dimethyl-3-(4′-hydroxybenzyl)-chroman-4-one | C_18_H_18_O_5_ | PO |
| 349 | 49.67 | [M-H]- | 313.1081 | 313.1081 | -0.1 | Ophiopogonanoe B | C_18_H_18_O_5_ | OJ |
| 350 | 50.01 | [M+K]+ | 317.115 | 317.1123 | -8.5 | Senkyunolide L | C_16_H_22_O_4_ | LC |
| 351 | 50.01 | [M+K]+ | 471.105 | 471.106 | 1.7 | 3,5,6,7,8,3',4'-Heptamethoxyflavone | C_22_H_24_O_9_ | CR |
| 352 | 50.04 | [M+Cl]- | 381.0747 | 381.0756 | 2.5 | 5,7, 2',4'-tetradihydroxy-8-methoyl-6-methyl-homoisoflavanone | C_18_H_18_O_7_ | OJ |
| 353 | 50.04 | [M+Cl]- | 381.0747 | 381.0756 | 2.5 | 5,7,2',4'-Tetrahydroxy-6-methoxy-8-methyl homoisoflavanone | C_18_H_18_O_7_ | PO |
| 354 | 50.04 | [M+Cl]- | 1049.5093 | 1049.5017 | -7.2 | malonyl-saikosaponin F | C_51_H_82_O_20_ | BC |
| 355 | 50.06 | [M-H]- | 343.0823 | 343.0819 | -1.4 | Eupatorin | C_18_H_16_O_7_ | CM |
| 356 | 50.06 | [M+Cl]- | 379.059 | 379.0602 | 3 | Eupatilin | C_18_H_16_O_7_ | CR |
| 357 | 50.24 | [M+H]+ | 455.352 | 455.352 | 0.1 | Mudanpinoicacid A | C_30_H_46_O_3_ | RP |
| 358 | 50.24 | [M+K]+ | 819.429 | 819.4296 | 0.5 | Saikosaponin B1 or Saikosaponin D | C_42_H_68_O_13_ | BC |
| 359 | 50.29 | [M-H]- | 283.0612 | 283.0611 | -0.3 | 7-dihydroxy-3-(4'-hydroxybenzyl) chromone-4-one | C_16_H_12_O_5_ | OJ |
| 360 | 50.29 | [M-H]- | 283.0612 | 283.0611 | -0.3 | Acacetin | C_16_H_12_O_5_ | CM |
| 361 | 50.29 | [M+Cl]- | 395.0903 | 395.0906 | 0.8 | Ophiopogonanone E | C_19_H_20_O_7_ | OJ |
| 362 | 50.3 | [M-H]- | 359.1136 | 359.1136 | 0 | 3-Hydroxy-5,6,7,4’-tetramethoxyflavanone | C_19_H_20_O_7_ | CR |
| 363 | 50.32 | [M-H]- | 811.4849 | 811.4869 | 2.5 | Saikochrome A or Saikosaponin B3 | C_43_H_72_O_14_ | BC |
| 364 | 50.33 | [M+H]+ | 419.134 | 419.1337 | 0.1 | 3- Hydroxy  -5,6,7,8,3’,4’-hexamethoxyflavone | C_21_H_22_O_9_ | CR |
| 365 | 50.36 | [M+Cl]- | 336.9757 | 336.9734 | -6.7 | Ellagic acid | C_14_H_6_O_8_ | RP |
| 366 | 50.4 | [M+FA-H]- | 329.0667 | 329.066 | -2.1 | Emodin-3-methyl ether | C_16_H_12_O_5_ | CO |
| 367 | 50.4 | [M+FA-H]- | 329.0667 | 329.066 | -2.1 | Physcion | C_16_H_12_O_5_ | PO |
| 368 | 50.46 | [M-H]- | 911.501 | 911.5006 | -0.4 | Chikusaikoside I | C_47_H_76_O_17_ | BC |
| 369 | 50.49 | [M+Na]+ | 967.524 | 967.5159 | -8.1 | Hydroxy-saikosaponin C or Saikosaponin N | C_48_H_80_O_18_ | BC |
| 370 | 50.53 | [M+H]+ | 373.128 | 373.1273 | -2.4 | isosinensetin | C_20_H_20_O_7_ | CR |
| 371 | 50.53 | [M+K]+ | 411.084 | 411.0839 | -0.4 | 5,7,8,3',4'-Pentamethoxyflavone | C_20_H_20_O_7_ | CR |
| 372 | 50.59 | [M+H]+ | 552.223 | 552.223 | 0.3 | (3S,4R)-6-hydroxy-4-(4-hydroxy-3,5-dimethoxyphenyl)-3-(hydroxymethyl)-N-(4-hydroxyphenethyl)-5,7-dimethoxy-3,4-dihydronaphthalene-2-carboxamide | C_30_H_33_NO_9_ | LB |
| 373 | 50.62 | [M-H]- | 1049.5174 | 1049.5083 | -8.7 | Ophiofurospiside A or Ophiofurospiside L | C_50_H_82_O_23_ | OJ |
| 374 | 50.62 | [M+FA-H]- | 1095.5229 | 1095.5137 | -8.4 | Ophiopogonin I | C_50_H_82_O_23_ | OJ |
| 375 | 50.74 | [M+FA-H]- | 1057.5225 | 1057.5126 | -9.4 | Malonyl-saikosaponin C | C_51_H_80_O_20_ | BC |
| 376 | 50.87 | [M+H]+ | 797.468 | 797.4677 | -0.6 | Saikosaponin I | C_42_H_68_O_14_ | BC |
| 377 | 50.99 | [M+H]+ | 839.479 | 839.4797 | 1.1 | 4"-O-Acetylsaikosaponin D | C_44_H_70_O_15_ | BC |
| 378 | 51.09 | [M-H]- | 915.4595 | 915.4588 | -0.8 | Polygoside B | C_45_H_72_O_19_ | PO |
| 379 | 51.19 | [M+K]+ | 427.079 | 427.0796 | 1.4 | C16 or C23 | C_20_H_20_O_8_ | CR |
| 380 | 51.23 | [M+FA-H]- | 973.5378 | 973.5296 | -8.4 | Saikosaponin F | C_48_H_80_O_17_ | BC |
| 381 | 51.24 | [M-H]- | 925.5166 | 925.5155 | -1.2 | Saikosaponin C | C_48_H_78_O_17_ | BC |
| 382 | 51.33 | [M-H]- | 737.4118 | 737.4121 | 0.4 | Tb | C_39_H_62_O_13_ | OJ |
| 383 | 51.67 | [M+H]+ | 942.518 | 942.516 | -2.4 | Saikosaponin S | C_48_H_77_O_18_ | BC |
| 384 | 51.73 | [M+Na]+ | 317.172 | 317.1735 | 3.6 | 6-Gingerol | C_17_H_26_O_4_ | BC |
| 385 | 51.94 | [M+K]+ | 861.44 | 861.4401 | 0.4 | 23-O-acetylsaikosaponin-A or 3"-O-acetylsaikosaponin A or 6"-O-acetylsaikosaponin A | C_44_H_70_O_14_ | BC |
| 386 | 51.98 | [M-H]- | 860.4501 | 860.4476 | -2.9 | Ammonium Glycyrrhizinate | C_42_H_62_O_16_._3_H_2_O.H_3_N | BC |
| 387 | 52.24 | [M-H]- | 763.4638 | 763.4647 | 1.2 | Saikosaponin E | C_42_H_68_O_12_ | BC |
| 388 | 52.26 | [M+FA-H]- | 941.5115 | 941.511 | -0.6 | Rotundifolioside I | C_47_H_76_O_16_ | BC |
| 389 | 52.36 | [M-H]- | 865.4591 | 865.4587 | -0.5 | Malonyl-saikosaponin A or Tartronoyl-saikosaponin D | C_45_H_70_O_16_ | BC |
| 390 | 52.51 | [M+Cl]- | 507.3247 | 507.3281 | 6.8 | Hederagenin | C_30_H_48_O_4_ | RP |
| 391 | 52.87 | [M+Na]+ | 363.084 | 363.0843 | 1 | Methylophiopogonone A | C_19_H_16_O_6_ | OJ |
| 392 | 53.1 | [M+H]+ | 381.206 | 381.2065 | 1.3 | Angelicide or Levistilide A or Riligustilide or Senkyunolide O or Senkyunolide P | C_24_H_28_O_4_ | AS |
| 393 | 53.1 | [M+H]+ | 381.206 | 381.2065 | 1.3 | Levistolide A or Tokinolide B | C_24_H_28_O_4_ | LC |
| 394 | 53.55 | [M+K]+ | 819.393 | 819.3952 | 3 | Ophiopogonin P | C_41_H_64_O_14_ | OJ |
| 395 | 53.82 | [M-H]- | 327.1238 | 327.1238 | 0 | Lophiopogonanone B or Methylophiopogonanone B | C_19_H_20_O_5_ | OJ |
| 396 | 53.99 | [M+K]+ | 893.43 | 893.428 | -1.7 | Ophiopogonin D' | C_44_H_70_O_16_ | OJ |
| 397 | 54.02 | [M-H]- | 853.4591 | 853.4596 | 0.6 | Ophiopogonin D | C_44_H_70_O_16_ | OJ |
| 398 | 54.05 | [M-H]- | 263.1653 | 263.1655 | 1 | Senkyunolide N | C_16_H_24_O_3_ | LC |
| 399 | 55.08 | [M-H]- | 721.4169 | 721.4183 | 2.1 | Ophiopogonin B | C_39_H_62_O_12_ | OJ |
| 400 | 55.09 | [M-H]- | 617.4059 | 617.4044 | -2.4 | Acetyl-prosaikogenin D or Prosaikogenin A or Prosaikogenin D | C_36_H_58_O_8_ | BC |
| 401 | 55.09 | [M-H]- | 617.4059 | 617.4044 | -2.4 | Prosaikogenin G | C_36_H_58_O_8_ | BC |
| 402 | 57.15 | [M+H]+ | 205.086 | 205.0859 | -0.1 | Senkyunolide B or Senkyunolide E | C_12_H_12_O_3_ | AS |
| 403 | 58.86 | [M+H]+ | 621.4 | 621.3994 | -0.5 | CiMigenol 3-beta-D-xylopyranoside | C_35_H_56_O_9_ | AA |
| 404 | 59.6 | [M-H]- | 305.1507 | 305.1491 | -5.2 | N-acetyl-N'-trans-feruloylputrescine | C_16_H_22_N_2_O_4_ | LB |
| 405 | 60.04 | [M-H]- | 455.3531 | 455.3533 | 0.5 | Oleanolic acid | C_30_H_48_O_3_ | RG |
| 406 | 60.08 | [M+FA-H]- | 323.2228 | 323.221 | -5.4 | Linolenic acid | C_18_H_30_O_2_ | LB |
| 407 | 60.78 | [M+Na]+ | 285.146 | 285.1462 | 0.2 | Lycifuranone A | C_16_H_22_O_3_ | LB |
| 408 | 60.79 | [M+H]+ | 281.248 | 281.2477 | 0.8 | Linoleic acid | C_18_H_32_O_2_ | LB |
| 409 | 60.88 | [M-H]- | 417.1555 | 417.1533 | -5.2 | 2,6,2',6'-Tetramethoxy-4,4'-bis(2,3-epoxy-hydroxypropyl)biphenyl | C_22_H_26_O_8_ | LB |
| 410 | 61.53 | [M+FA-H]- | 327.2541 | 327.2549 | 2.5 | Oleic acid | C_18_H_34_O_2_ | LB |
| 411 | 62.24 | [M+Na]+ | 591.417 | 591.4167 | -0.9 | Zeaxanthin | C_40_H_56_O_2_ | LB |
| 412 | 62.35 | [M+H]+ | 167.034 | 167.0341 | 1.4 | Phthalic acid | C_8_H_6_O_4_ | AS |
| 413 | 62.43 | [M+H]+ | 577.446 | 577.4463 | 0.1 | Daucossterol | C_35_H_60_O_6_ | AA |
| 414 | 65.38 | [M+FA-H]- | 1153.6011 | 1153.6083 | 6.2 | Ginsenoside Rb1 | C_54_H_92_O_23_ | BC |
| 415 | 65.4 | [M-H]- | 777.6191 | 777.6249 | 7.4 | Zeaxanthin monomyristate | C_54_H_82_O_3_ | LB |

Fig. S1 The chemical structures of identified constituents in YGMM

 Fig. S1 The chemical structures of identified constituents in YGMM(Continued)

 Fig. S1 The chemical structures of identified constituents in YGMM(Continued)

Fig. S2 The possible fragament pathway of 3-O-feruloylquinic acid

Fig.S3 The possible fragament pathway of quercetin

Fig.S4 The possible fragament pathway of 5,6,7,8,3',4'-Hexamethoxyflavanone

Fig.S5 The possible fragament pathway of Z-Ligustilide

Fig.S6 The possible fragament pathway of paeoniflorin

 Fig.S7 The possible fragament pathway of polygoside A

Fig.S8 The possible fragament pathway of emodin-3-methyl ether
